# A nonenzymatic method for cleaving polysaccharides to yield oligosaccharides for structural analysis

**DOI:** 10.1038/s41467-020-17778-1

**Published:** 2020-08-07

**Authors:** Matthew J. Amicucci, Eshani Nandita, Ace G. Galermo, Juan Jose Castillo, Siyu Chen, Dayoung Park, Jennifer T. Smilowitz, J. Bruce German, David A. Mills, Carlito B. Lebrilla

**Affiliations:** 1 Agricultural and Environmental Chemistry Graduate Group, University of California, Davis, CA USA; 2 Department of Chemistry, University of California, Davis, CA USA; 3 Foods For Health Institute, University of California, Davis, CA USA; 4 Department of Food Science and Technology, University of California, Davis, CA USA; 5 Department of Biochemistry and Molecular Medicine, University of California, Davis, CA USA

**Keywords:** Polysaccharides, Glycomics, Mass spectrometry

## Abstract

Polysaccharides are the most abundant biomolecules in nature, but are the least understood in terms of their chemical structures and biological functions. Polysaccharides cannot be simply sequenced because they are often highly branched and lack a uniform structure. Furthermore, large polymeric structures cannot be directly analyzed by mass spectrometry techniques, a problem that has been solved for polynucleotides and proteins. While restriction enzymes have advanced genomic analysis, and trypsin has advanced proteomic analysis, there has been no equivalent enzyme for universal polysaccharide digestion. We describe the development and application of a chemical method for producing oligosaccharides from polysaccharides. The released oligosaccharides are characterized by advanced liquid chromatography–mass spectrometry (LC–MS) methods with high sensitivity, accuracy and throughput. The technique is first used to identify polysaccharides by oligosaccharide fingerprinting. Next, the polysaccharide compositions of food and feces are determined, further illustrating the utility of technique in food and clinical studies.

## Abstract (web summary)

While mass spectrometry-based proteomics largely relies on digesting proteins into peptides, there is no equivalent strategy for polysaccharide analysis. Here, the authors develop a chemical approach to break down poly- into oligosaccharides and present a workflow to identify polysaccharides by oligosaccharide fingerprinting.

## Introduction

The fundamental importance of carbohydrates to biological organisms remains discouragingly elusive. The plants that nourish all life forms and stabilize the planet’s atmosphere, are mostly carbohydrate. Yet scientists struggle to provide even the most crude predictions of how the complexity of polysaccharide structures drives their endogenous functions much less their roles in fueling the entire food web. Epidemiologic and observational studies have tentatively documented that polysaccharides (as fiber) are a vitally important dimension of nutrition and health^1–4^. But investigations of the detailed structural basis of those functions is ostensibly nonexistent. Understanding the structure/function relationships of polysaccharides requires that their structures be known. They are not. The development of analytical tools for characterizing polysaccharides has lagged far behind than those of DNA and proteins^5^. Polysaccharide analysis remains slow and tedious, as current strategies still employ methods developed long before the development of high throughput structural tools commonly used in genomic and proteomic analysis^6–8^. Unfortunately, these limitations in determining polysaccharide structures hinder our ability to understand their fundamental biological roles in fields from fuel efficiency to human health. The deficiency in structural methods for polysaccharides is becoming particularly acute in foods where the polysaccharide compositions represent a major gap in knowledge and are rarely, if ever, identified or quantified^9^.

The dissociation of polysaccharides into constitutive and measurable oligosaccharides has been the missing critical component for characterizing polysaccharides. Robust digestion methods are the key to polysaccharide analysis, which opens polysaccharides to the most powerful modern chromatographic, mass spectrometry, computational, and robotic techniques^10–14^. In proteomic analysis, the ability to generate oligopeptides was a necessary first-step toward high throughput analysis. For proteomic analysis, proteases such as trypsin yielded highly reproducible cleavages and produced peptides of sufficient length to identify the parent protein. The methods involved the matching of peptide masses to databases for peptide fingerprinting^15–17^, and the use of tandem mass spectrometry to obtain sequence-specific fragments for protein identification^18^. However, the analogy between protein and polysaccharide sequencing must end here. Unlike proteins and DNA, which have discrete and linear primary structures, polysaccharide structures may be composed of homo or copolymers with stochastically distributed monomers or pendant polymers each with distinct monosaccharide compositions with various glycosidic linkages. To complicate the primary structures further, branching may occur with no distinct length of polymer. Due to the large number of variable structural attributes, polysaccharides do not have precise structures and must be thought of as probabilistic distributions of their innate structural features. The lack of a unified structure further means that there is no enzymatic process, or a trypsin analog, to produce oligosaccharides from most polysaccharides. Instead, there are enzymes such as amylase that produce monosaccharides and oligosaccharides from only specific polysaccharides (amylose and amylopectin). Similarly, animal polysaccharide such as glycosaminoglycans (GAGs) are routinely digested to disaccharides via enzymatic approaches for further analysis by liquid chromatography–mass spectrometry (LC–MS)^19–23^. Due to established and robust methods, GAGs were not explored in this report. The lack of a single unified polymeric structure further obviates the use of strategies that aim to reassemble parent polymer sequence from aligning overlapping oligomer sequences. Rather, polysaccharide sequence should be used to understand the frequency that individual structural features (branching, linkages between individual monosaccharides, and modifications) are found in a distribution of similar but, often, structurally unique molecules.

The structural heterogeneity of polysaccharides is due to the large diversity in monosaccharide and linkage compositions. Thus, there is no universal digestive enzyme for producing oligosaccharides from polysaccharides. The repertoire of natural glycosidase enzymes is conspicuous by their high specificity for specific glycosidic bonds. Therefore, only a limited number of polysaccharide linkages can be digested with a specific enzyme^24,25^. Acid-based techniques have been used to produce oligosaccharides from polysaccharides with varying levels of success, but generally require precise reaction conditions for each polysaccharide, and is therefore not well suited for the analysis of complicated mixtures or heterogeneous structures^26–28^.

The combination of complexity of polysaccharide structures and specificity of glycosidase catalysis is central to biology itself. Fuel and carbon flows through all ecosystems largely as carbohydrate. Managing these flows for any purpose from energy efficiency and sustainability to human metabolism and health requires that the structures of all biopolymers that contain carbohydrate to be known. The need for more robust polysaccharide characterization techniques is especially crucial for characterizing food polysaccharides. Carbohydrates make up the largest part of most human diets, which are now associated with various metabolic diseases^29,30^ and are the largest driver of the gut microbial population^31–35^. While there has been rapid progress in characterizing the microbial composition of the gut microbiome^36,37^, similar advances have not been made in characterizing the carbohydrates that modulate those populations^9^. Thus, the impact that dietary carbohydrates have on the gut microbiome is, generally, indirectly measured by genomic and transcriptomic techniques rather than through direct carbohydrate analysis, which continues to hinder our mechanistic understanding of this complicated process^38–40^. Therefore, there is an immediate and pressing need to expand the current toolbox of polysaccharide characterization if we are to better understand diet–microbe interactions^31,41–46^.

In this report, we describe a chemical method for the dissociation of diverse polysaccharides into oligosaccharides. Fenton’s initiation toward defined oligosaccharide groups (FITDOG) employs oxidative chemistry to disassemble structurally diverse polysaccharides into oligosaccharides that are of sufficient length to be structurally probed by LC–MS.

## Results

### Optimization of reaction conditions

**Fig. 1:**
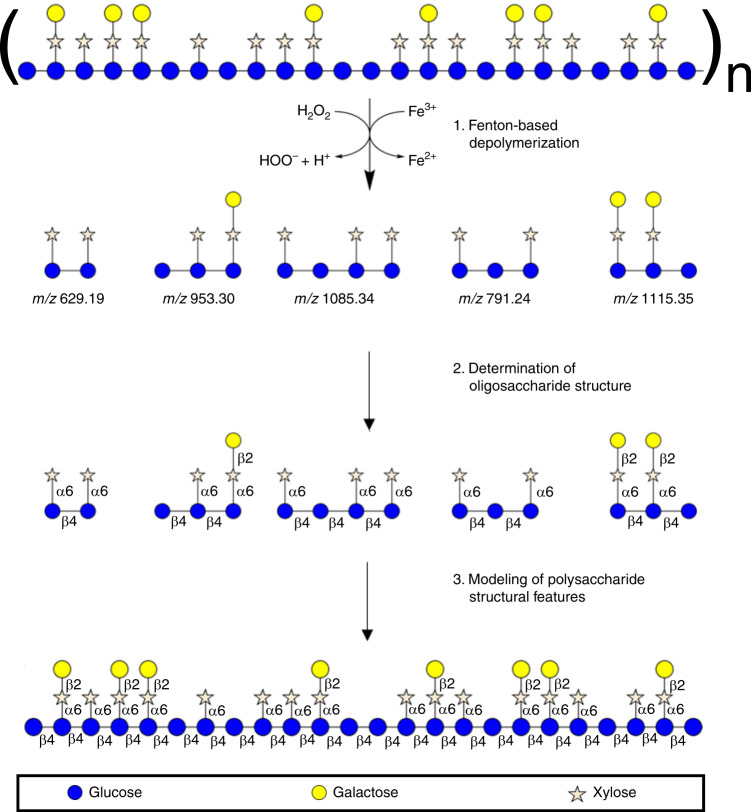
Oxidative method of polysaccharide depolymerization. FITDOG reaction illustrating the dissociation of xyloglucan polysaccharide into representative oligosaccharides and its reconstruction by reassembly of the elucidated oligosaccharides.

The FITDOG process was developed to produce oligosaccharides from polysaccharides so that advanced separation and mass spectrometry methods could be employed for analysis. The process was initiated by a reaction between a metal catalyst, Fe^3+^, and an oxidizing agent, hydrogen peroxide, to produce reactive radical species that cleave glycosidic bonds. The radicals induce oxidative cleavage of the polysaccharide backbone to produce oligosaccharides that are representative of the parent polysaccharide structure (Fig. 1). An LC–MS/MS approach employing collision induced dissociation (CID) was used to obtain structural information.

A considerable effort was placed in developing a general reaction condition suitable for a large number of structural variations. These conditions included the relative amounts of reagents and analytes, reaction conditions, and time. Over oxidation was an issue that needed to be controlled. The conditions presented herein represent the best conditions for a large number of different polysaccharides including those in complicated matrices such as food.

### Production of oligosaccharides by FITDOG

**Fig. 2:**
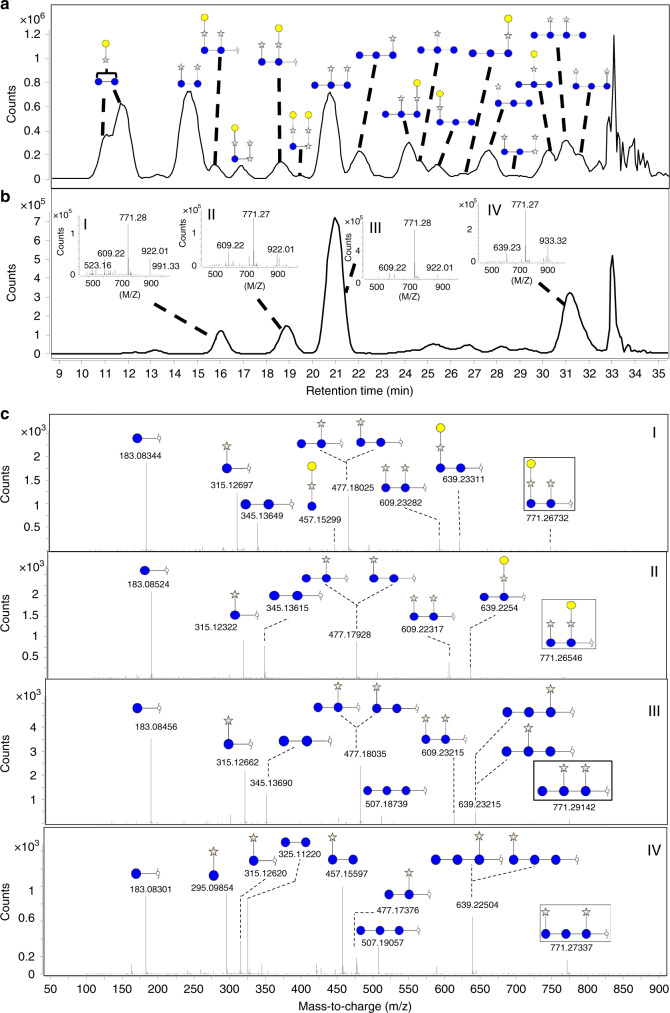
Characterization of FITDOG derived xyloglucan oligosaccharides. **a** Annotated base peak chromatogram of characterized xyloglucan-derived oligosaccharides. **b** Extracted ion chromatogram of *m*/*z* 771.27 shows four isomers. **c** Annotated tandem mass spectra of the four *m*/*z* 771.27 isomers and their final elucidated structures.

Polysaccharides with known structures were used to determine the full capabilities and characteristics of the method. For example, xyloglucan is comprised of a β(1 → 4) glucose backbone with frequent α(1 → 6) xylose branches that are often terminated with a single β(1 → 2) galactose residue^25,47–49^. The reaction of xyloglucan yielded over 20 structurally unique oligosaccharides as determined by nano-HPLC-chip/Q-TOF MS. The most abundant oligosaccharides are shown in Fig. 2a, while the complete mass/rt library and annotated profiles are provided in the Supplementary Data 1, 2, respectively.

The degrees of polymerization (DPs) were obtained from the accurate masses, while structural information was obtained from tandem MS. Isomers, compounds with the same DP but different structures, were present and were structurally elucidated. For example, four isomers with the compositions three hexoses and two pentoses (Hex_3_Pnt_2_, *m*/*z* 771.27) were observed at 16.0 (compound I), 18.9 (II), 21.0 (III), and 31.2 (IV) min, respectively (Fig. 2b). The corresponding tandem MS (MS/MS) spectra were obtained and annotated (Fig. 2c). The structure of xyloglucan is generally known and can be used to obtain precise structural information of the oligosaccharides. Based on these analyses, the inset structures for the isomers were deduced and were consistent with the known structures of xyloglucan.

**Fig. 3:**
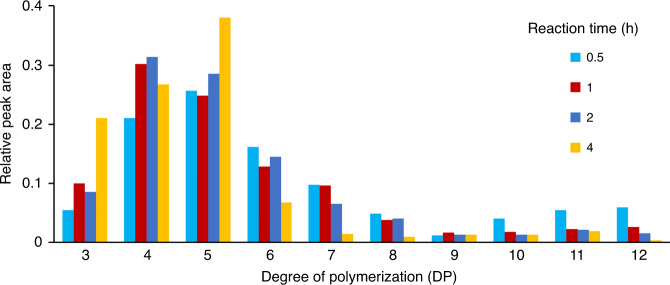
Reaction time and oligosaccharide size. Relative peak area of different size oligosaccharides when reacted from 0.5–4 h.

There was some control on the size distribution of the oligosaccharide products by varying the time of the reaction. At the optimized conditions, the products favored a DP 3–14. However, by increasing the reaction time, smaller DPs were biased. This effect was illustrated with amylose, a simple homopolymer, to provide a clear illustration of the shift in the degree of polymerization (DP) with reaction time. The shortest reaction time, 0.5 h, yielded products with DP 3–14, while at 2 h only those corresponding to DP 3–8 were observed, and at 4 h only those corresponding to DP 3–6 were observed (Fig. 3). While the reaction time did give variability in the DP, other factors such as choice of metal and peroxide concentration may allow the reaction conditions to be further optimized to give oligosaccharide reads of a desired length.

**Fig. 4:**
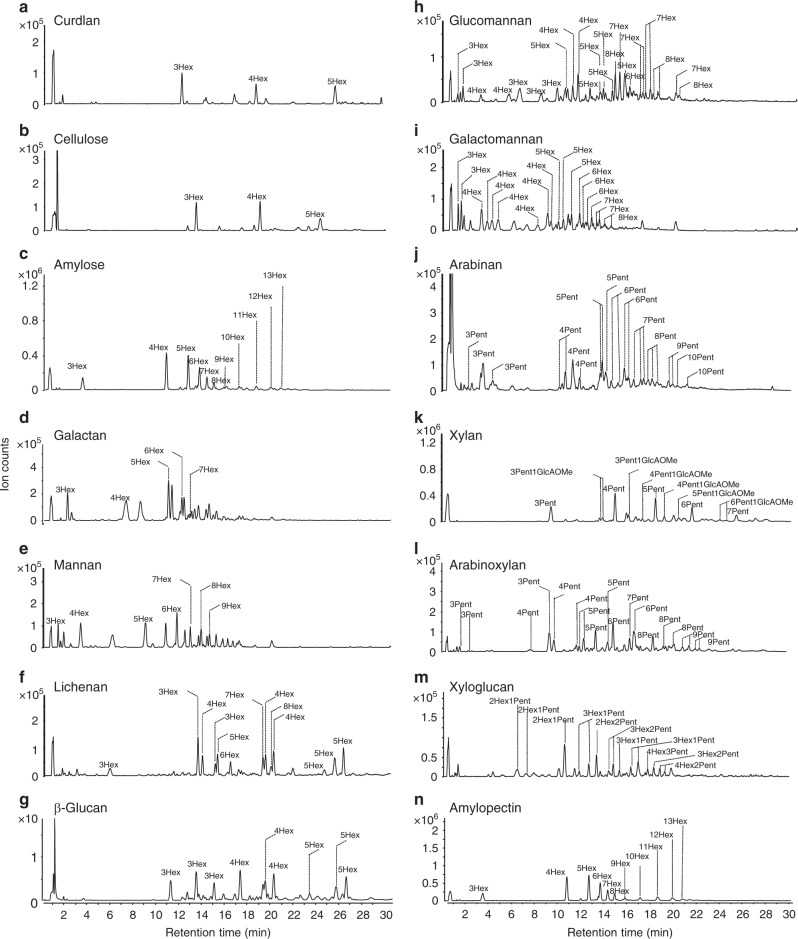
Construction of an oligosaccharide reference library. **a** Annotated base peak chromatograms of oligosaccharides derived from curdlan, **b** cellulose, **c** amylose, **d** galactan, **e** mannan, **f** lichenan, **g** β-glucan, **h** glucomannan, **i** galactomannan, **j** arabinan, **k** xylan, **l** arabinoxylan, **m** xyloglucan, and **n** amylopectin.

To determine the general utility of the approach, other polysaccharide standards were subjected to FITDOG and yielded representative oligosaccharides (Fig. 4). The polysaccharide standards included amylose, amylopectin, cellulose, curdlan, lichenan, β-glucan, arabinan, xylan, arabinoxylan, mannan, galactomannan, glucomannan, galactan, and xyloglucan. These represent a large proportion of known polysaccharides and were selected because they were composed of many monosaccharide combinations, linkage types, branching patterns, while some contained monosaccharide modifications. Annotated chromatograms for each polysaccharide after FITDOG are shown in Fig. 4a–n. Prominent peaks were labeled with the DP and an abbreviation of their monosaccharide components (Hexose-Hex, Pentose-Pnt, Hexuronic Acid/Hxa, 4-*O*-methylated glucuronic acid – GlcAOMe) (Fig. 4). A more comprehensive table of the oligosaccharides produced were listed in the Supplementary Data 3.

A single procedure worked for all of the polysaccharides we tested. The homopolysaccharides, a group of polymers that are composed of a single monosaccharide and glycosidic linkage, yielded linear oligosaccharides with a single representation for each DP (no isomers). For example, curdlan, a linear β(1 → 3) linked glucose polymer yielded oligosaccharides with compositions corresponding to Hex_3_ (RT 12.56 min), Hex_4_ (19.28 min), and Hex_5_ (26.44 min) (Fig. 4a). Other homopolymers included cellulose with β(1 → 4)glucose (Fig. 4b), amylose α(1 → 4) glucose (4c), galactan β(1 → 4)galactose (4d), and mannan β(1 → 4)mannose (4e). It was noted that impurities remained in the polysaccharide from the manufacturer process yielding peaks corresponding to other polysaccharides.

More complicated structures including heteropolysaccharides, which contained more than one monosaccharide or glycosidic linkage, also yielded structurally representative oligosaccharides. The structural heterogeneity contributed to the production of several oligosaccharide isomers for each DP. Among this group were β-glucan and lichenan, both of which have similar structures composed of β(1 → 3) and β(1 → 4) linked glucose residues, but in differing ratios. Thus, the composition corresponding to Hex_6_ was observed to generate eight isomers from lichenan and four from β-glucan (Fig. 4f, g). Moreover, the structural similarities in monosaccharide and linkage composition between lichenan and β-glucan yielded many similar oligosaccharides from both polysaccharides. For example, the Hex_3_ (13.73 min), Hex_4_ (19.46, 19.70, and 20.41 min), Hex_5_ (24.92, 25.79 min), and Hex_6_ (30.90 min) were identical and shared by the two polysaccharides (Fig. 4f, g). Glucomannan, another linear heteropolymer composed of β(1 → 4) glucose and mannose residues, also yielded several isomers for each DP (Fig. 4h).

Branched polysaccharides that contain multiple linkage types and multiple monosaccharides yielded the most isomers per DP. Galactomannan is a branched heteropolysaccharides containing a β(1 → 4) linked mannose backbone and α(1–6) linked terminal galactose branches. With FITDOG, the oligosaccharides resembled those from mannooligosaccharides, derived from the mannose backbone, and galactomannooligosaccharides derived from the branching regions (Fig. 4i). Arabinan, xylan, arabinoxylan, and xyloglucan, all are branched polysaccharides with mixed monomeric units, and were also dissociated to similarly generate many isomeric species (Fig. 4j–m). The dissociation of these diverse structures further demonstrated the method’s reactivity towards pentose- and hexose-containing polysaccharides and highly complicated structures. Unexpectedly, amylopectin, another branched heteropolysaccharide containing an α(1 → 4) linked glucose backbone with α(1 → 4,6) bisecting glucose, produced only one structure per DP. The presence of a single isomer from amylopectin may result from a cleavage preference for α(1 → 6) branching points, thereby producing linear oligosaccharide(Fig. 4n)^50^.

Polysaccharides containing modified monosaccharide residues were also examined to determine whether FITDOG removed saccharide modifications. Xylan contained β(1 → 4)-linked xylose polysaccharide with terminal 4-O-methyl glucuronic acid (GlcAOMe) residues. Under FITDOG conditions, several oligosaccharides were generated with unique compositions including Pnt_3_ GlcAOMe (4 isomers), Pent_4_ GlcAOMe (e isomers), Pent_5_ GlcAOMe (4 isomers), Pent_6_ GlcAOMe (3 isomers), and Pent_7_ GlcAOMe (2 isomers). The presence of these oligosaccharides indicated that O-methylation on the terminal glucuronic acid was preserved during the FITDOG treatment (Fig. 4k). Other modifications are known for carbohydrates including sulfation, ferulic acid, phosphorylation. How these modifications will be impacted by this method is currently under investigation.

### Identification of polysaccharides by fingerprinting

**Fig. 5:**
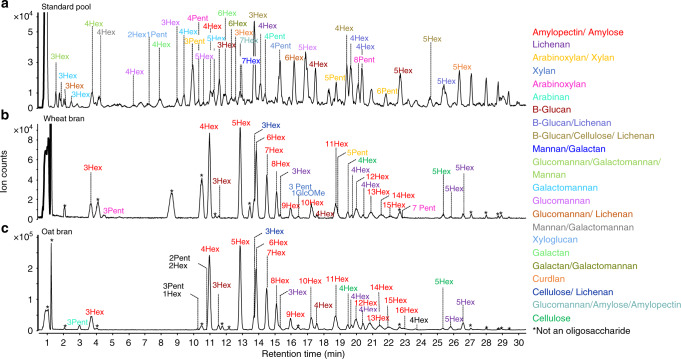
Oligosaccharide fingerprinting. **a** Annotated base peak chromatogram of pooled oligosaccharides derived from the depolymerization of the polysaccharide standards. Annotated base peak chromatograms of characterized oligosaccharides derived from (**b**) wheat bran and (**c**) oat bran.

With the unique oligosaccharide compositions representing each parent polysaccharide, we determined whether the oligosaccharides could be used as diagnostic markers for polysaccharide identification in complicated mixtures such as those found in carbohydrate mixtures. This approach is analogous in proteomics to peptide fingerprinting, which is a robust method for identifying proteins based upon the presence of diagnostic peptides. The oligosaccharide products of polysaccharides were compiled as unique identifiers for the respective parent, which created an oligosaccharide reference library with nearly 400 unique oligosaccharides (Fig. 5a, Supplementary Data 3).

Wheat and oat bran were selected to validate the oligosaccharide fingerprinting method as they were known to contain large amounts of non-starch polysaccharides including arabinoxylans^51^, mixed linkage β-glucans^52^, and cellulose^53^. Oat and wheat bran each produced over 50 distinct oligosaccharides with most matching entries in the reference library (Fig. 5b, c). A pool containing all the oligosaccharides was run alongside unknown samples for retention time alignment and source-polymer identification (Fig. 5a). The full list of matched peaks is presented in the Supplementary Data 4. For identification, we used the number of oligosaccharides produced in the individual standards to determine the fraction of the polysaccharide products that are observed in the mixture. This number varied with each standard polysaccharide. For cellulose, only four oligosaccharides were observed in our chromatographic window, while for amylopectin over 20 oligosaccharides were observed, and for arabinoxylan over 40 oligosaccharides were observed. We then used the number of oligosaccharides found in the mixture represented as a fraction of those found in the pure standard as a percent coverage. The coverage is expected to be higher if the oligosaccharide count in the sample is closer to the number of oligosaccharides produced in the standard.

Based on the FITDOG analyses, we found that wheat bran contained amylose/amylopectin (100% coverage), cellulose (75%), β-glucan (34%), and lichenan (24%). Oat bran was composed of amylose/amylopectin (100% coverage), cellulose (75%), β-glucan (38%), and lichenan (24%). Wheat bran also contained arabinoxylan (20%) and xylan (32%). Interestingly, oat bran contained several unmatched pentose oligomers and mixed hexose and pentose containing oligomers demonstrating the presence of a polysaccharide that is yet to be identified by our reference library, which is binned for future identification. Overall, the results matched the general expected polysaccharide composition of both brans^51–53^.

The ability to identify polysaccharides in complicated matrices will yield the biological functions of polysaccharides. In particular, the role of fiber in food is not well understood due to the lack of analytical tools to characterize them. As an illustration of the utility of the method, we probed the fate of complimentary food polysaccharides in an infant gut by monitoring the changes in compositions between the food product and infant feces after consumption. The infants were part of a cohort comprised of exclusively breastfed infants who were fed complementary food in a crossover feeding trial. An infant was fed pear as their first solid food along with continued breastfeeding. Upon completion of the 7-day feeding period, a fecal sample was taken and analyzed for comparison with the native food. This study would provide the fate of dietary carbohydrates as they interact with the infant gut microbiome.

**Fig. 6:**
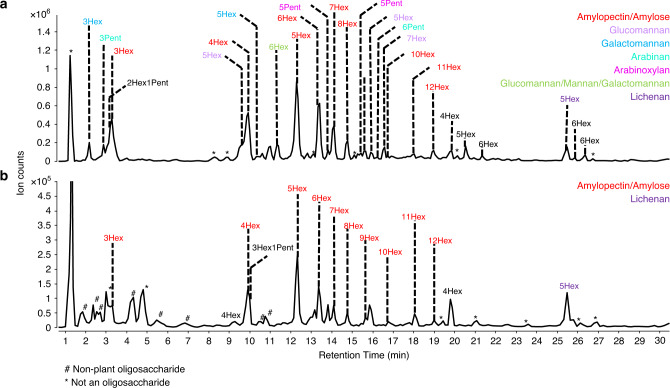
Analysis of in vivo microbe–carbohydrate interactions. Annotated base peak chromatograms of oligosaccharides derived from FITDOG treatment of the ethanol precipitate fraction from (**a**) pear and (**b**) feces.

Polysaccharides that made up the pear product were examined and found to be composed of, in decreasing order, amylopectin/amylose, glucomannan, arabinan, arabinoxylan, galactomannan, mannan, and lichenan (Fig. 6a). Analysis of the feces yielded primarily amylopectin, amylose, and lichenan. (Fig. 6b). These results suggested that amylose, amylopectin, and lichenan were not completely digested while glucomannan, galactomannan, arabinan, arabinoxylan, and mannan appeared to be degraded by host and microbial enzymes.

### De novo structural characterization of oligosaccharides

**Fig. 7:**
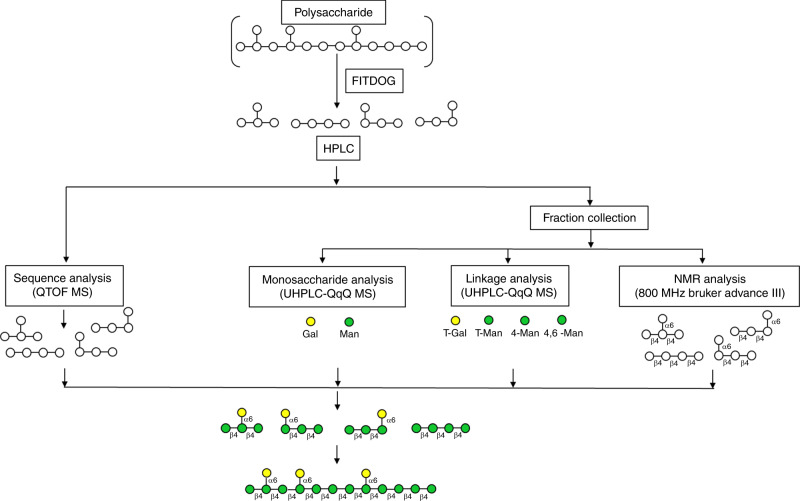
Schematic workflow of the de novo analysis of oligosaccharides derived from polysaccharide depolymerization. Galactomannan is exemplified. Green circles represent mannose and yellow circles represent galactose.

Oligosaccharides produced by FITDOG were further structurally elucidated to provide, de novo, the parent polysaccharide. The workflow is shown in Fig. 7. For the analysis of the constitutive oligosaccharides, the products were separated into pools of smaller numbers of compounds many containing single unique structures. The LC eluant was split between the QTOF for MS and MS/MS and a 96-well collection plate. The QTOF MS and MS/MS mass spectra provided some structural information, in terms of monosaccharide compositions and the degree of oligosaccharide polymerization, of the compounds collected at unique retention times. However, it could not identify monosaccharide constituents, linkages, or the anomeric character of the linkage (alpha versus beta). The nearly 200 collected fractions were further analyzed by rapid throughput monosaccharide and linkage analysis to provide nearly complete structures of each oligosaccharide component^54–57^.

**Fig. 8:**
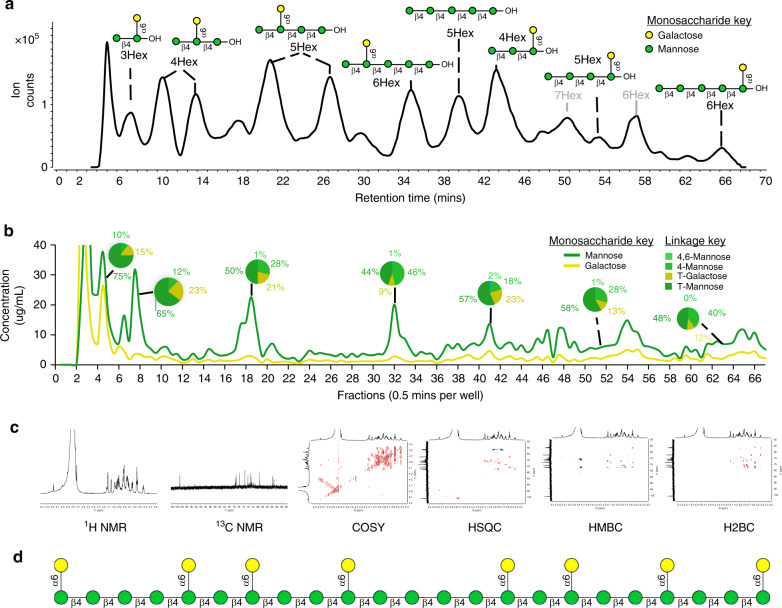
De novo characterization of polysaccharides. **a** Annotated total compound chromatograms with completely elucidated FITDOG oligosaccharides from galactomannan. **b** Constructed chromatogram representing the monosaccharide concentration galactose and mannose in each fraction and the relative glycosidic linkage composition of fractions shown in pie charts. **c** NMR analysis of FITDOG oligosaccharides. **d** A representation of the elucidated galactomannan structure by integrating oligosaccharide, monosaccharide, glycosidic linkage, and NMR analysis.

The galactomannan polysaccharide was used to illustrate the capabilities of these workflows. The LC–MS chromatogram yielded primarily hexose polymers with unknown monosaccharide compositions (combinations of galactose and mannose), linkage, or branching (Fig. 8a). Fractions of the LC–MS were collected in 30 s increments leading to a total of 192 fractions. Each fraction was analyzed for monosaccharide compositions using a recently described rapid throughput automated workflow (Fig. 8b)^54,57^. The linkage information was similarly obtained using a separate recently reported workflow for comprehensive linkage analysis^55,56^. The monosaccharide analysis employed acid hydrolysis to produce monosaccharides that were subsequently labeled with 3-methyl-1-phenyl-2-pyrazoline-5-one (PMP). UHPLC-QqQ MS analysis required 10 min of run time and measured the absolute abundances of 14 monosaccharides. The glycosidic linkages were analyzed with permethylation followed by acid hydrolysis and PMP labeling. The run time was 15 min and was capable of identifying over 100 distinct glycosidic linkages. The monosaccharide and linkage analysis methods were chosen for their speed, which allowed 96-well plates to be quickly analyzed, and their sensitivity, which allowed multiple analyses to be performed on single HPLC fractions. When the three platforms were integrated, they yielded structural information that could describe oligosaccharides with monosaccharide compositions and glycosidic linkage information. To obtain the final structural feature, the anomeric character of the linkages, the most abundant components were selected for NMR analysis using ^1^H, ^13^C NMR and a combination of techniques including COSY, HSQC, HMBC, and H2BC (Fig. 8c, Supplementary Data 5). The MS-obtained linkages and monosaccharide compositions greatly facilitated the NMR interpretation by limiting the resolution needed to determine the exact structures. This multi-platform approach yielded absolute oligosaccharide structures, which were used to recapitulate the parent polymeric structure (Fig. 8d).

## Discussion

The life sciences have built a remarkable understanding of the functions of cells and organisms driven by innovations in mapping the structures of life’s biopolymers. As the most abundant biopolymer in nature, the lack of structural methods for polysaccharides has been glaring. Carbohydrate polymers are central to many biological processes. Cell-cell recognition is based on complicated glycan structures^58,59^; protection is provided by complex carbohydrates^60,61^ and importantly, fuel and carbon flows through ecosystems primarily as complex carbohydrates^62,63^. The importance of glycan structures to interkingdom biology was vividly revealed by research on human milk that demonstrated that human mothers produce complicated, structure specific, oligosaccharides not to feed their infants but to feed specific bacteria within their infants^11,32–34,64^. While the prebiotic effects of oligosaccharides are more widely understood, their antimicrobial and antibiofilm activities are currently under intense investigations^65–67^. The challenge of understanding and managing the flows of carbohydrates across all of biology is solving the complexity of carbohydrate structures also across biology. Yet this complexity is far greater than the other biopolymer classes nucleotides and proteins combined. Elucidating carbohydrate structures has been daunting, but a major impediment—the structure agnostic production of oligosaccharides—has now been solved.

The features of FITDOG make it suitable to measure polysaccharides with accuracy, sensitivity, and throughput. Central to the FITDOG analytical strategy is the ability to produce distinct oligosaccharides that are unique to their respective parent polysaccharides. The lack of mono- and disaccharides demonstrates that the catalysis is equivalent to an endo-, rather than an exoglycosidase enzyme. The results further indicate that the cleavages are not random thereby producing distinct oligosaccharides that are consistent with the cleavages of specific glycosidic bonds. The chemical basis underlying the glycosidic specificity of the FITDOG process, in effect favoring the cleavage of certain bonds, is not yet known. The regioselectivity of iron coordination within the polysaccharide chains likely guides the reaction. It has been suggested that the Fe^3+^ catalyzes the formation of the hydroperoxyl radical species, the site of chelation guides oxidation toward nearby hydroxyl groups that render nearby glycosidic bonds more prone to base-induced beta-elimination^68^. However, this mechanism is not entirely consistent with our observations under the specified conditions, as we do not observe the change in mass associated with the dehydration and formation of the double bond. Determining the true mechanism of this reaction is a topic of ongoing research.

There are side reactions—some that can be suppressed and others that also have further utility. Excessive oxidation can occur including the formation of carboxylic acids in the nascent oligosaccharides. A singular feature of polysaccharide-based Fenton chemistry is that these side reactions can be minimized through properly selected experimental conditions that include the reaction times and the relative concentrations of Fe^3+^ and hydrogen peroxide. For this report, the conditions were optimized, and those presented here yielded the largest number of oligosaccharides while minimizing over oxidation for each polysaccharide tested. Factors that can lead to overoxidation included higher reaction temperatures and high concentrations of hydrogen peroxide. Changing the reaction time can increase or decrease the DP of the oligosaccharide products. Increasing the DP yields greater information regarding the placement of pendant chains from the main polymer chain.

The oligosaccharides produced by FITDOG can be used to identify known polysaccharides structures, which is analogous to peptide fingerprinting in proteomic analysis. This capability will simplify the analysis of complicated polysaccharide mixtures such as those found in food. As different dietary polysaccharides are known to affect the microbiota distinctly, this capability could significantly advance prebiotic/probiotic research.

The availability of a universal chemical approach for polysaccharide dissociation allows modern separation and mass spectrometry methods to be applied to the analysis of complex polysaccharides. We show that oligosaccharide fingerprinting is suitable for identifying known polysaccharides, and the reaction is readily scalable and can be expanded to more polysaccharides as new structures become available. Furthermore, the polysaccharide examples that were illustrated belong to several kingdoms including plants, bacteria, fungi, and algae. The method can also be used to elucidate the structures of new and uncharacterized polysaccharides. We showed that oligosaccharides produced by this method are characterizable by tandem MS methods where a more in-depth multi-platform strategy can systematically provide exact oligomer structure. While we have compared the current strategy to peptide fingerprinting, the analogy ends here. Structural analyses of oligosaccharides are significantly more difficult than that of peptides. Therefore, sequencing by tandem MS, a common tool for peptides, yields highly limited information with oligosaccharides, especially of those with limited monomeric mass diversity. However, we show that de novo structural elucidation of oligosaccharides, which previously involved resource extensive processes, can now be performed rapidly. With the structures elucidated, the oligosaccharides can be reconstructed to determine the overall structure of the parent polysaccharide (Fig. 8d). This method can also be applied for elucidating the structures of new polysaccharides in a much more rapid manner than is currently available.

Finally, this method may find additional utility beyond analysis. When scaled, this method provides the creation of new and previously unattainable oligosaccharides that can be further probed for bioactivity. We look forward to these results as we predict that the method will substantially advance carbohydrate research.

## Methods

### Experimental materials

Sodium acetate, hydrogen peroxide (H_2_O_2_), sodium hydroxide (NaOH), sodium borohydride (NaBH_4_), iron(III) sulfate pentahydrate (Fe_2_(SO_4_)_3_), and glacial acetic acid were purchased from Sigma-Aldrich (St. Louis, MO). Amylopectin was obtained from Carbosynth (Compton, UK). Amylose, xyloglucan, arabinoxylan, xylan, glucomannan, galactomannan, mannan, curdlan, galactan, β-glucan, arabinan, and lichenan were purchased from Megazyme (Bray, Ireland). Microcrystalline cellulose was purchased from ACROS Organics. Formic Acid (FA) was purchased from Fisher Scientific (Belgium, UK). Acetonitrile (HPLC grade) was purchased from Honeywell (Muskegon, MI). Nano-pure water was used for all experiments.

### FITDOG generation of oligosaccharides

A solution was prepared containing 95% (v/v) 40 mM sodium acetate buffer adjusted to pH 5 with glacial acetic acid, 5% (v/v) hydrogen peroxide (30% v/v), and 65 nM iron(III) sulfate. This mixture was vortexed and added to dry polysaccharide standards to make a final solution of 1 mg/ml. The reaction was incubated at 100 °C for 1 h. The reaction was quenched by adding half of the reaction volume of cold 2 M NaOH. Glacial acetic acid was added for neutralization.

Oligosaccharides were reduced by incubation with 1 M NaBH_4_ for 1 h at 65 °C. Oligosaccharides were isolated using nonporous graphitized carbon cartridges. Cartridges were washed with 80% acetonitrile and 0.1% (v/v) TFA in water. The oligosaccharides were loaded and washed with five column volumes of water. The oligosaccharides were eluted with 40% acetonitrile with 0.05% (v/v) TFA. Samples were completely dried by evaporative centrifugation and stored at −20 °C until analysis.

### Sample preparation for fecal fingerprinting

To separate the endogenous oligosaccharides from the polysaccharides, pear and feces samples underwent 80% ethanol precipitation overnight at −80 °C. Samples were centrifuged at 845 × *g* for 20 min to pellet the polysaccharide fraction and partition it from the endogenous oligosaccharides in the supernatant. The pelleted fraction underwent FITDOG treatment to generate representative oligosaccharides. Oligosaccharides were then reduced and purified using the protocol described in the section above.

### Mass spectrometry analysis

For analysis by MALDI-MS, 1 µl was plated directly onto a stainless steel MALDI plate. To this, 0.3 µl of 0.01 M NaCl and 0.7 µl of 25 mg/ml 2, 5-dihydroxybenzoic acid was added and mixed within the pipet tip. The samples were dried under vacuum and analyzed on a Bruker UltraFlextreme MALDI-tandem time-of-flight (MALDI-TOF/TOF) instrument. The instrument was operated in positive mode and 95% of max laser power.

Samples were reconstituted in nano-pure water before analysis by nano-HPLC-chip/Q-TOF MS. The system included two pumps, a capillary pump for sample loading and a nano pump for analytical separation. In this system, an Agilent 1200 series HPLC is coupled to an Agilent 6520 Q-TOF mass spectrometer through a chip cube interface. The chip contained a 40 nl enrichment column, and a 75 µm × 43 mm analytical column; both columns had PGC as the stationary phase. Sample loading was done with 3% (v/v) acetonitrile/water + 0.1% formic acid at a flow rate of 4 µl/min. Chromatographic separation was performed with a binary gradient of solvent A: (3% (v/v) acetonitrile/water + 0.1% formic acid) and solvent B: (90% acetonitrile/water + 0.1% formic acid) with a flow rate of 0.4 µl/min. The gradient was run for 60 min, 1–5% B, 0–2 min; 5–30%, 2–33 min; 30–99%, 33–38 min; 99–99%, 38–48 min; 99–1%, 48–50 min; 1–1%, 50–60 min.

Data were collected in the positive mode and calibrated with internal calibrant ions ranging from *m*/*z* 118.086 to 2721.895. Drying gas was set to 325 °C and with a flow rate of 5 l/min. The fragment, skimmer, and Octapole 1 RF voltages were set to 175, 60, and 750 V, respectively. Fragmentation was performed at a rate of 0.63 spectra/s. The collision energy was based upon the compound mass and expressed by the linear function (collision energy = 1.8 × (*m*/*z*) − 2.4).

Samples were reconstituted in nano-pure water before HPLC Q-TOF MS analysis. The analytical separation was carried out using an Agilent 1260 Infinity II HPLC coupled to an Agilent 6530 Accurate-Mass Q-TOF MS. Chromatographic separation was performed on a 150 mm × 1 mm Hypercarb column from Thermo Scientific with a 5 µm particle size. A binary gradient was employed which consisted of solvent A: (3% (v/v) acetonitrile/water + 0.1% formic acid) and solvent B: (90% acetonitrile/water + 0.1% formic acid). A 45 min gradient with a flow rate of 0.150 mL/min was used for chromatographic separation: 3–25% B, 0–15 min; 25–25% B, 15–18 min; 25–99% B, 18–30 min; 99–99% B, 30–32 min; 99–3% B, 32–34 min; 3–3% B, 34–45 min. Samples were run in the positive mode. Internal calibrant ions ranged from *m*/*z* 121.051 to 2421.914. Drying gas temperature and flow rate were set to 150 °C and 11 l/min, respectively. Operation voltages for the fragment, skimmer, and octupole 1 RF were 175, 60, and 750 V, respectively. The acquisition rate was set to 0.63 spectra/s. When fragmentation was used, the linear function, collision energy = 1.45 × (*m*/*z*) − 3.5, was employed.

### Infant fecal samples

The fecal samples were collected from a healthy, term 6-month-old infant who completed the UC Davis Infant Microbiome Nutrition and Development (Infant MiND) Study. The infant was exclusively breastfed at enrollment. The infant was assigned to consume pear (Earth’s Best Stage 1) concurrently with breast milk for 7 days. After 7 days, parents were instructed to scrape the soiled diaper with sterile utensils, to place the fecal samples into sterile tubes, and to seal and store the samples in their kitchen freezers. The fecal samples were transported back to University of California Davis campus on dry ice and stored in −80 °C before being analyzed. The University of California Davis Institutional Review Board approved all aspects of this study and written informed consent was obtained from the participants (Protocol ID: 919505). This study was registered on clinicaltrials.gov (NCT01817127).

### Fractionation of oligosaccharides

Fractionation and detection of oligosaccharides was performed on an Agilent 1260 Infinity II series HPLC coupled to an Agilent 6530 Q-TOF mass spectrometer and a Teledyne Isco Foxy 200 fraction collector. Oligosaccharides were first separated on a 150 × 4.6 mm Hypercarb column from Thermo Scientific with a 5 µm particle size. A binary gradient was employed and consisted of solvent A: (3% (v/v) acetonitrile/water + 0.1% formic acid) and solvent B: (90% acetonitrile/water + 0.1% formic acid). A 90 min gradient with a flow rate of 1 ml/min was used for chromatographic separation: 5–12% B, 0–90 min; 12–99% B, 90–90.01 min; 99–99% B, 90.01–110 min; 99–5% B, 110–110.01 min; 5–5% B, 110.01–120 min. Post column, a 90:10 flow splitter partitioned the larger stream to the fraction collector and the smaller to the Q-TOF mass spectrometer. Data from the QTOF was collected in the positive mode and calibrated with internal calibrant ions ranging from *m*/*z* 118.086–2721.895. Drying gas was set to 150 °C and with a flow rate of 11 l/min. The fragment, skimmer, and Octupole 1 RF voltages were set to 75, 60, and 750 V, respectively. Fragmentation was performed at a rate of 1 spectra/s. The collision energy was based upon the compound mass and expressed by the linear function (collision energy = 1.3 × (*m*/*z*) − 3.5). Fractions were collected on 96-well plates at a rate of 30 s per fraction. Collected fractions were dried to completion under vacuum centrifugation and reconstituted in 100 μl of nano-pure water. A 10 μl aliquot was transferred to a separate 96-well plate for monosaccharide composition analysis, while the remaining 90 µl underwent glycosidic linkage analysis.

### Monosaccharide composition analysis of fractionated oligosaccharides

Monosaccharide analysis was adapted by Amicucci et al. with the following modifications^54^. Briefly, fractionated oligosaccharides underwent acid hydrolysis with 4 M TFA for 2 h at 100 °C. The samples were dried to completion by vacuum centrifugation. Samples and monosaccharide standards (0.001–100 μg/ml) underwent derivatization with 0.2 M PMP in methanol and 28% NH_4_OH at 70 °C for 30 min. Derivatized products were dried to completion under vacuum centrifugation and reconstituted in nano-pure water. Excess PMP was removed with chloroform extraction. The aqueous layer was analyzed by an Agilent 1290 infinity II UHPLC coupled to an Agilent 6495A QqQ MS employing dynamic multiple reaction monitoring (MRM) mode. An external standard curve was used for absolute quantitation of each monosaccharide in the fractions.

### Linkage analysis of fractionated oligosaccharides

Linkage analysis was adapted from Galermo et al. with the following modifications^55,56^. Briefly, fractionated oligosaccharides and a pool of oligosaccharide standards were reacted with saturated NaOH and iodomethane in DMSO. Residual NaOH and DMSO were removed by extraction with DCM and water. The DCM layer was dried to completion under vacuum centrifugation. Samples were hydrolyzed and derivatized in the same manner as the monosaccharide analysis. Samples did not undergo chloroform extraction and were reconstituted in 70% (v/v) methanol/water. Fractions were analyzed on an Agilent 1290 infinity II UHPLC coupled to an Agilent 6495A QqQ MS ran in MRM mode. A pool of oligosaccharide standards was used to assign the glycosidic linkages present.

### NMR analysis of fractionated oligosaccharides

NMR spectra were recorded at 303 K on a Bruker AVANCE III 800 MHz spectrometer equipped with a 5 mm Bruker CPTCI cryoprobe. Samples were obtained by combining ten collections of fractionated oligosaccharides of the same components verified with HPLC-QTOF MS and MS/MS data. Based on the monosaccharide and linkage information, the most abundant oligosaccharides were selected for NMR analysis. Each of these selected pooled fractions were dried with vacuum centrifugation before being reconstituted in 0.4 mL of D_2_O, and measured using 1D ^1^H (relaxation delay (D1) 2 s; number of scans (NS) 128), ^13^C NMR (D1 1.5 s; NS 6000–15000), and 2D ^1^H–^1^H COSY (D1 1.5 s; NS 8), ^1^H–^13^C HSQC (D1 2 s; NS 4), HMBC (D1 1.5 s; NS 16), and H2BC (D1 1.5 s; NS 16). The spectra were then processed with Bruker TopSpin 3.2 and analyzed with MestReNova. The experimental chemical shifts, along with the required monosaccharide and linkage data, were calculated using the CASPER program^69^, where the oligosaccharide structures, including anomeric characters of linkages, were predicted with ranking scores.

### Reporting summary

Further information on research design is available in the Nature Research Reporting Summary linked to this article.

## Supplementary information

Peer Review File

Description of Additional Supplementary Files

Supplementary Data 1

Supplementary Data 2

Supplementary Data 3

Supplementary Data 4

Supplementary Data 5

Reporting Summary

## Data Availability

The authors declare that the data supporting the findings of the study are available in the paper and in Supplementary Data 1–5. All other data are available from the corresponding author on reasonable request.
