# Supplementary material for: A nonenzymatic method for cleaving polysaccharides to yield oligosaccharides for structural analysis
Source: Nat Commun. 2020 Aug 7;11:3963. doi: 10.1038/s41467-020-17778-1 (PMC7414865; doi:10.1038/s41467-020-17778-1)
Supplement: Supplementary file 2 — Description of Additional Supplementary Files [file 41467_2020_17778_MOESM2_ESM.docx]

SUPPLEMENTARY LEGENDS

**Supplementary Data 1**

Library of structurally elucidated oligosaccharides.

Structures were determined by interpretation of their CID fragmentation spectra. Blue circles represent glucose, yellow circles represent galactose, grey stars represent xylose, and white stars represent an unidentified pentose.

**Supplementary Data 2**

Elucidation of xyloglucan oligosaccharides.

HPLC-MS chromatogram of each m/z, raw tandem mass spectrum, and tandem mass spectrum annotated by in-house software.

**Supplementary Data 3**

Polysaccharide fingerprinting library of polysaccharides.

Compounds are referred to as their acryonym: Hexose/Hex, Pentose/Pent, *O*-methylated Glucuronic Acid/GlcAOMe. Polysaccharides are abbreviated: Curdlan/Curd, Cellulose/Cell, β-Glucan/β-Glc, Lichenan/Lich, Galactan/Gal, Mannan/Man, Glucomannan/GlcMan, Galactomannan/GalMan, Arabinan/Ara, Xylan/Xyl, Arabinoxylan/AraXyl, Amylose/Amy, Amylopectin/AmyP, Xyloglucan/XylGlc. An “**X**” denotes the presence of the corresponding oligosaccharide from the FITDOG depolymerization of the parent polysaccharide.

**Supplementary Data 4**

Polysaccharide fingerprinting of wheat and oat bran.

Compounds are referred to as their acryonym: Hexose/Hex, Pentose/Pent, *O*-methylated Glucuronic Acid/GlcAOMe. Polysaccharides are abbreviated: Curdlan/Curd, Cellulose/Cell, β-Glucan/β-Glc, Lichenan/Lich, Galactan/Gal, Mannan/Man, Glucomannan/GlcMan, Galactomannan/GalMan, Arabinan/Ara, Xylan/Xyl, Arabinoxylan/AraXyl, Amylose/Amy, Amylopectin/AmyP, Xyloglucan/XylGlc. When an oligosaccharide can be from multiple polysaccharides, it is denoted in the column which contains multiple polysaccharide names. When an oligosaccharide can only be found in one polysaccharide, it is denoted in the column with only one polysaccharide name. An “X” denotes the presence of the corresponding oligosaccharide from the FITDOG depolymerization of the parent polysaccharide. An “O” denotes an oligosaccharide that was not found in any polysaccharide standard.

**Supplementary Data 5**

De novo characterization of carbohydrates.

NMR spectra (^1^H, ^13^C, COSY, HSQC, HMBC, H2BC) and MS/MS (HPLC-Q-TOF MS) of collected oligosaccharide fractions of galactomannan FITDOG product. The selected chemical shifts (listed below each spectrum), along with monosaccharide and linkage data, were calculated using the CASPER program, where the oligosaccharide structures were predicted (shown before each set of NMR spectra). Oligosaccharides are referred to as the number of their component hexoses – hex – order.
