# Supplementary material for: A nonenzymatic method for cleaving polysaccharides to yield oligosaccharides for structural analysis
Source: Nat Commun. 2020 Aug 7;11:3963. doi: 10.1038/s41467-020-17778-1 (PMC7414865; doi:10.1038/s41467-020-17778-1)
Supplement: Supplementary file 3 — Supplementary Data 1 [file 41467_2020_17778_MOESM3_ESM.pdf]

Supplementary Data 1

Library of structurally elucidated oligosaccharides employing nano-chip-HPLC/QTOF MS. Structures were determined by interpretation of their CID fragmentation spectra. Blue circles represent glucose, yellow circles represent galactose, grey stars represent xylose, and white stars represent an unidentified pentose.

Number	Composition	Retention Time	Experimental Mass (Da)	Calculated Mass (Da)	Error (Da)	Volume	Structure
**1**	220	14.9	608.2171	608.2164	0.0007	75184112	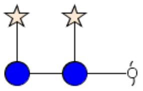
**2**	211a	11.3	638.2275	638.2269	0.0006	17781760	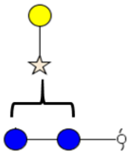
**3**	211b	12.1	638.2275	638.2269	0.0006	52542280	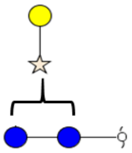
**4**	310a	22.3	638.2268	638.2269	0.0001	21461504	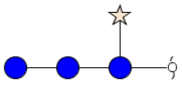
**5**	310b	24.8	638.2268	638.2269	0.0001	11417947	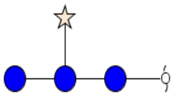
**6**	310c	27.8	638.2273	638.2269	0.0004	22907458	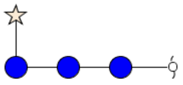

**7**	131a	13.9	740.258	740.2586	0.0006	1002178	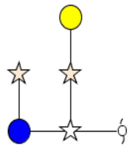
**8**	131b	17.2	740.2585	740.2586	0.0001	5929352	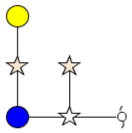
**9**	230a	28.8	740.2583	740.2586	0.0003	2067635	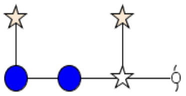
**10**	230b	30.1	740.2583	740.2586	0.0003	1999852	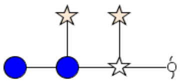
**11**	230c	31.8	740.2581	740.2586	0.0005	2458508	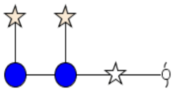
**12**	221a	16	770.2692	770.2692	<0.0001	6940470	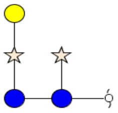
**13**	221b	18.8	770.2694	770.2692	0.0002	9330536	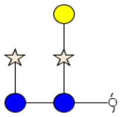

**14**	320a	20.9	770.2694	770.2692	0.0002	50428980	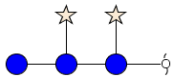
**15**	320b	31.2	770.2692	770.2692	<0.0001	26768034	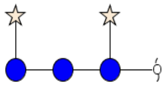
**16**	311a	25.6	800.2795	800.2798	0.0003	8189866	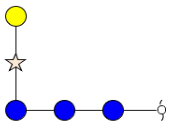
**17**	311b	27.3	800.2793	800.2798	0.0005	4528094	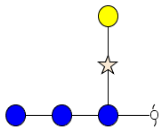
**18**	311c	30.4	800.2802	800.2798	0.0004	16758543	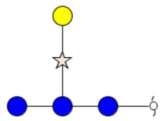
**19**	132	19.7	902.3111	902.3115	0.0004	1975302	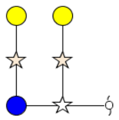
**20**	321	24.4	932.3221	932.322	0.0001	20507106	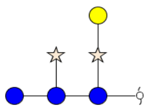
**21**	420	31.7	932.3227	932.322	0.0007	17290708	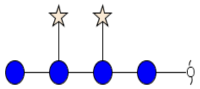
