# Supplementary material for: A nonenzymatic method for cleaving polysaccharides to yield oligosaccharides for structural analysis
Source: Nat Commun. 2020 Aug 7;11:3963. doi: 10.1038/s41467-020-17778-1 (PMC7414865; doi:10.1038/s41467-020-17778-1)
Supplement: Supplementary file 4 — Supplementary Data 2 [file 41467_2020_17778_MOESM4_ESM.pdf]

Supplementary Data 2

HPLC-MS chromatogram of each m/z, raw tandem mass spectrum, and tandem mass spectrum annotated by in-house software.

2Hex2Pent

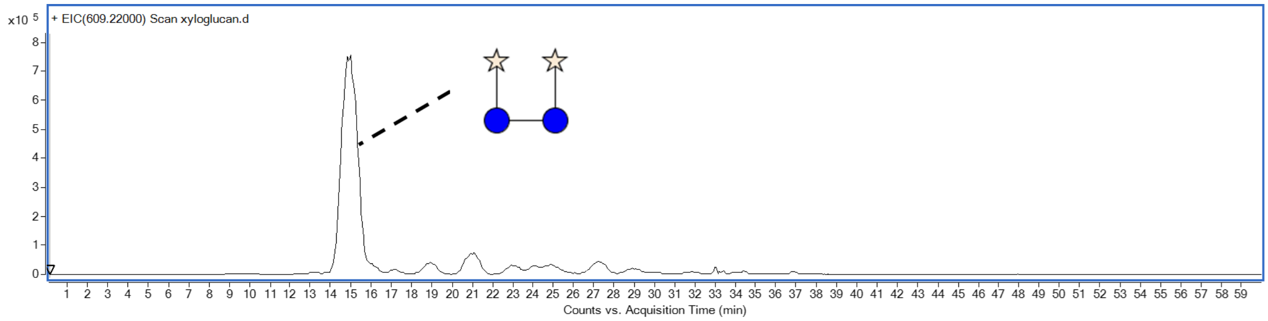

2Hex2Pent

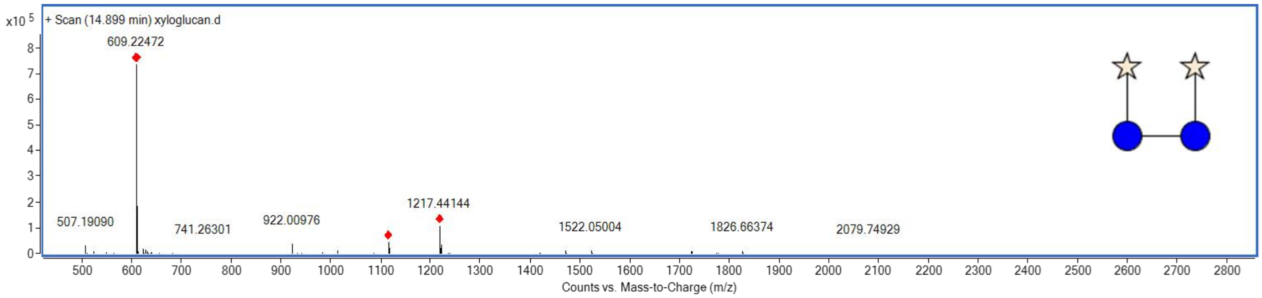

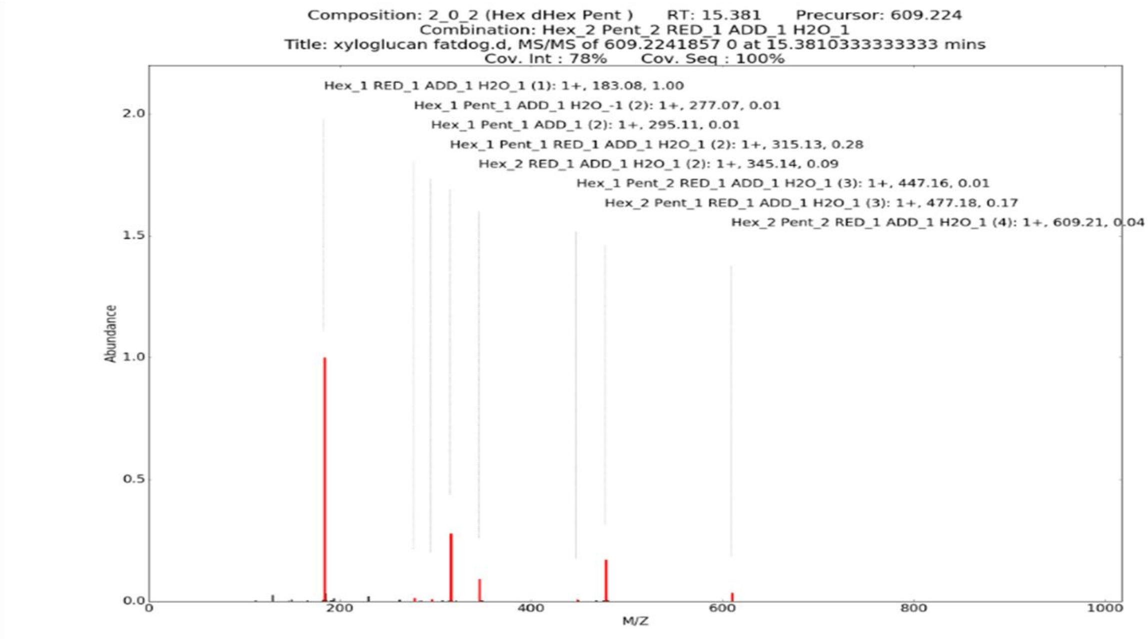

3Hex1Pent

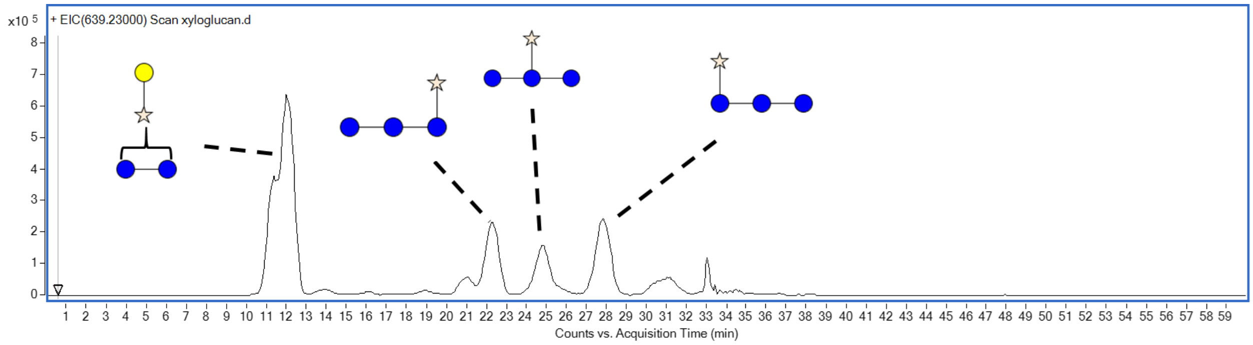

3Hex1Pent (a/b)

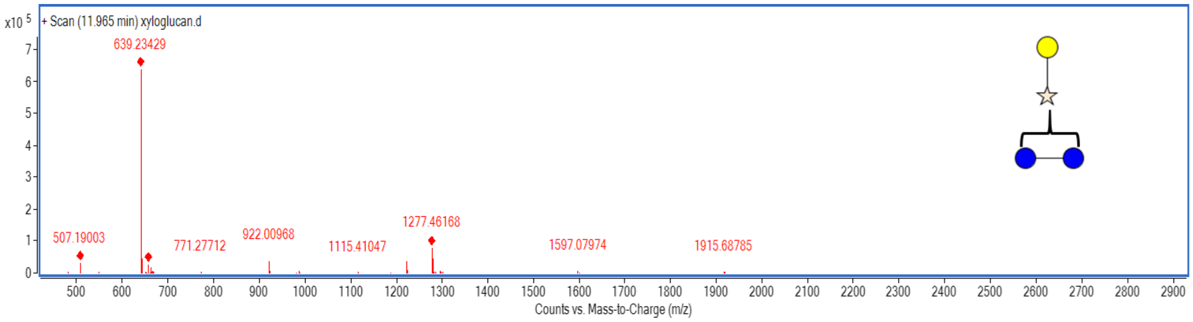
x10 5
+ Scan (11.965 min) xyloglucan.d
639.23429
507.19003
771.27712
922.00968
1115.41047
1277.46168
1597.07974
1915.68785
Counts vs. Mass-to-Charge (m/z)

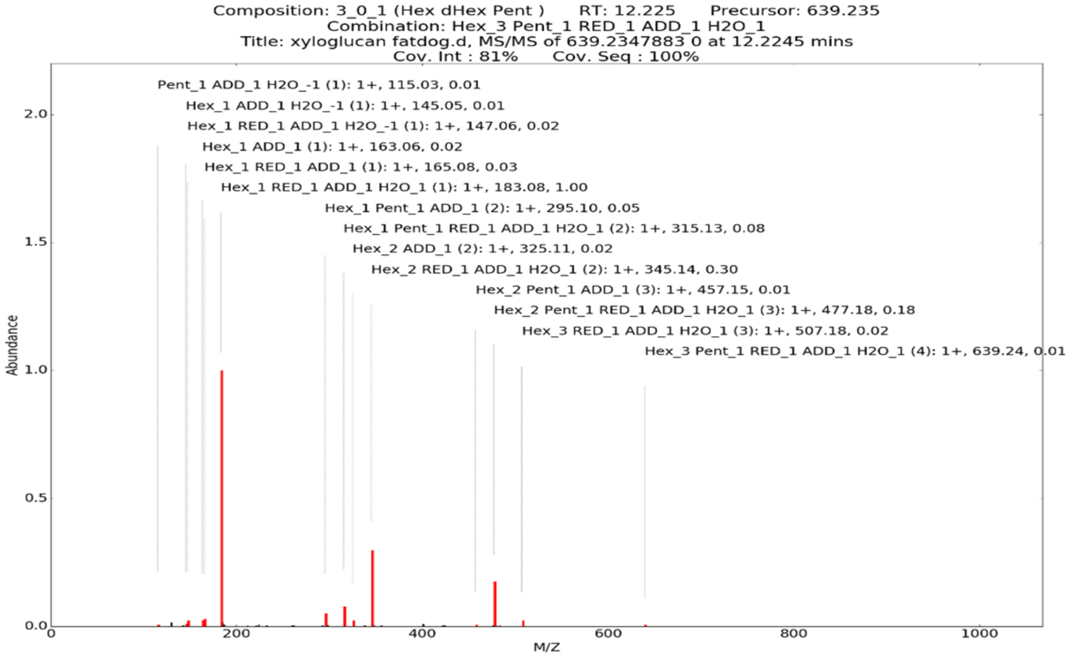
Composition: 3_0_1 (Hex dHex Pent) RT: 12.225 Precursor: 639.235
Combination: Hex_3 Pent_1 RED_1 ADD_1 H2O_1
Title: xyloglucan fatdog.d, MS/MS of 639.2347883 0 at 12.2245 mins
Cov. Int : 81% Cov. Seq : 100%
Pent_1 ADD_1 H2O_-1 (1): 1+, 115.03, 0.01
Hex_1 ADD_1 H2O_-1 (1): 1+, 145.05, 0.01
Hex_1 RED_1 ADD_1 H2O_-1 (1): 1+, 147.06, 0.02
Hex_1 ADD_1 (1): 1+, 163.06, 0.02
Hex_1 RED_1 ADD_1 (1): 1+, 165.08, 0.03
Hex_1 RED_1 ADD_1 H2O_1 (1): 1+, 183.08, 1.00
Hex_1 Pent_1 ADD_1 (2): 1+, 295.10, 0.05
Hex_1 Pent_1 RED_1 ADD_1 H2O_1 (2): 1+, 315.13, 0.08
Hex_2 ADD_1 (2): 1+, 325.11, 0.02
Hex_2 RED_1 ADD_1 H2O_1 (2): 1+, 345.14, 0.30
Hex_2 Pent_1 ADD_1 (3): 1+, 457.15, 0.01
Hex_2 Pent_1 RED_1 ADD_1 H2O_1 (3): 1+, 477.18, 0.18
Hex_3 RED_1 ADD_1 H2O_1 (3): 1+, 507.18, 0.02
Hex_3 Pent_1 RED_1 ADD_1 H2O_1 (4): 1+, 639.24, 0.01
Abundance
M/Z

3Hex1Pent (c)

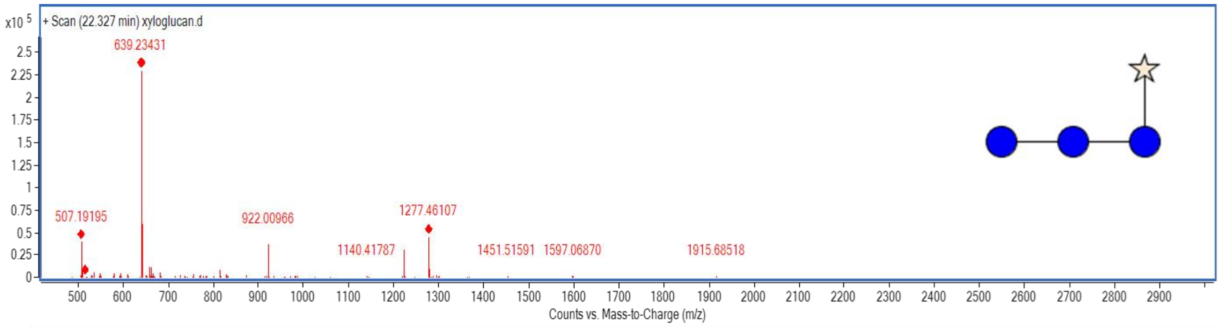
x10 5
+ Scan (22.327 min) xyloglucan.d
639.23431
507.19195
922.00966
1277.46107
1140.41787
1451.51591
1597.06870
1915.68518
Counts vs. Mass-to-Charge (m/z)

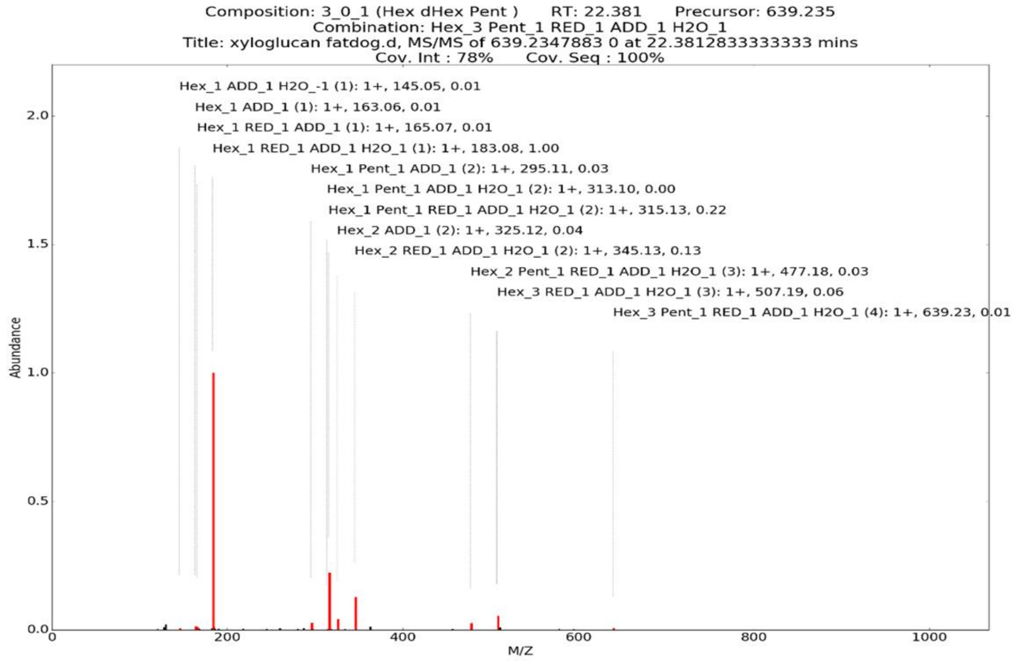
Composition: 3_0_1 (Hex dHex Pent) RT: 22.381 Precursor: 639.235
Combination: Hex_3 Pent_1 RED_1 ADD_1 H2O_1
Title: xyloglucan fatdog.d, MS/MS of 639.2347883 0 at 22.3812833333333 mins
Cov. Int : 78% Cov. Seq : 100%
Hex_1 ADD_1 H2O_-1 (1): 1+, 145.05, 0.01
Hex_1 ADD_1 (1): 1+, 163.06, 0.01
Hex_1 RED_1 ADD_1 (1): 1+, 165.07, 0.01
Hex_1 RED_1 ADD_1 H2O_1 (1): 1+, 183.08, 1.00
Hex_1 Pent_1 ADD_1 (2): 1+, 295.11, 0.03
Hex_1 Pent_1 ADD_1 H2O_1 (2): 1+, 313.10, 0.00
Hex_1 Pent_1 RED_1 ADD_1 H2O_1 (2): 1+, 315.13, 0.22
Hex_2 ADD_1 (2): 1+, 325.12, 0.04
Hex_2 RED_1 ADD_1 H2O_1 (2): 1+, 345.13, 0.13
Hex_2 Pent_1 RED_1 ADD_1 H2O_1 (3): 1+, 477.18, 0.03
Hex_3 RED_1 ADD_1 H2O_1 (3): 1+, 507.19, 0.06
Hex_3 Pent_1 RED_1 ADD_1 H2O_1 (4): 1+, 639.23, 0.01
Abundance
M/Z

3Hex1Pent (d)

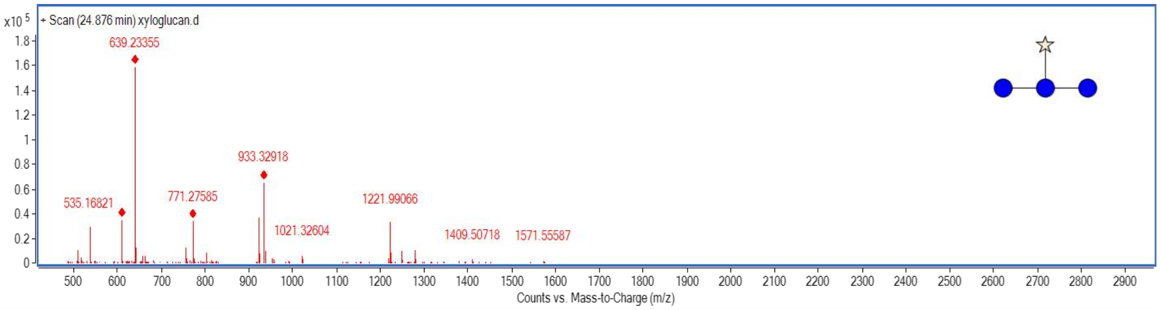
x10 5
+ Scan (24.876 min) xyloglucan.d
639.23355
933.32918
535.16821
771.27585
1221.99066
1021.32604
1409.50718
1571.55587
Counts vs. Mass-to-Charge (m/z)

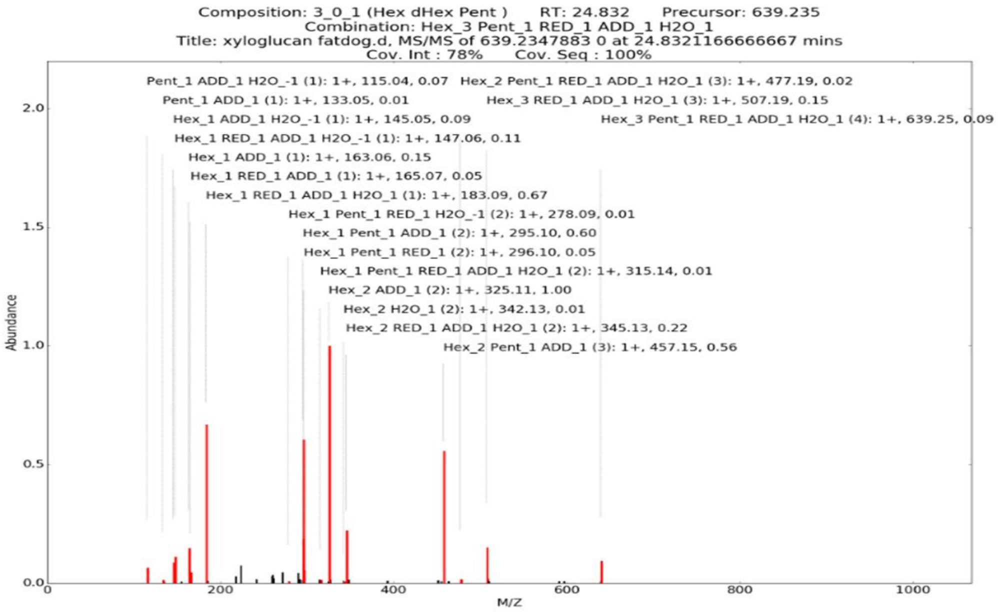
Composition: 3_0_1 (Hex dHex Pent) RT: 24.832 Precursor: 639.235
Combination: Hex_3 Pent_1 RED_1 ADD_1 H2O_1
Title: xyloglucan fatdog.d, MS/MS of 639.2347883 0 at 24.8321166666667 mins
Cov. Int : 78% Cov. Seq : 100%
Pent_1 ADD_1 H2O_-1 (1): 1+, 115.04, 0.07
Hex_2 Pent_1 RED_1 ADD_1 H2O_1 (3): 1+, 477.19, 0.02
Pent_1 ADD_1 (1): 1+, 133.05, 0.01
Hex_3 RED_1 ADD_1 H2O_1 (3): 1+, 507.19, 0.15
Hex_1 ADD_1 H2O_-1 (1): 1+, 145.05, 0.09
Hex_3 Pent_1 RED_1 ADD_1 H2O_1 (4): 1+, 639.25, 0.09
Hex_1 RED_1 ADD_1 H2O_-1 (1): 1+, 147.06, 0.11
Hex_1 ADD_1 (1): 1+, 163.06, 0.15
Hex_1 RED_1 ADD_1 (1): 1+, 165.07, 0.05
Hex_1 RED_1 ADD_1 H2O_1 (1): 1+, 183.09, 0.67
Hex_1 Pent_1 RED_1 H2O_-1 (2): 1+, 278.09, 0.01
Hex_1 Pent_1 ADD_1 (2): 1+, 295.10, 0.60
Hex_1 Pent_1 RED_1 (2): 1+, 296.10, 0.05
Hex_1 Pent_1 RED_1 ADD_1 H2O_1 (2): 1+, 315.14, 0.01
Hex_2 ADD_1 (2): 1+, 325.11, 1.00
Hex_2 H2O_1 (2): 1+, 342.13, 0.01
Hex_2 RED_1 ADD_1 H2O_1 (2): 1+, 345.13, 0.22
Hex_2 Pent_1 ADD_1 (3): 1+, 457.15, 0.56
Abundance
M/Z

3Hex1Pent (e)

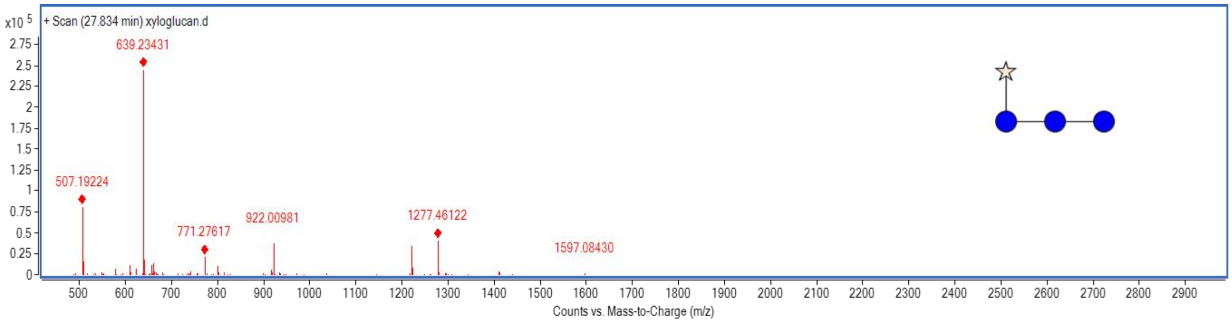
x10 5
+ Scan (27.834 min) xyloglucan.d
639.23431
507.19224
771.27617
922.00981
1277.46122
1597.08430
Counts vs. Mass-to-Charge (m/z)

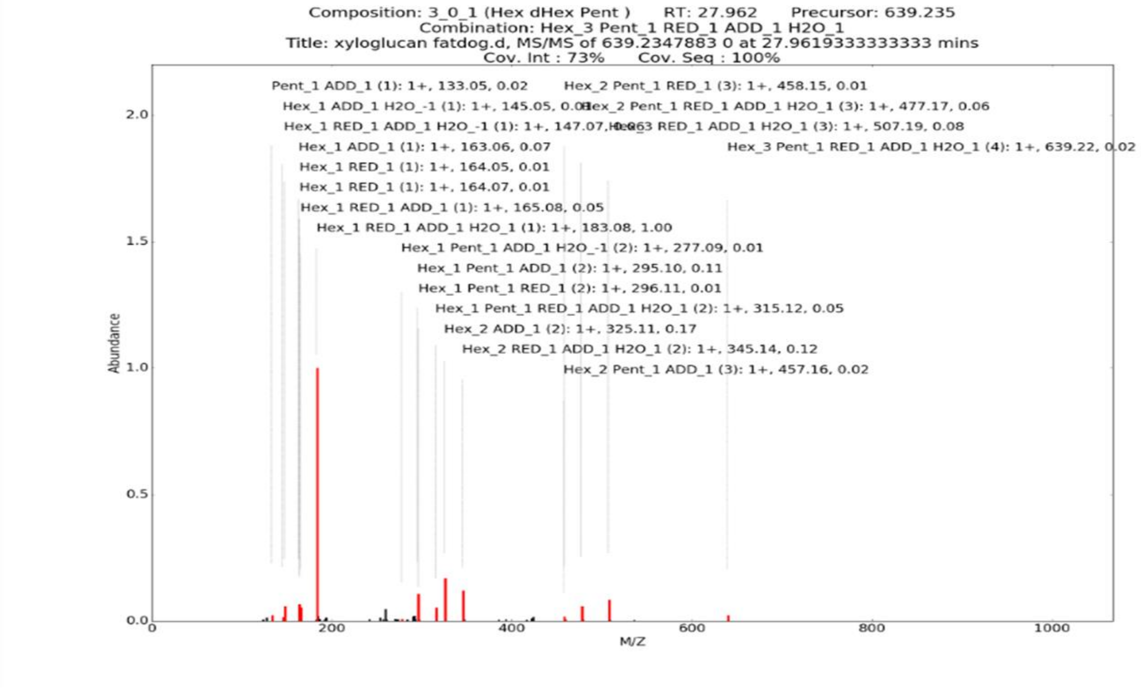
Composition: 3_0_1 (Hex dHex Pent) RT: 27.962 Precursor: 639.235
Combination: Hex_3 Pent_1 RED_1 ADD_1 H2O_1
Title: xyloglucan fatdog.d, MS/MS of 639.2347883 0 at 27.9619333333333 mins
Cov. Int : 73% Cov. Seq : 100%
Pent_1 ADD_1 (1): 1+, 133.05, 0.02
Hex_1 ADD_1 H2O_-1 (1): 1+, 145.05, 0.06
Hex_1 RED_1 ADD_1 H2O_-1 (1): 1+, 147.07, 0.06
Hex_1 ADD_1 (1): 1+, 163.06, 0.07
Hex_1 RED_1 (1): 1+, 164.05, 0.01
Hex_1 RED_1 (1): 1+, 164.07, 0.01
Hex_1 RED_1 ADD_1 (1): 1+, 165.08, 0.05
Hex_1 RED_1 ADD_1 H2O_1 (1): 1+, 183.08, 1.00
Hex_1 Pent_1 ADD_1 H2O_-1 (2): 1+, 277.09, 0.01
Hex_1 Pent_1 ADD_1 (2): 1+, 295.10, 0.11
Hex_1 Pent_1 RED_1 (2): 1+, 296.11, 0.01
Hex_1 Pent_1 RED_1 ADD_1 H2O_1 (2): 1+, 315.12, 0.05
Hex_2 ADD_1 (2): 1+, 325.11, 0.17
Hex_2 RED_1 ADD_1 H2O_1 (2): 1+, 345.14, 0.12
Hex_2 Pent_1 ADD_1 (3): 1+, 457.16, 0.02
Hex_2 Pent_1 RED_1 (3): 1+, 458.15, 0.01
Hex_2 Pent_1 RED_1 ADD_1 H2O_1 (3): 1+, 477.17, 0.06
Hex_3 RED_1 ADD_1 H2O_1 (3): 1+, 507.19, 0.08
Hex_3 Pent_1 RED_1 ADD_1 H2O_1 (4): 1+, 639.22, 0.02
Abundance
M/Z

2Hex3Pent

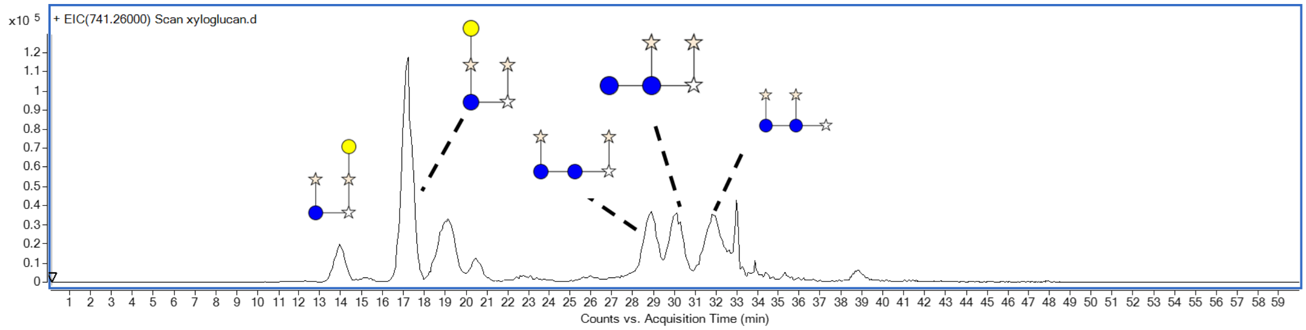

2Hex3Pent (a)

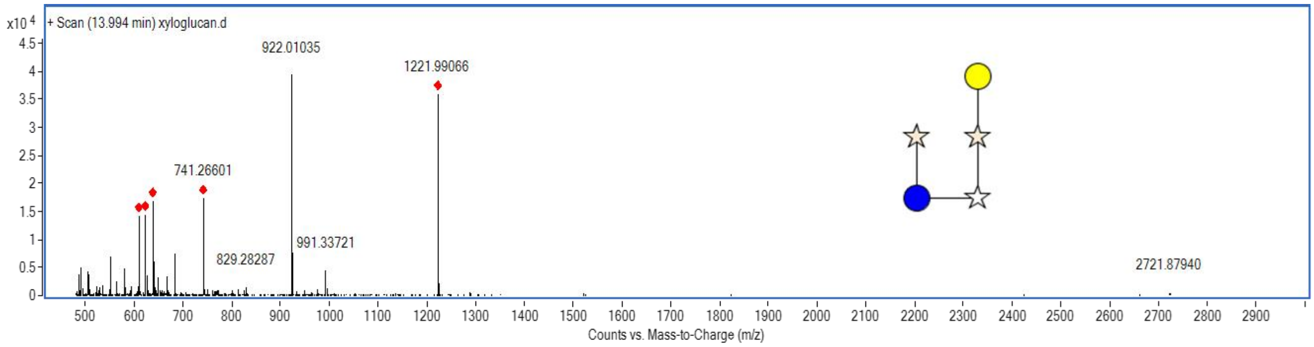

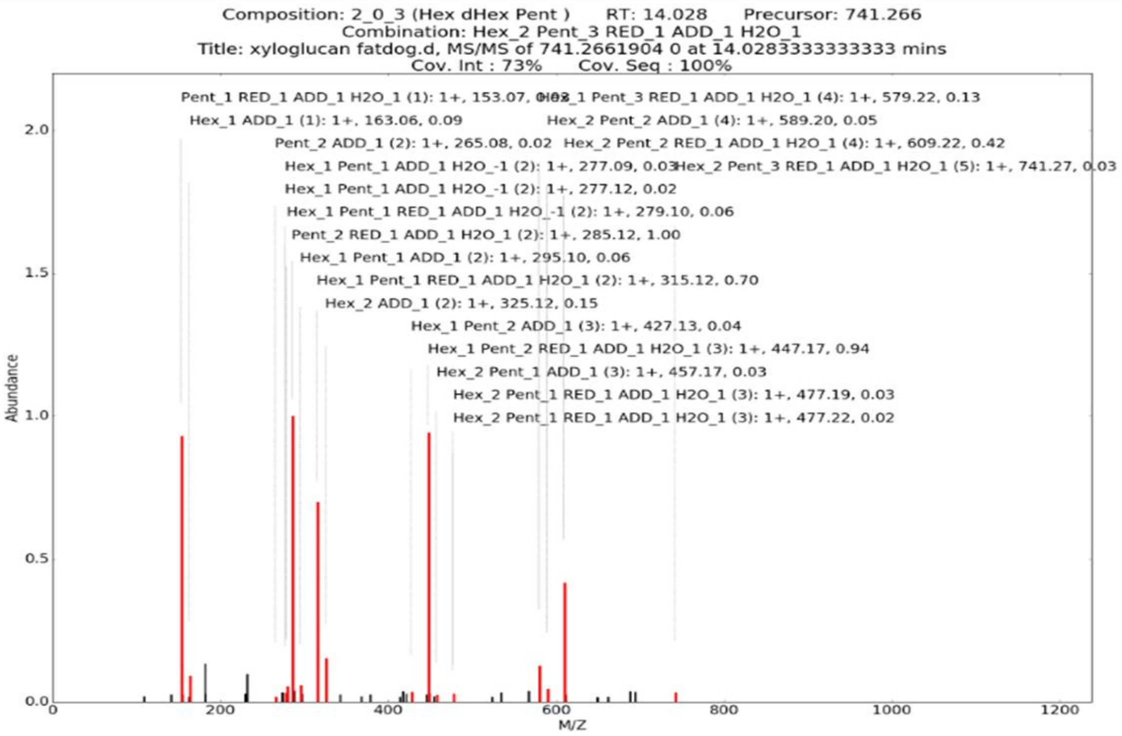

2Hex3Pent (b)

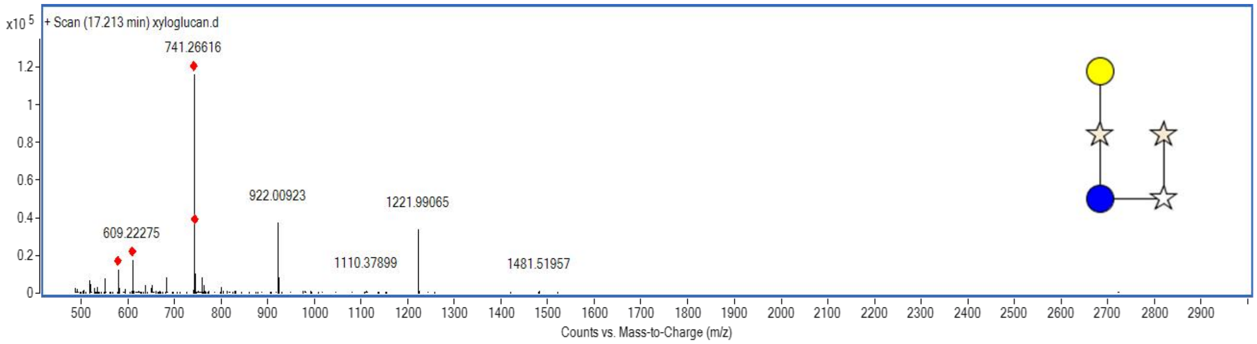
x10 5
+ Scan (17.213 min) xyloglucan.d
741.26616
922.00923
1221.99065
609.22275
1110.37899
1481.51957
Counts vs. Mass-to-Charge (m/z)

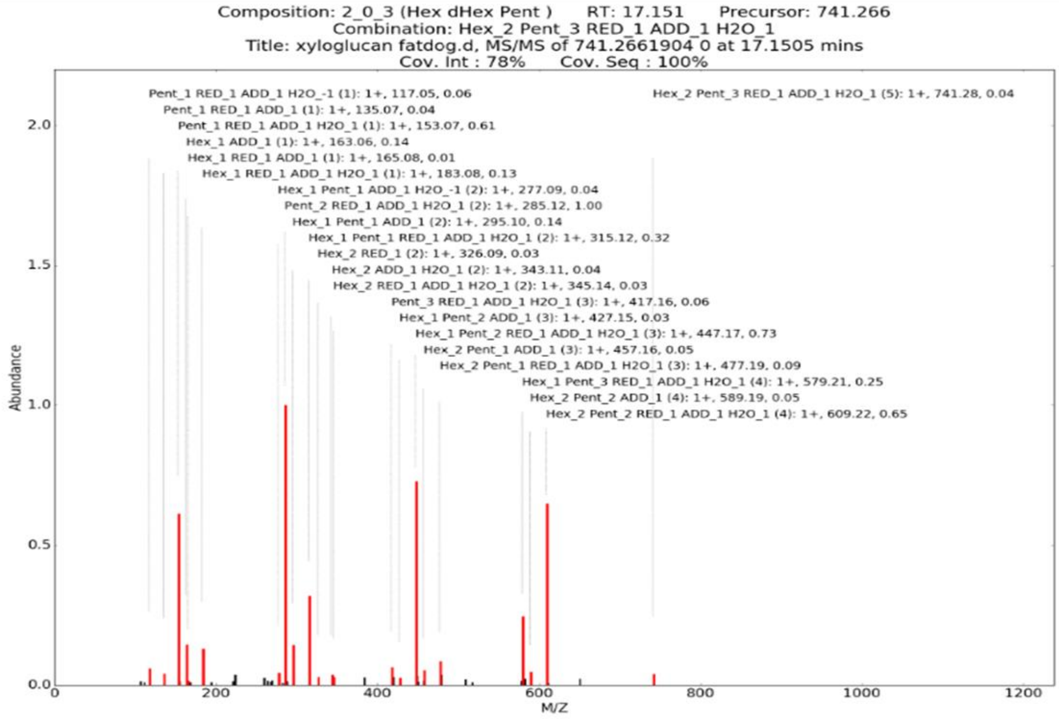
Composition: 2_0_3 (Hex dHex Pent) RT: 17.151 Precursor: 741.266
Combination: Hex_2 Pent_3 RED_1 ADD_1 H2O_1
Title: xyloglucan fatdog.d, MS/MS of 741.2661904 0 at 17.1505 mins
Cov. Int : 78% Cov. Seq : 100%
Pent_1 RED_1 ADD_1 H2O_-1 (1): 1+, 117.05, 0.06
Pent_1 RED_1 ADD_1 (1): 1+, 135.07, 0.04
Pent_1 RED_1 ADD_1 H2O_1 (1): 1+, 153.07, 0.61
Hex_1 ADD_1 (1): 1+, 163.06, 0.14
Hex_1 RED_1 ADD_1 (1): 1+, 165.08, 0.01
Hex_1 RED_1 ADD_1 H2O_1 (1): 1+, 183.08, 0.13
Hex_1 Pent_1 ADD_1 H2O_-1 (2): 1+, 277.09, 0.04
Pent_2 RED_1 ADD_1 H2O_1 (2): 1+, 285.12, 1.00
Hex_1 Pent_1 ADD_1 (2): 1+, 295.10, 0.14
Hex_1 Pent_1 RED_1 ADD_1 H2O_1 (2): 1+, 315.12, 0.32
Hex_2 RED_1 (2): 1+, 326.09, 0.03
Hex_2 ADD_1 H2O_1 (2): 1+, 343.11, 0.04
Hex_2 RED_1 ADD_1 H2O_1 (2): 1+, 345.14, 0.03
Pent_3 RED_1 ADD_1 H2O_1 (3): 1+, 417.16, 0.06
Hex_1 Pent_2 ADD_1 (3): 1+, 427.15, 0.03
Hex_1 Pent_2 RED_1 ADD_1 H2O_1 (3): 1+, 447.17, 0.73
Hex_2 Pent_1 ADD_1 (3): 1+, 457.16, 0.05
Hex_2 Pent_1 RED_1 ADD_1 H2O_1 (3): 1+, 477.19, 0.09
Hex_1 Pent_3 RED_1 ADD_1 H2O_1 (4): 1+, 579.21, 0.25
Hex_2 Pent_2 ADD_1 (4): 1+, 589.19, 0.05
Hex_2 Pent_2 RED_1 ADD_1 H2O_1 (4): 1+, 609.22, 0.65
Hex_2 Pent_3 RED_1 ADD_1 H2O_1 (5): 1+, 741.28, 0.04
Abundance
M/Z

2Hex3Pent (c)

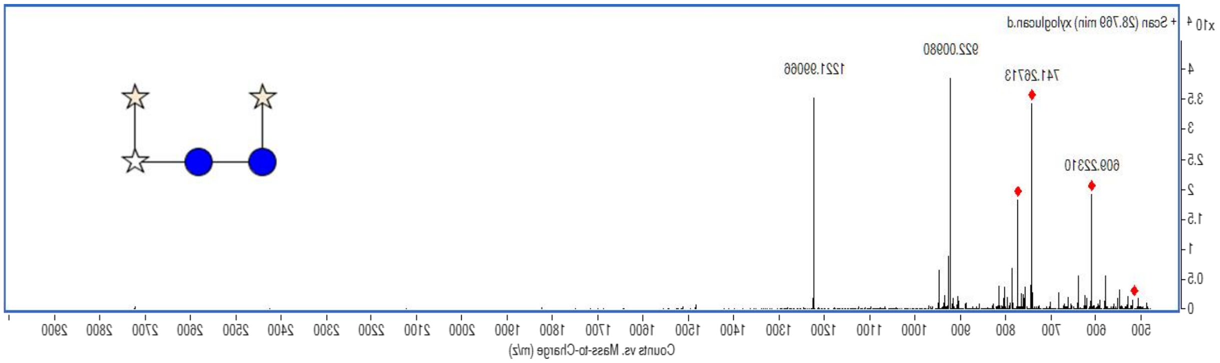

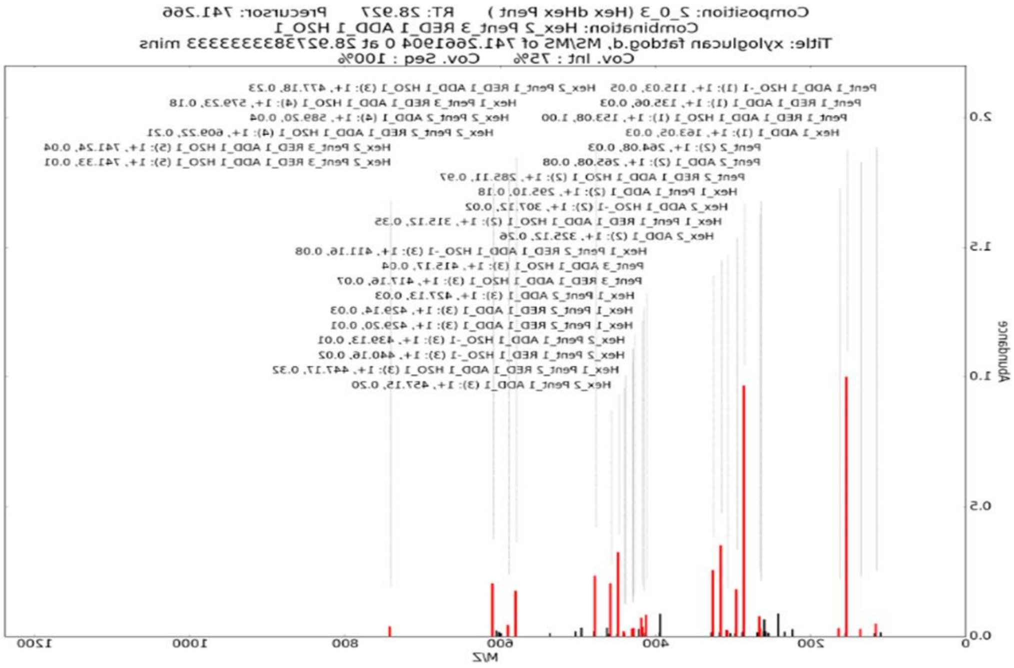

2Hex3Pent (d)

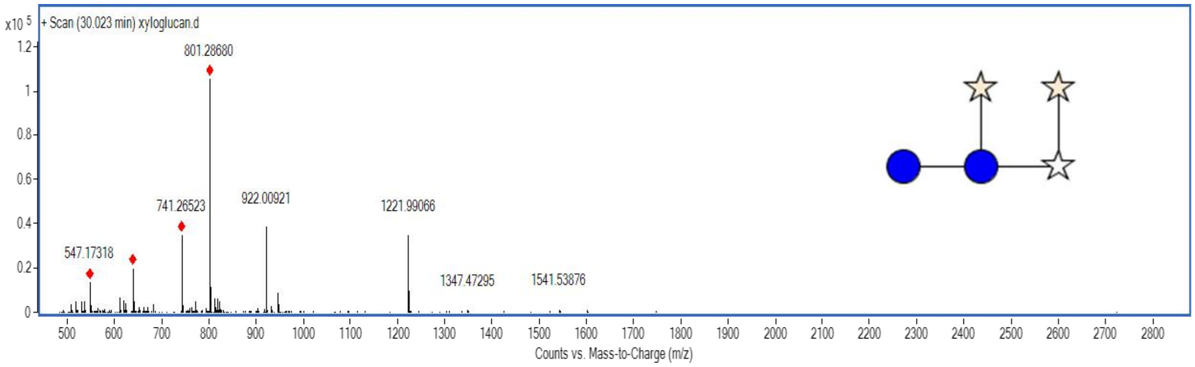
x10 5
+ Scan (30.023 min) xyloglucan.d
801.28680
741.26523
922.00921
1221.99066
547.17318
1347.47295
1541.53876
Counts vs. Mass-to-Charge (m/z)

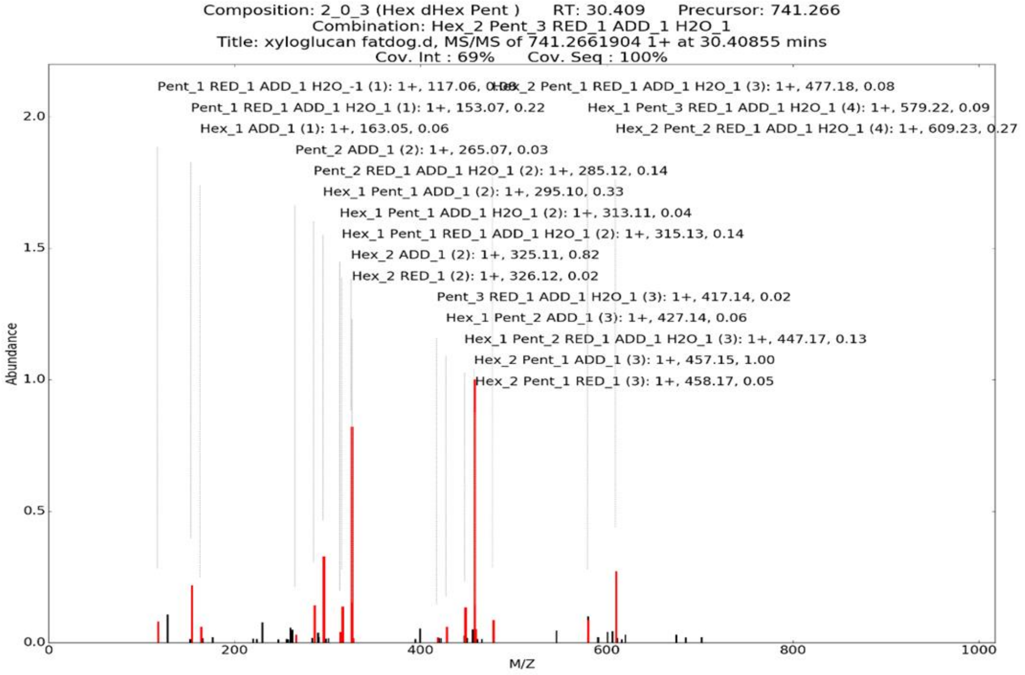
Composition: 2_0_3 (Hex dHex Pent) RT: 30.409 Precursor: 741.266
Combination: Hex_2 Pent_3 RED_1 ADD_1 H2O_1
Title: xyloglucan fatdog.d, MS/MS of 741.2661904 1+ at 30.40855 mins
Cov. Int : 69% Cov. Seq : 100%
Pent_1 RED_1 ADD_1 H2O_-1 (1): 1+, 117.06, 0.08
Hex_2 Pent_1 RED_1 ADD_1 H2O_1 (3): 1+, 477.18, 0.08
Pent_1 RED_1 ADD_1 H2O_1 (1): 1+, 153.07, 0.22
Hex_1 Pent_3 RED_1 ADD_1 H2O_1 (4): 1+, 579.22, 0.09
Hex_1 ADD_1 (1): 1+, 163.05, 0.06
Hex_2 Pent_2 RED_1 ADD_1 H2O_1 (4): 1+, 609.23, 0.27
Pent_2 ADD_1 (2): 1+, 265.07, 0.03
Pent_2 RED_1 ADD_1 H2O_1 (2): 1+, 285.12, 0.14
Hex_1 Pent_1 ADD_1 (2): 1+, 295.10, 0.33
Hex_1 Pent_1 ADD_1 H2O_1 (2): 1+, 313.11, 0.04
Hex_1 Pent_1 RED_1 ADD_1 H2O_1 (2): 1+, 315.13, 0.14
Hex_2 ADD_1 (2): 1+, 325.11, 0.82
Hex_2 RED_1 (2): 1+, 326.12, 0.02
Pent_3 RED_1 ADD_1 H2O_1 (3): 1+, 417.14, 0.02
Hex_1 Pent_2 ADD_1 (3): 1+, 427.14, 0.06
Hex_1 Pent_2 RED_1 ADD_1 H2O_1 (3): 1+, 447.17, 0.13
Hex_2 Pent_1 ADD_1 (3): 1+, 457.15, 1.00
Hex_2 Pent_1 RED_1 (3): 1+, 458.17, 0.05
Abundance
M/Z

2Hex3Pent (e)

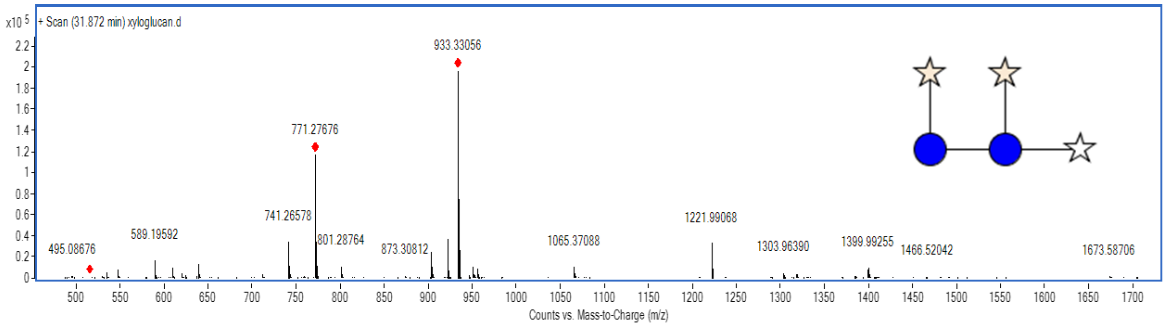

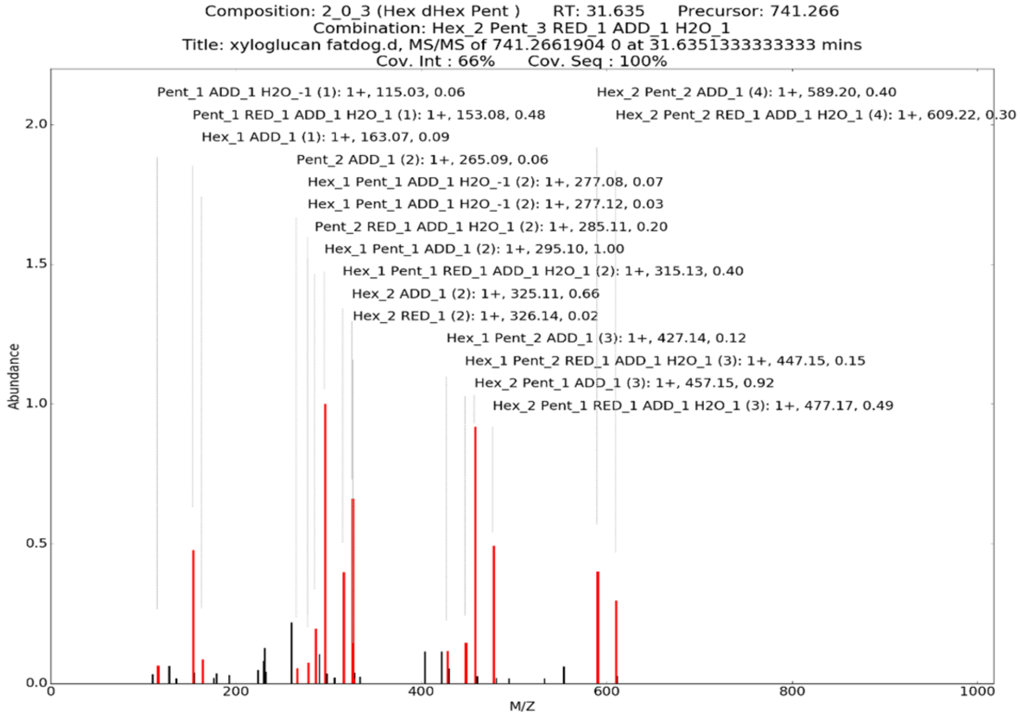

3Hex2Pent

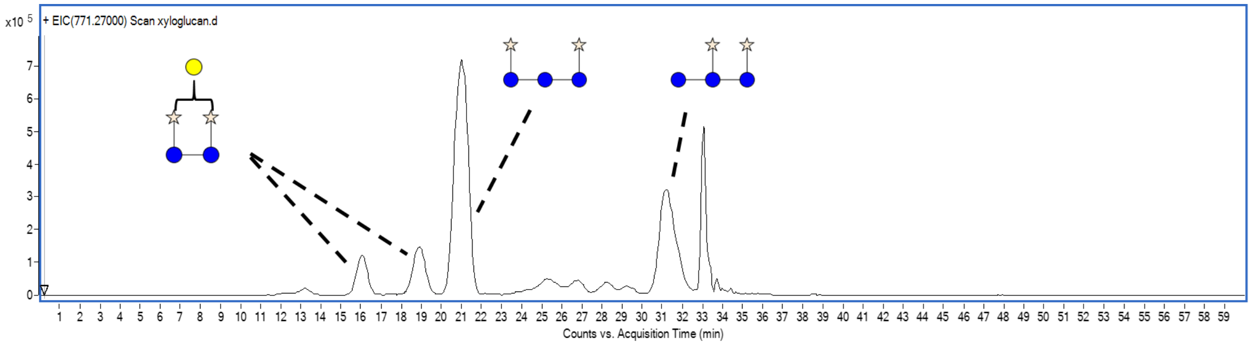

3Hex2Pent (a/b)

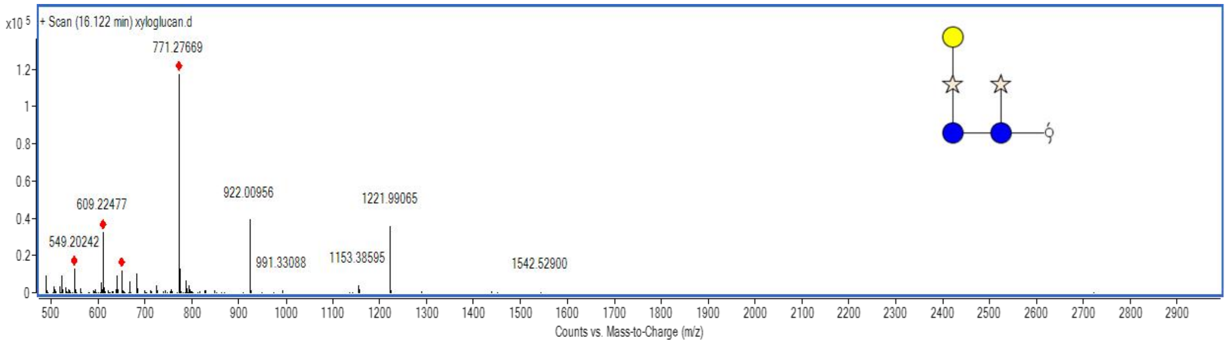

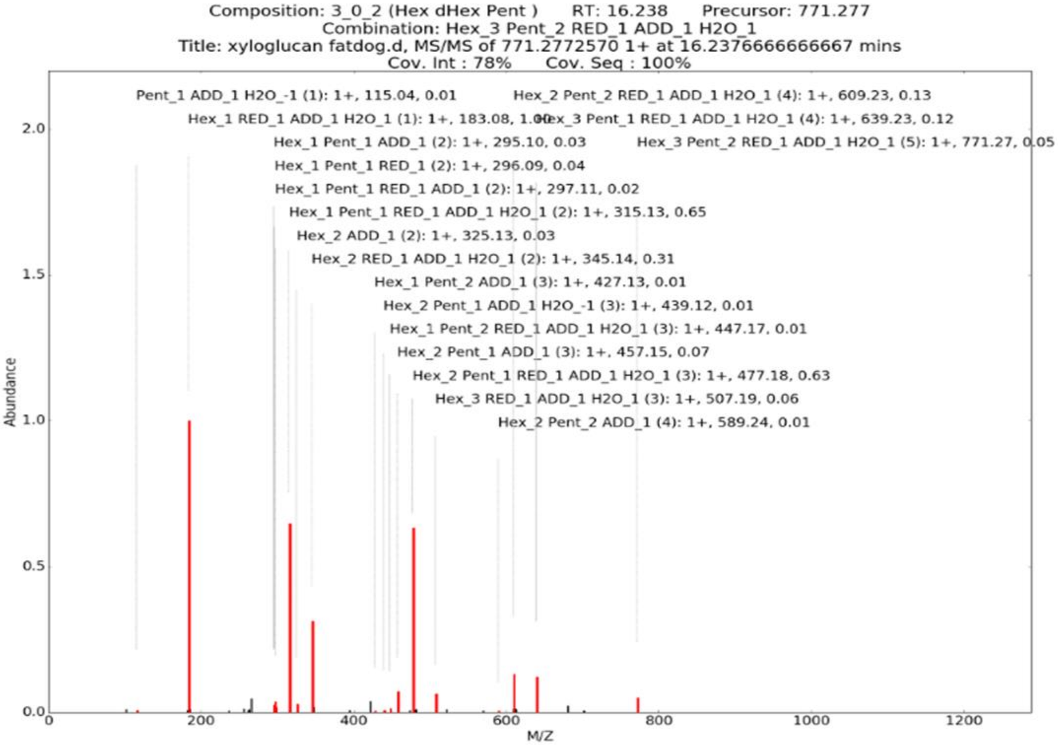

3Hex2Pent (c)

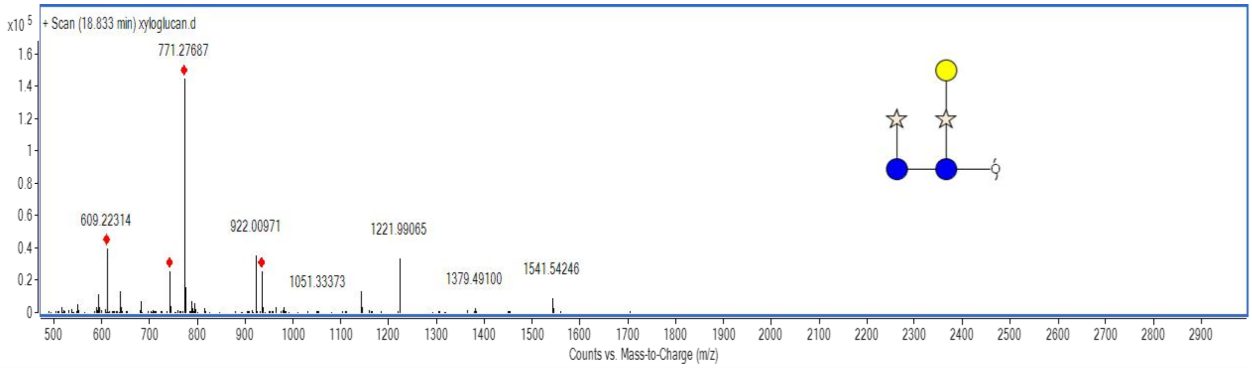
x10 5
+ Scan (18.833 min) xyloglucan.d
771.27687
609.22314
922.00971
1221.99065
1051.33373
1379.49100
1541.54246
Counts vs. Mass-to-Charge (m/z)

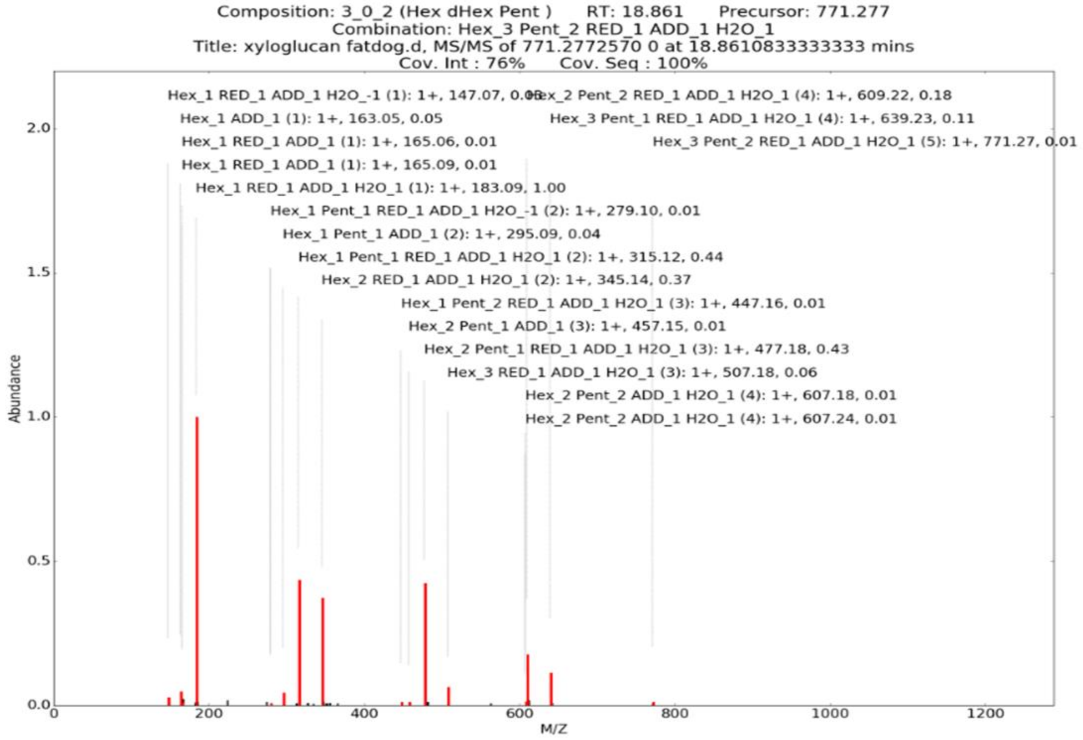
Composition: 3_0_2 (Hex dHex Pent) RT: 18.861 Precursor: 771.277
Combination: Hex_3 Pent_2 RED_1 ADD_1 H2O_1
Title: xyloglucan fatdog.d, MS/MS of 771.2772570 0 at 18.8610833333333 mins
Cov. Int : 76% Cov. Seq : 100%
Hex_1 RED_1 ADD_1 H2O_-1 (1): 1+, 147.07, 0.03
Hex_1 ADD_1 (1): 1+, 163.05, 0.05
Hex_1 RED_1 ADD_1 (1): 1+, 165.06, 0.01
Hex_1 RED_1 ADD_1 (1): 1+, 165.09, 0.01
Hex_1 RED_1 ADD_1 H2O_1 (1): 1+, 183.09, 1.00
Hex_1 Pent_1 RED_1 ADD_1 H2O_-1 (2): 1+, 279.10, 0.01
Hex_1 Pent_1 ADD_1 (2): 1+, 295.09, 0.04
Hex_1 Pent_1 RED_1 ADD_1 H2O_1 (2): 1+, 315.12, 0.44
Hex_2 RED_1 ADD_1 H2O_1 (2): 1+, 345.14, 0.37
Hex_1 Pent_2 RED_1 ADD_1 H2O_1 (3): 1+, 447.16, 0.01
Hex_2 Pent_1 ADD_1 (3): 1+, 457.15, 0.01
Hex_2 Pent_1 RED_1 ADD_1 H2O_1 (3): 1+, 477.18, 0.43
Hex_3 RED_1 ADD_1 H2O_1 (3): 1+, 507.18, 0.06
Hex_2 Pent_2 ADD_1 H2O_1 (4): 1+, 607.18, 0.01
Hex_2 Pent_2 ADD_1 H2O_1 (4): 1+, 607.24, 0.01
Hex_2 Pent_2 RED_1 ADD_1 H2O_1 (4): 1+, 609.22, 0.18
Hex_3 Pent_1 RED_1 ADD_1 H2O_1 (4): 1+, 639.23, 0.11
Hex_3 Pent_2 RED_1 ADD_1 H2O_1 (5): 1+, 771.27, 0.01
Abundance
M/Z

3Hex2Pent (d)

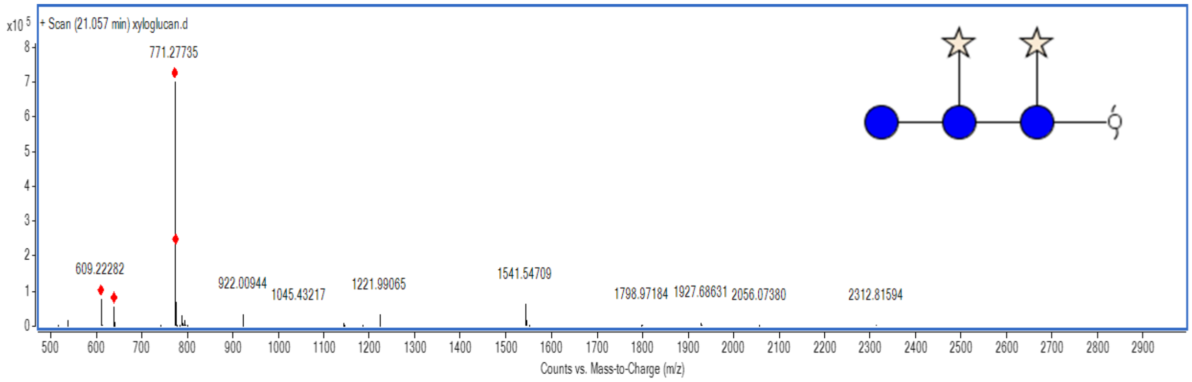

x10 5
+ Scan (21.057 min) xyloglucan.d
771.27735
609.22282
922.00944
1045.43217
1221.99065
1541.54709
1798.97184
1927.68631
2056.07380
2312.81594
Counts vs. Mass-to-Charge (m/z)

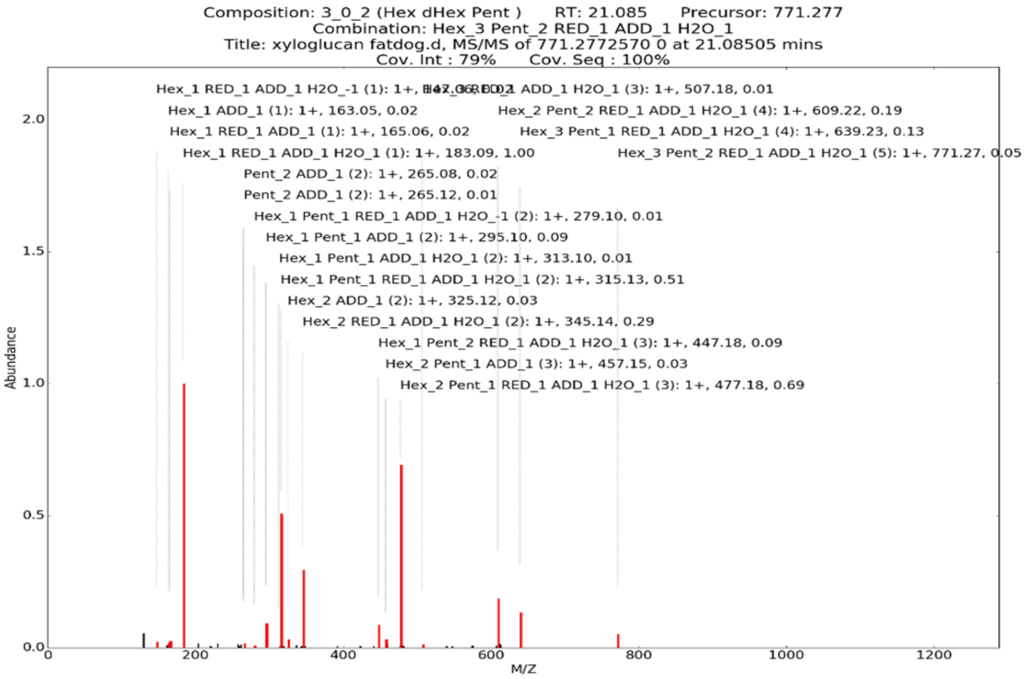

Composition: 3_0_2 (Hex dHex Pent) RT: 21.085 Precursor: 771.277
Combination: Hex_3 Pent_2 RED_1 ADD_1 H2O_1
Title: xyloglucan fatdog.d, MS/MS of 771.2772570 0 at 21.08505 mins
Cov. Int : 79% Cov. Seq : 100%
Hex_1 RED_1 ADD_1 H2O_-1 (1): 1+, 147.06, 0.02
Hex_2 RED_1 ADD_1 H2O_1 (3): 1+, 507.18, 0.01
Hex_1 ADD_1 (1): 1+, 163.05, 0.02
Hex_2 Pent_2 RED_1 ADD_1 H2O_1 (4): 1+, 609.22, 0.19
Hex_1 RED_1 ADD_1 (1): 1+, 165.06, 0.02
Hex_3 Pent_1 RED_1 ADD_1 H2O_1 (4): 1+, 639.23, 0.13
Hex_1 RED_1 ADD_1 H2O_1 (1): 1+, 183.09, 1.00
Hex_3 Pent_2 RED_1 ADD_1 H2O_1 (5): 1+, 771.27, 0.05
Pent_2 ADD_1 (2): 1+, 265.08, 0.02
Pent_2 ADD_1 (2): 1+, 265.12, 0.01
Hex_1 Pent_1 RED_1 ADD_1 H2O_-1 (2): 1+, 279.10, 0.01
Hex_1 Pent_1 ADD_1 (2): 1+, 295.10, 0.09
Hex_1 Pent_1 ADD_1 H2O_1 (2): 1+, 313.10, 0.01
Hex_1 Pent_1 RED_1 ADD_1 H2O_1 (2): 1+, 315.13, 0.51
Hex_2 ADD_1 (2): 1+, 325.12, 0.03
Hex_2 RED_1 ADD_1 H2O_1 (2): 1+, 345.14, 0.29
Hex_1 Pent_2 RED_1 ADD_1 H2O_1 (3): 1+, 447.18, 0.09
Hex_2 Pent_1 ADD_1 (3): 1+, 457.15, 0.03
Hex_2 Pent_1 RED_1 ADD_1 H2O_1 (3): 1+, 477.18, 0.69
Abundance
M/Z

3Hex2Pent (e)

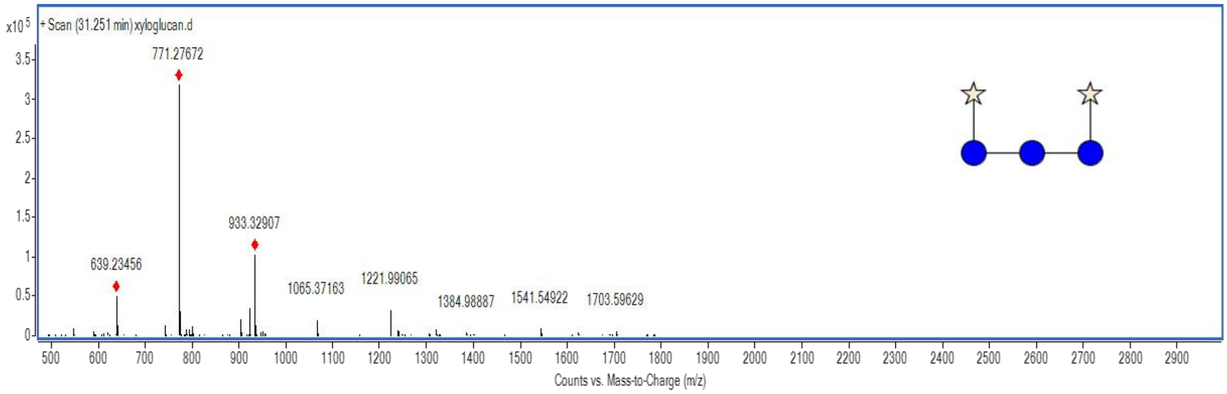

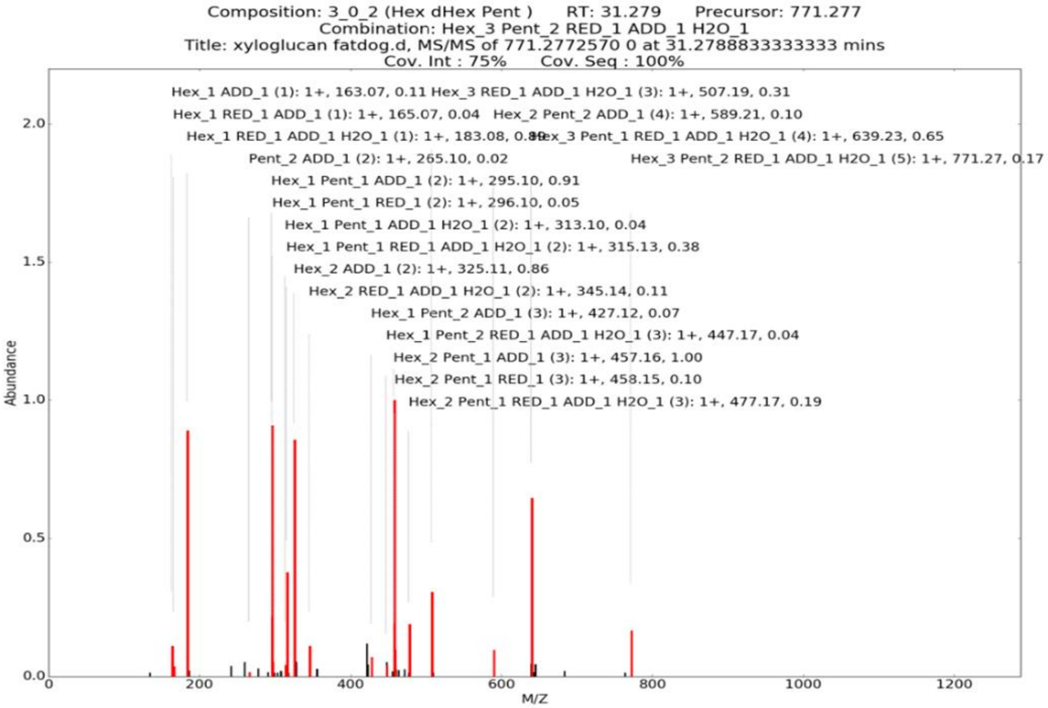

4Hex1Pent

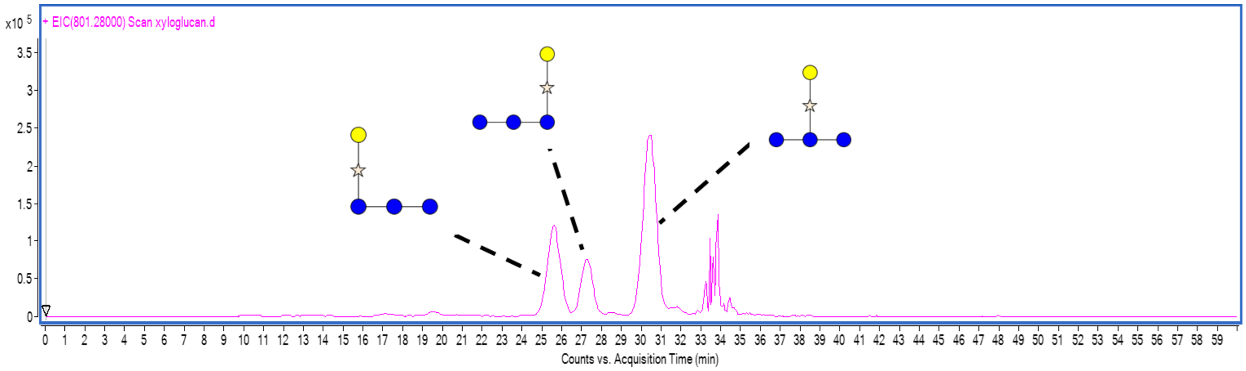

4Hex1Pent (a)

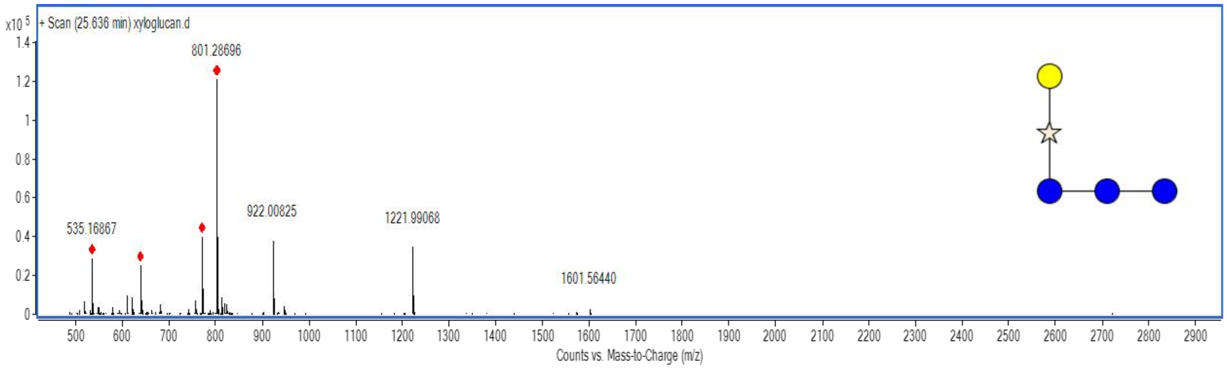

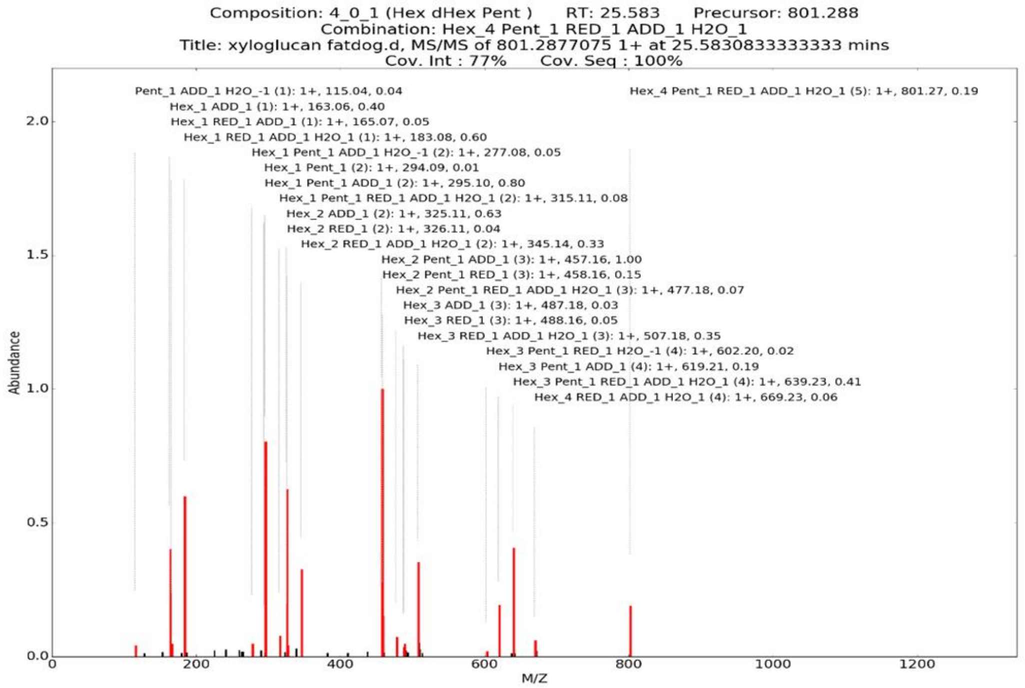

4Hex1Pent (b)

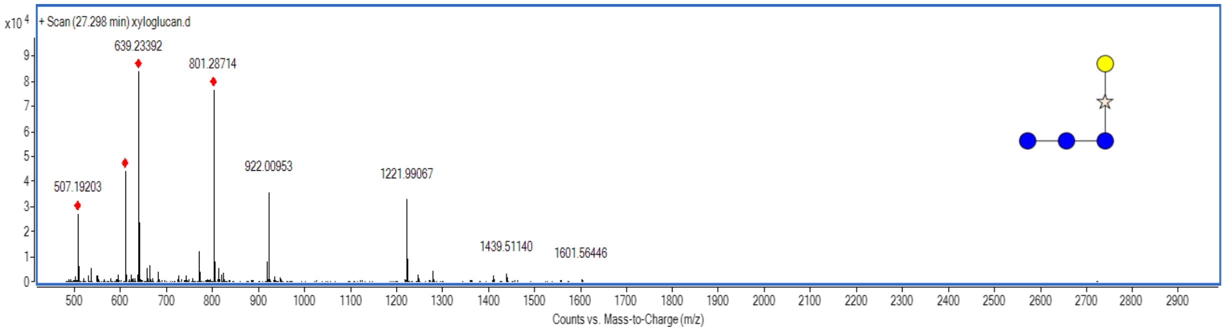

x10 4
+ Scan (27.298 min) xyloglucan.d
639.23392
801.28714
507.19203
922.00953
1221.99067
1439.51140
1601.56446
Counts vs. Mass-to-Charge (m/z)

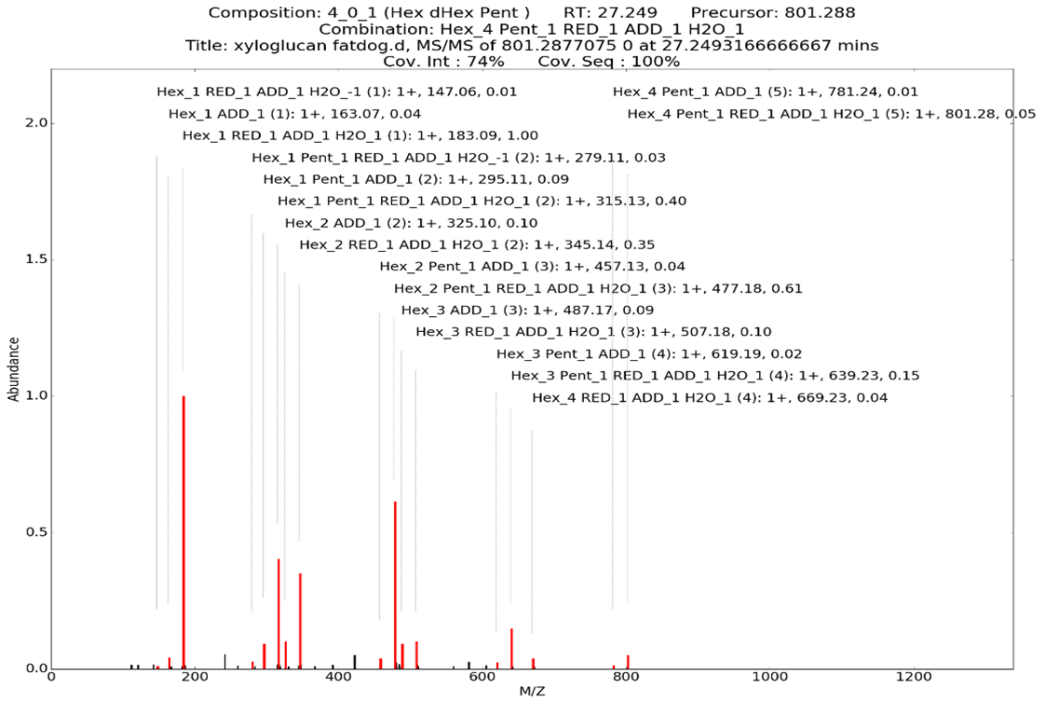

Composition: 4_0_1 (Hex dHex Pent) RT: 27.249 Precursor: 801.288
Combination: Hex_4 Pent_1 RED_1 ADD_1 H2O_1
Title: xyloglucan fatdog.d, MS/MS of 801.2877075 0 at 27.2493166666667 mins
Cov. Int : 74% Cov. Seq : 100%
Hex_1 RED_1 ADD_1 H2O_-1 (1): 1+, 147.06, 0.01
Hex_1 ADD_1 (1): 1+, 163.07, 0.04
Hex_1 RED_1 ADD_1 H2O_1 (1): 1+, 183.09, 1.00
Hex_1 Pent_1 RED_1 ADD_1 H2O_-1 (2): 1+, 279.11, 0.03
Hex_1 Pent_1 ADD_1 (2): 1+, 295.11, 0.09
Hex_1 Pent_1 RED_1 ADD_1 H2O_1 (2): 1+, 315.13, 0.40
Hex_2 ADD_1 (2): 1+, 325.10, 0.10
Hex_2 RED_1 ADD_1 H2O_1 (2): 1+, 345.14, 0.35
Hex_2 Pent_1 ADD_1 (3): 1+, 457.13, 0.04
Hex_2 Pent_1 RED_1 ADD_1 H2O_1 (3): 1+, 477.18, 0.61
Hex_3 ADD_1 (3): 1+, 487.17, 0.09
Hex_3 RED_1 ADD_1 H2O_1 (3): 1+, 507.18, 0.10
Hex_3 Pent_1 ADD_1 (4): 1+, 619.19, 0.02
Hex_3 Pent_1 RED_1 ADD_1 H2O_1 (4): 1+, 639.23, 0.15
Hex_4 RED_1 ADD_1 H2O_1 (4): 1+, 669.23, 0.04
Hex_4 Pent_1 ADD_1 (5): 1+, 781.24, 0.01
Hex_4 Pent_1 RED_1 ADD_1 H2O_1 (5): 1+, 801.28, 0.05
Abundance
M/Z

4Hex1Pent (c)

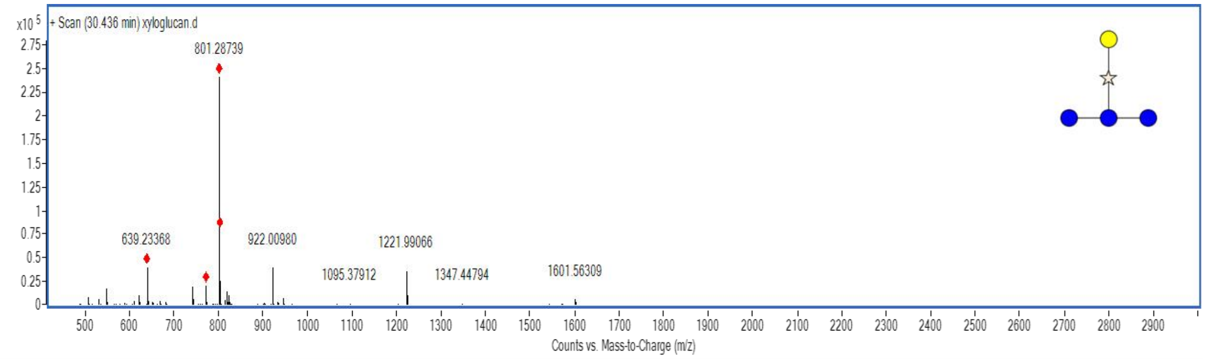
+ Scan (30.436 min) xyloglucan.d
801.28739
639.23368
922.00980
1221.99066
1095.37912
1347.44794
1601.56309
Counts vs. Mass-to-Charge (m/z)

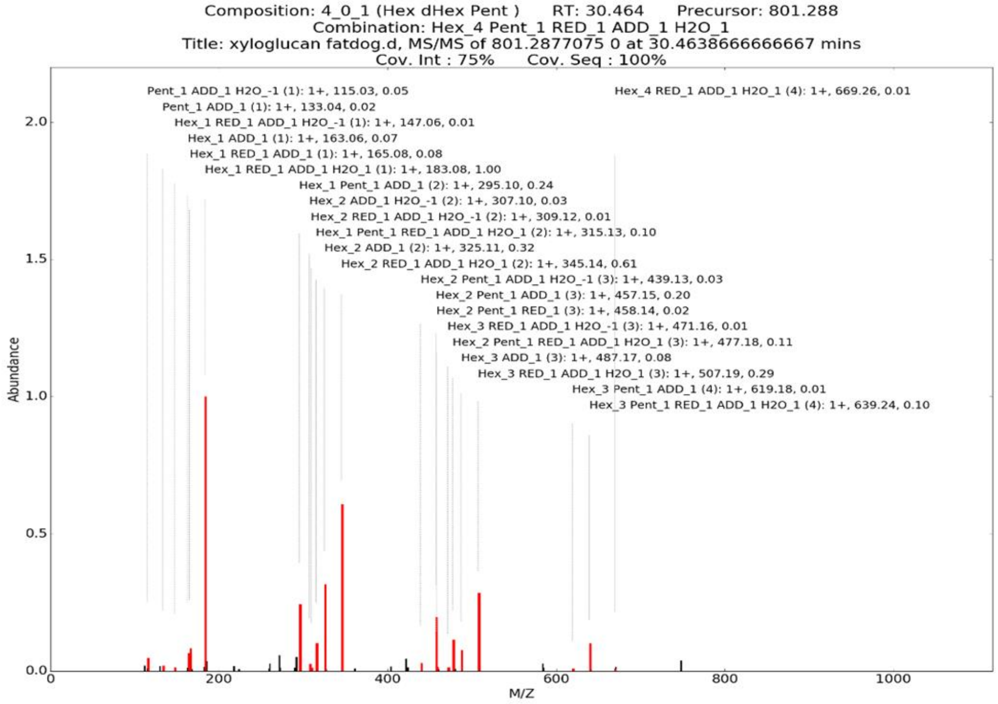
Composition: 4_0_1 (Hex dHex Pent) RT: 30.464 Precursor: 801.288
Combination: Hex_4 Pent_1 RED_1 ADD_1 H2O_1
Title: xyloglucan fatdog.d, MS/MS of 801.2877075 0 at 30.4638666666667 mins
Cov. Int : 75% Cov. Seq : 100%
Pent_1 ADD_1 H2O_-1 (1): 1+, 115.03, 0.05
Pent_1 ADD_1 (1): 1+, 133.04, 0.02
Hex_1 RED_1 ADD_1 H2O_-1 (1): 1+, 147.06, 0.01
Hex_1 ADD_1 (1): 1+, 163.06, 0.07
Hex_1 RED_1 ADD_1 (1): 1+, 165.08, 0.08
Hex_1 RED_1 ADD_1 H2O_1 (1): 1+, 183.08, 1.00
Hex_1 Pent_1 ADD_1 (2): 1+, 295.10, 0.24
Hex_2 ADD_1 H2O_-1 (2): 1+, 307.10, 0.03
Hex_2 RED_1 ADD_1 H2O_-1 (2): 1+, 309.12, 0.01
Hex_1 Pent_1 RED_1 ADD_1 H2O_1 (2): 1+, 315.13, 0.10
Hex_2 ADD_1 (2): 1+, 325.11, 0.32
Hex_2 RED_1 ADD_1 H2O_1 (2): 1+, 345.14, 0.61
Hex_2 Pent_1 ADD_1 H2O_-1 (3): 1+, 439.13, 0.03
Hex_2 Pent_1 ADD_1 (3): 1+, 457.15, 0.20
Hex_2 Pent_1 RED_1 (3): 1+, 458.14, 0.02
Hex_3 RED_1 ADD_1 H2O_-1 (3): 1+, 471.16, 0.01
Hex_2 Pent_1 RED_1 ADD_1 H2O_1 (3): 1+, 477.18, 0.11
Hex_3 ADD_1 (3): 1+, 487.17, 0.08
Hex_3 RED_1 ADD_1 H2O_1 (3): 1+, 507.19, 0.29
Hex_3 Pent_1 ADD_1 (4): 1+, 619.18, 0.01
Hex_3 Pent_1 RED_1 ADD_1 H2O_1 (4): 1+, 639.24, 0.10
Hex_4 RED_1 ADD_1 H2O_1 (4): 1+, 669.26, 0.01
Abundance
M/Z

3Hex3Pent

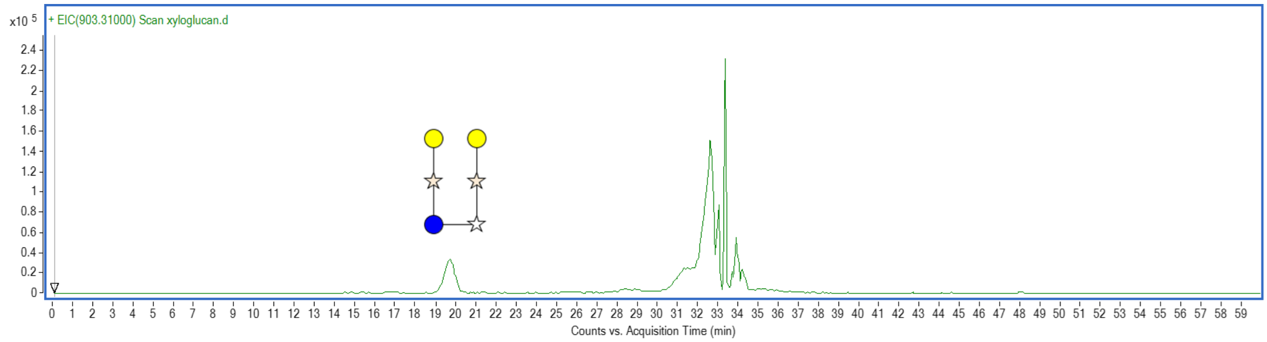

3Hex3Pent

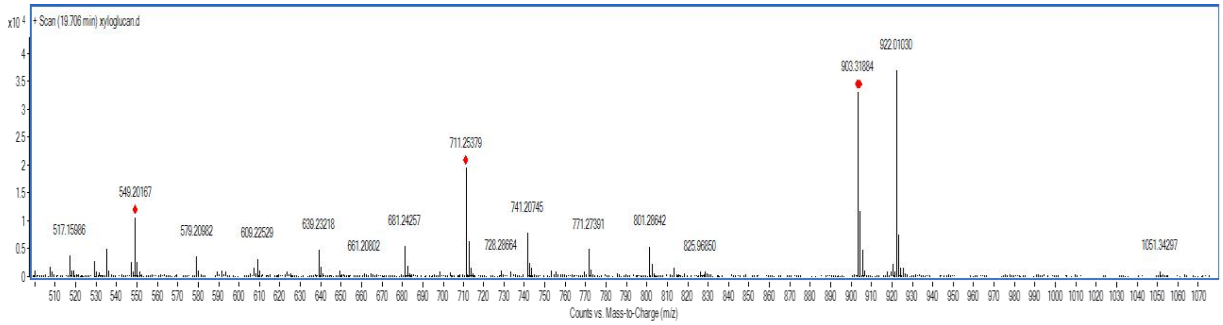

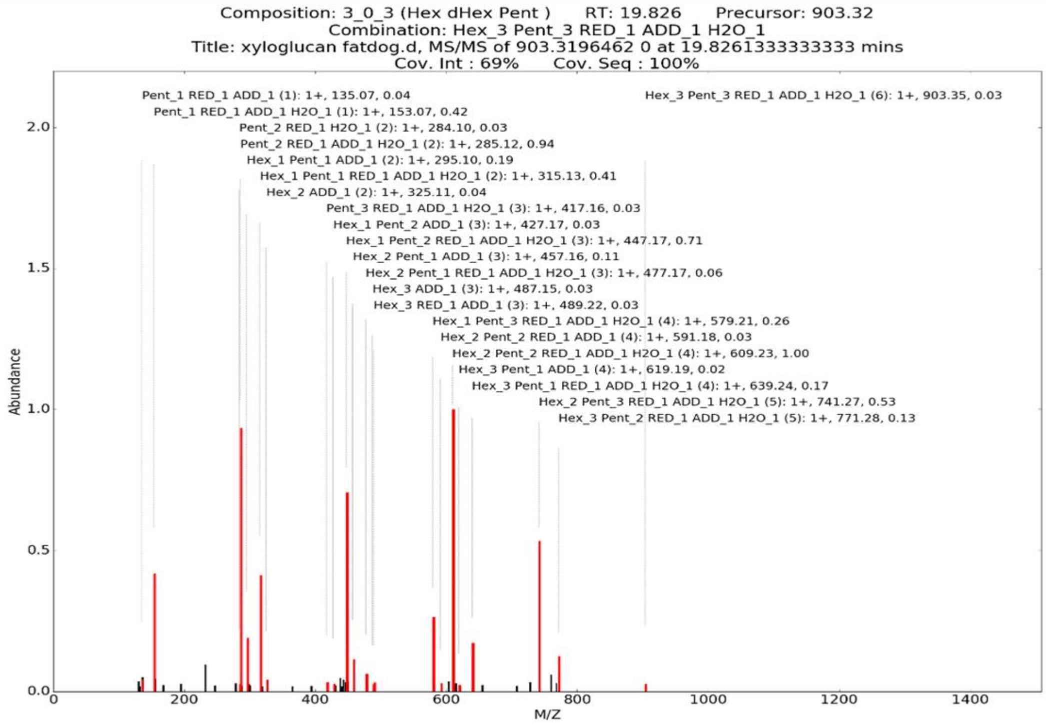

4Hex2Pent

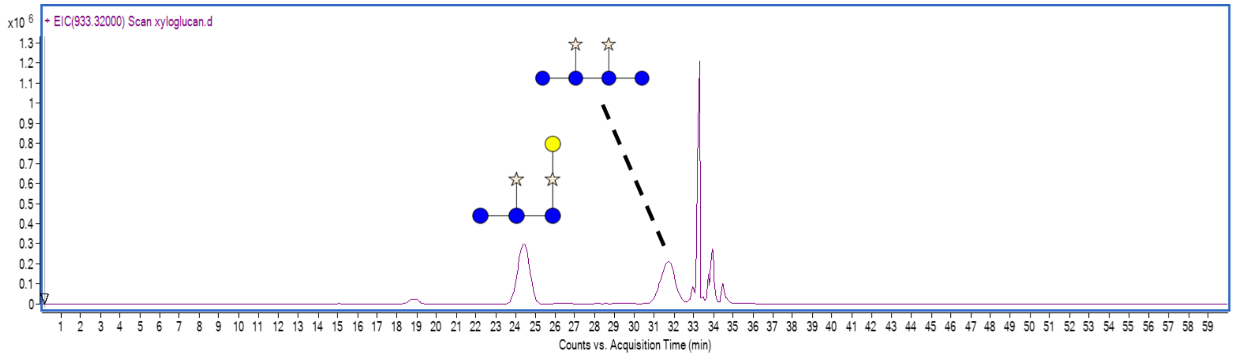

4Hex2Pent (a)

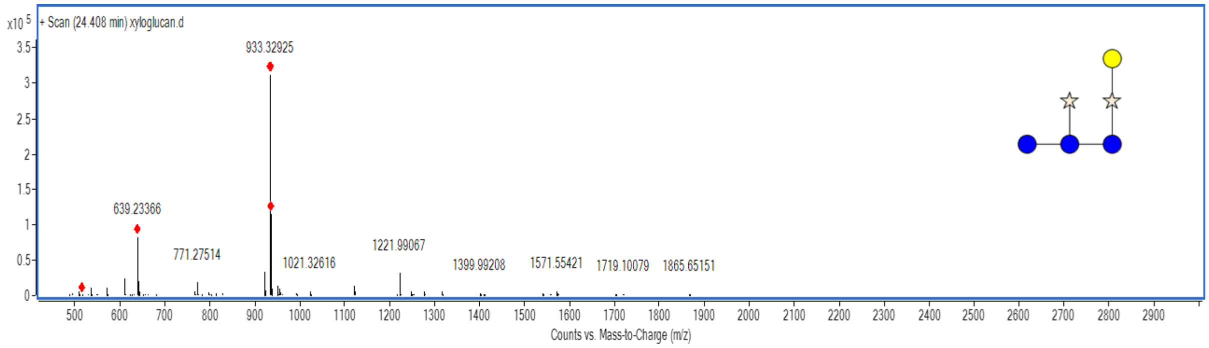

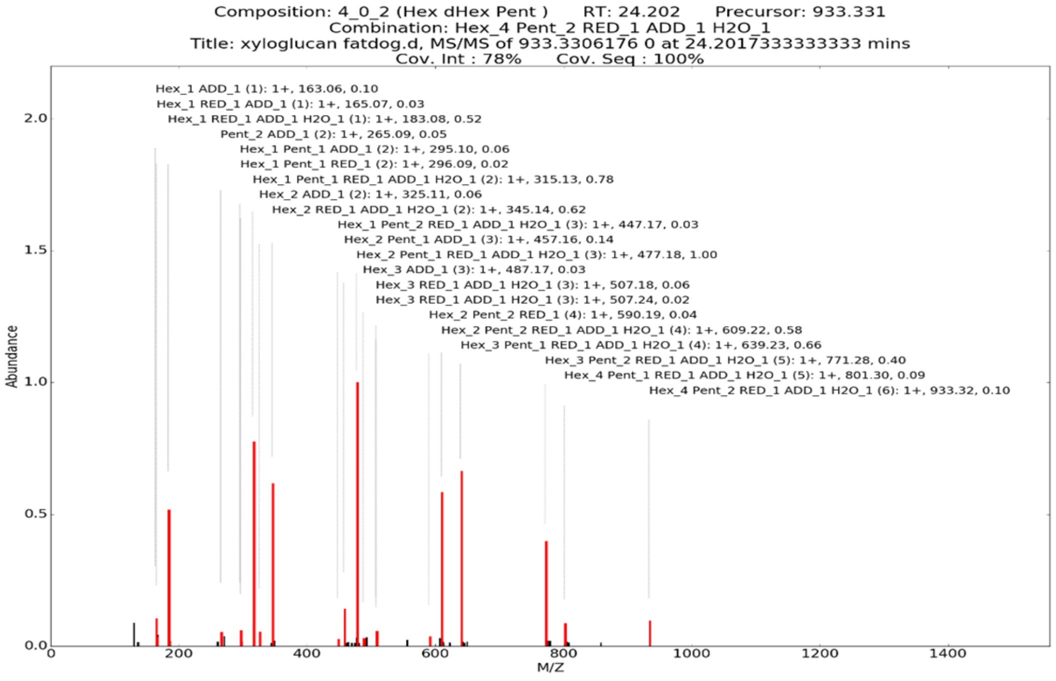

4Hex2Pent (b)

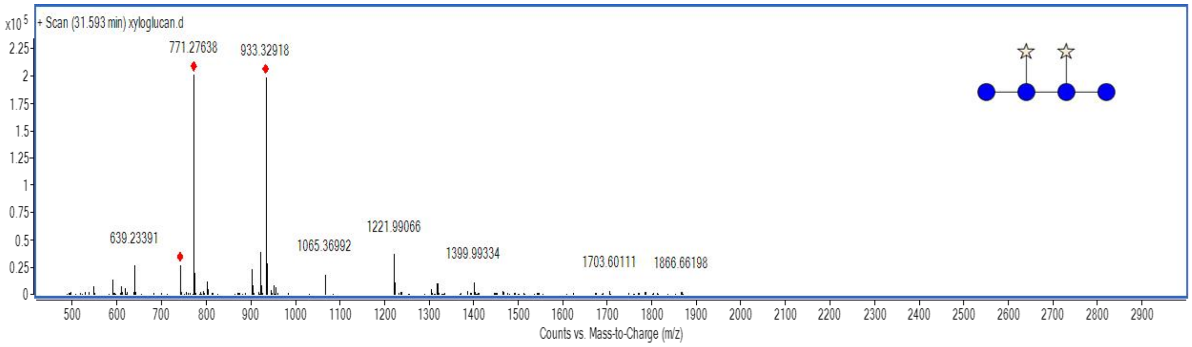
x10 5
+ Scan (31.593 min) xyloglucan.d
771.27638
933.32918
639.23391
1065.36992
1221.99066
1399.99334
1703.60111
1866.66198
Counts vs. Mass-to-Charge (m/z)

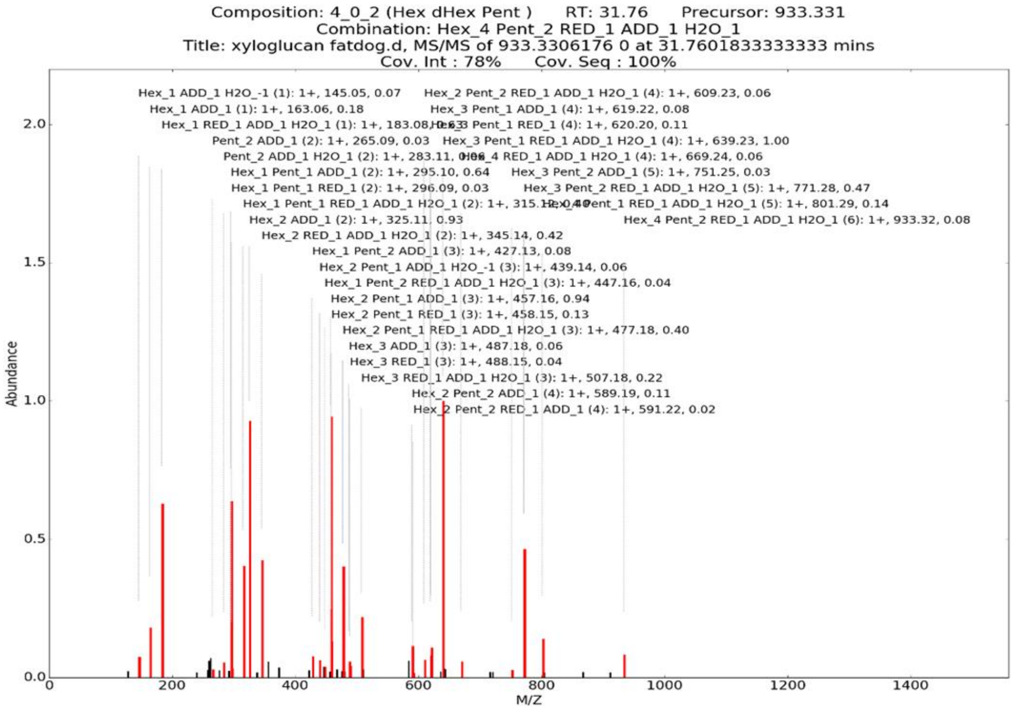
Composition: 4_0_2 (Hex dHex Pent) RT: 31.76 Precursor: 933.331
Combination: Hex_4 Pent_2 RED_1 ADD_1 H2O_1
Title: xyloglucan fatdog.d, MS/MS of 933.3306176 0 at 31.7601833333333 mins
Cov. Int : 78% Cov. Seq : 100%
Hex_1 ADD_1 H2O_-1 (1): 1+, 145.05, 0.07
Hex_1 ADD_1 (1): 1+, 163.06, 0.18
Hex_1 RED_1 ADD_1 H2O_1 (1): 1+, 183.08,
Pent_2 ADD_1 (2): 1+, 265.09, 0.03
Pent_2 ADD_1 H2O_1 (2): 1+, 283.11,
Hex_1 Pent_1 ADD_1 (2): 1+, 295.10, 0.64
Hex_1 Pent_1 RED_1 (2): 1+, 296.09, 0.03
Hex_1 Pent_1 RED_1 ADD_1 H2O_1 (2): 1+, 315.1
Hex_2 ADD_1 (2): 1+, 325.11, 0.93
Hex_2 RED_1 ADD_1 H2O_1 (2): 1+, 345.14, 0.42
Hex_1 Pent_2 ADD_1 (3): 1+, 427.13, 0.08
Hex_2 Pent_1 ADD_1 H2O_-1 (3): 1+, 439.14, 0.06
Hex_1 Pent_2 RED_1 ADD_1 H2O_1 (3): 1+, 447.16, 0.04
Hex_2 Pent_1 ADD_1 (3): 1+, 457.16, 0.94
Hex_2 Pent_1 RED_1 (3): 1+, 458.15, 0.13
Hex_2 Pent_1 RED_1 ADD_1 H2O_1 (3): 1+, 477.18, 0.40
Hex_3 ADD_1 (3): 1+, 487.18, 0.06
Hex_3 RED_1 (3): 1+, 488.15, 0.04
Hex_3 RED_1 ADD_1 H2O_1 (3): 1+, 507.18, 0.22
Hex_2 Pent_2 ADD_1 (4): 1+, 589.19, 0.11
Hex_2 Pent_2 RED_1 ADD_1 (4): 1+, 591.22, 0.02
Hex_2 Pent_2 RED_1 ADD_1 H2O_1 (4): 1+, 609.23, 0.06
Hex_3 Pent_1 ADD_1 (4): 1+, 619.22, 0.08
Pent_1 RED_1 (4): 1+, 620.20, 0.11
Hex_3 Pent_1 RED_1 ADD_1 H2O_1 (4): 1+, 639.23, 1.00
RED_1 ADD_1 H2O_1 (4): 1+, 669.24, 0.06
Hex_3 Pent_2 ADD_1 (5): 1+, 751.25, 0.03
Hex_3 Pent_2 RED_1 ADD_1 H2O_1 (5): 1+, 771.28, 0.47
Pent_1 RED_1 ADD_1 H2O_1 (5): 1+, 801.29, 0.14
Hex_4 Pent_2 RED_1 ADD_1 H2O_1 (6): 1+, 933.32, 0.08
Abundance
M/Z
