# Supplementary material for: A nonenzymatic method for cleaving polysaccharides to yield oligosaccharides for structural analysis
Source: Nat Commun. 2020 Aug 7;11:3963. doi: 10.1038/s41467-020-17778-1 (PMC7414865; doi:10.1038/s41467-020-17778-1)
Supplement: Supplementary file 5 — Supplementary Data 3 [file 41467_2020_17778_MOESM5_ESM.pdf]

Supplementary Data 3

Polysaccharide fingerprinting library of 14 polysaccharides. Compounds are referred to as their acryonym: Hexose/Hex, Pentose/Pent, O-methylated Glucuronic Acid/GlcAOMe. Polysaccharides are abbreviated: Curdlan/Curd, Cellulose/Cell, β-Glucan/β-Glc, Lichenan/Lich, Galactan/Gal, Mannan/Man, Glucomannan/GlcMan, Galactomannan/GalMan, Arabinan/Ara, Xylan/Xyl, Arabinoxylan/AraXyl, Amylose/Amy, Amylopectin/AmyP, Xyloglucan/XylGlc. An “X” denotes the presence of the corresponding oligosaccharide from the FITDOG depolymerization of the parent polysaccharide.

Mass (Dal)	RT (min)	Compound	Curdlan	Cellulose	β-Glucan	Lichenan	Galactan	Mannan	Glucomannan
504.169	1.51	3Hex						x	x
504.169	1.78	3Hex							
504.169	1.97	3Hex				x			x
414.137	2	3Pent							
474.158	2.01	2Hex:1Pent						x	
504.169	2.34	3Hex					x		
504.169	2.56	3Hex							
666.222	2.58	4Hex				x			
474.158	2.69	2Hex:1Pent					x		
474.158	2.91	2Hex:1Pent					x		
666.222	2.91	4Hex				x			
414.137	2.96	3Pent							
666.222	3.36	4Hex				x			
666.222	3.51	4Hex						x	x
504.169	3.68	3Hex							
414.137	3.94	3Pent							
666.222	4.01	4Hex						x	
414.137	4.4	3Pent							
666.222	4.41	4Hex							
666.222	4.51	4Hex						x	
414.137	4.77	3Pent							
666.222	4.94	4Hex						x	
414.137	4.97	3Pent							
636.211	5.84	3Hex:1Pent							
666.222	5.91	4Hex							x
636.211	5.99	3Hex:1Pent					x		
636.211	6.3	3Hex:1Pent						x	x
504.169	6.87	3Hex							x
474.158	6.88	2Hex:1Pent							
636.211	7.05	3Hex:1Pent					x		
636.211	7.43	3Hex:1Pent						x	x
666.222	7.5	4Hex					x		
474.158	7.68	2Hex:1Pent							
546.18	8.01	4Pent							
666.222	8.18	4Hex					x		
666.222	8.34	4Hex							
504.169	8.71	3Hex							x
636.211	8.72	3Hex:1Pent					x		x
828.274	9.15	5Hex						x	x
828.274	9.2	5Hex							x
666.222	9.32	4Hex					x		
828.274	9.5	5Hex							
636.211	9.52	3Hex:1Pent							
1152.38	9.59	7Hex				x			
990.327	9.72	6Hex				x			
414.137	9.83	3Pent							
636.211	9.91	3Hex:1Pent							
1152.38	9.99	7Hex				x			
504.169	10.05	3Hex							x
636.211	10.1	3Hex:1Pent					x		
828.274	10.13	5Hex							
546.18	10.19	4Pent							

990.327	10.28	6Hex				x			
1152.38	10.5	7Hex				x			
828.274	10.54	5Hex							
546.18	10.55	4Pent							
828.274	10.68	5Hex							x
798.264	10.78	4Hex:1Pent					x		
474.158	10.94	2Hex:1Pent							
798.264	10.97	4Hex:1Pent						x	x
666.222	10.98	4Hex							
798.264	11.03	4Hex:1Pent					x		
546.18	11.03	4Pent							
828.274	11.06	5Hex							
1314.433	11.16	8Hex				x			
828.274	11.19	5Hex					x		
546.18	11.2	4Pent							
828.274	11.25	5Hex							
504.169	11.38	3Hex			x				
798.264	11.38	4Hex:1Pent							x
798.264	11.5	4Hex:1Pent					x		
546.18	11.58	4Pent							
546.18	11.66	4Pent							
798.264	11.85	4Hex:1Pent							
990.327	11.93	6Hex						x	x
960.317	12.14	5Hex:1Pent					x		
798.264	12.15	4Hex:1Pent							
546.18	12.16	4Pent							
636.211	12.21	3Hex:1Pent							x
990.327	12.23	6Hex							
1152.38	12.24	7Hex				x			
546.18	12.26	4Pent							
960.317	12.27	5Hex:1Pent					x		
474.158	12.39	2Hex:1Pent			x				
678.222	12.39	5Pent							
990.327	12.4	6Hex					x		
990.327	12.46	6Hex							x
576.19	12.47	3Pent1Hex							
798.264	12.5	4Hex:1Pent							
636.211	12.51	3Hex:1Pent							x
960.317	12.55	5Hex:1Pent					x		
504.169	12.57	3Hex	x						
990.327	12.6	6Hex							
960.317	12.65	5Hex:1Pent						x	
828.274	12.65	5Hex				x	x		
678.222	12.77	5Pent							
474.158	12.83	2Hex:1Pent			x				
828.274	12.85	5Hex							x
990.327	12.86	6Hex							
960.317	12.88	5Hex:1Pent							
636.211	13.03	3Hex:1Pent							x
960.317	13.04	5Hex:1Pent							
604.19	13.06	3Pent:1GlcAOMe							
1152.38	13.1	7Hex					x	x	
1152.38	13.11	7Hex							x
1092.359	13.22	6Hex:1Pent					x		
960.317	13.23	5Hex:1Pent							
828.274	13.47	5Hex				x			
1152.38	13.51	7Hex							
678.222	13.52	5Pent							
960.317	13.62	5Hex:1Pent							
1152.38	13.65	7Hex							x
1314.433	13.68	8Hex					x		
606.201	13.71	2Hex:2Pent							
504.169	13.73	3Hex		x	x	x			
828.274	13.73	5Hex							x
678.222	13.81	5Pent							
1092.359	13.83	6Hex:1Pent						x	
1092.359	13.84	6Hex:1Pent							

990.327	13.85	6Hex					x
1284.422	13.86	7Hex:1Pent			x		
604.19	14	3Pent:1GlcAOMe					
810.264	14.03	6Pent					
828.274	14.03	5Hex					x
678.222	14.03	5Pent					
1314.433	14.06	8Hex				x	
678.222	14.11	5Pent					
666.222	14.13	4Hex		x			
798.264	14.2	4Hex:1Pent					x
474.158	14.21	2Hex:1Pent	x				
678.222	14.21	5Pent					
1092.359	14.24	6Hex:1Pent					
604.19	14.27	3Pent:1GlcAOMe					
1314.433	14.34	8Hex					
678.222	14.36	5Pent					
990.327	14.38	6Hex		x			x
474.158	14.39	2Hex:1Pent	x				
678.222	14.5	5Pent					
1152.38	14.53	7Hex					x
1284.422	14.56	7Hex:1Pent				x	
1476.486	14.62	9Hex			x		
1314.433	14.67	8Hex					
1092.359	14.7	6Hex:1Pent					
1476.486	14.75	9Hex				x	
798.264	14.76	4Hex:1Pent					x
828.274	14.81	5Hex					x
678.222	14.86	5Pent					
1284.422	14.9	7Hex:1Pent					
1314.433	14.9	8Hex					
1446.475	14.91	8Hex:1Pent			x		
768.253	14.92	3Hex:2Pent					
810.264	14.97	6Pent					
678.222	14.98	5Pent					
1314.433	15.08	8Hex					
1476.486	15.1	9Hex					
810.264	15.11	6Pent					
1314.433	15.14	8Hex					x
768.253	15.15	3Hex:2Pent					
504.169	15.17	3Hex	x				
990.327	15.19	6Hex					x
798.264	15.21	4Hex:1Pent					x
504.169	15.24	3Hex		x			
1314.433	15.27	8Hex					
546.18	15.33	4Pent					
1446.475	15.33	8Hex:1Pent				x	
828.274	15.34	5Hex					x
810.264	15.36	6Pent					
1284.422	15.4	7Hex:1Pent					
828.274	15.45	5Hex		x			
1152.38	15.49	7Hex					x
960.317	15.56	5Hex:1Pent					x
810.264	15.57	6Pent					
1314.433	15.58	8Hex					
810.264	15.65	6Pent					
1152.38	15.65	7Hex		x			
942.307	15.69	7Pent					
636.211	15.72	3Hex:1Pent					
1152.38	15.72	7Hex					x
678.222	15.82	5Pent					
828.274	15.85	5Hex					x
1476.486	15.97	9Hex					x
930.306	16	4Hex:2Pent					
990.327	16	6Hex					x
798.264	16.05	4Hex:1Pent					x
1284.422	16.06	7Hex:1Pent					
810.264	16.13	6Pent					

990.327	16.23	6Hex				x			x
828.274	16.32	5Hex							x
810.264	16.32	6Pent							
1284.422	16.36	7Hex:1Pent							
990.327	16.47	6Hex							x
604.19	16.47	3Pent:1GlcAOMe							
810.264	16.52	6Pent							
636.211	16.55	3Hex:1Pent							x
942.307	16.61	7Pent							
990.327	16.61	6Hex				x			
828.274	16.62	5Hex							x
636.211	16.66	3Hex:1Pent							
1314.433	16.7	8Hex				x			
1314.433	16.72	8Hex							x
798.264	16.73	4Hex:1Pent							x
678.222	16.76	5Pent							
798.264	16.84	4Hex:1Pent							
636.211	16.85	3Hex:1Pent							x
828.274	16.91	5Hex							x
1152.38	17.15	7Hex							x
678.222	17.15	5Pent							
810.264	17.21	6Pent							
1152.38	17.24	7Hex				x			
960.317	17.25	5Hex:1Pent							x
1638.538	17.25	10Hex							
942.307	17.26	7Pent							
636.211	17.32	3Hex:1Pent							
942.307	17.44	7Pent							
666.222	17.46	4Hex			x				
1314.433	17.49	8Hex							x
942.307	17.51	7Pent							
666.222	17.58	4Hex				x			
1152.38	17.58	7Hex				x			x
960.317	17.64	5Hex:1Pent							x
1152.38	17.64	7Hex							x
736.232	17.68	4Pent:1GlcAOMe							
942.307	17.78	7Pent							
1314.433	17.86	8Hex							x
798.264	17.94	4Hex:1Pent							
768.253	18.13	3Hex:2Pent							
1314.433	18.17	8Hex				x			
1062.349	18.18	4Hex:3Pent							
942.307	18.29	7Pent							
1074.349	18.42	8Pent							
1314.433	18.54	8Hex							x
768.253	18.65	3Hex:2Pent							
1800.591	18.77	11Hex							
930.306	18.78	4Hex:2Pent							
1314.433	18.79	8Hex							x
678.222	18.79	5Pent							
736.232	18.87	4Pent:1GlcAOMe							
636.211	18.88	3Hex:1Pent			x				
1284.422	18.89	7Hex:1Pent							x
1074.349	18.97	8Pent							
1092.359	18.98	5Hex:2Pent							
930.306	19.2	4Hex:2Pent							
1314.433	19.2	8Hex							x
666.222	19.28	4Hex	x						
1314.433	19.45	8Hex							x
666.222	19.46	4Hex		x	x	x			
1284.422	19.53	7Hex:1Pent							x
1152.38	19.55	7Hex				x			
736.232	19.55	4Pent:1GlcAOMe							
930.306	19.61	4Hex:2Pent							
1074.349	19.65	8Pent							
666.222	19.7	4Hex			x	x			
1284.422	19.75	7Hex:1Pent							x

1206.391	19.75	9Pent						
636.211	19.88	3Hex:1Pent		x				
1092.359	19.94	5Hex:2Pent						
1314.433	19.96	8Hex						x
1962.644	20.05	12Hex						
636.211	20.14	3Hex:1Pent		x				
1314.433	20.18	8Hex			x			
1284.422	20.2	7Hex:1Pent						x
1074.349	20.29	8Pent						
900.296	20.34	3Hex:3Pent						
1314.433	20.38	8Hex						x
666.222	20.41	4Hex		x	x			
1074.349	20.56	8Pent						
1314.433	20.64	8Hex						x
1062.349	20.7	4Hex:3Pent						
1314.433	20.78	8Hex						x
1476.486	20.81	9Hex			x			
2124.697	20.88	13Hex						
1074.349	20.91	8Pent						
868.274	20.93	5Pent:1GlcAOMe						
1062.349	20.95	4Hex:3Pent						
1092.359	21.24	6Hex:1Pent						x
1224.401	21.27	5Hex:3Pent						
810.264	21.35	6Pent						
1338.433	21.37	10Pent						
1206.391	21.45	9Pent						
2286.75	21.47	14Hex						
1314.433	21.54	8Hex						x
1206.391	21.65	9Pent						
1092.359	21.72	6Hex:1Pent						x
798.264	21.76	4Hex:1Pent						
810.264	21.92	6Pent						
1314.433	21.95	8Hex						x
1092.359	22.03	6Hex:1Pent						x
2448.802	22.12	15Hex						
1470.476	22.32	11Pent						
1206.391	22.51	9Pent						
1314.433	22.55	8Hex						x
828.274	22.69	5Hex		x				
1254.412	22.74	6Hex:2Pent						
942.307	22.79	7Pent						
990.327	22.79	6Hex		x				
868.274	22.82	5Pent:1GlcAOMe						
930.306	22.84	4Hex:2Pent						
798.264	22.86	4Hex:1Pent						
2610.855	22.96	16Hex						
868.274	23.13	5Pent:1GlcAOMe						
1092.359	23.14	5Hex:2Pent						
798.264	23.17	4Hex:1Pent						
1206.391	23.29	9Pent						
1314.433	23.36	8Hex						x
868.274	23.46	5Pent:1GlcAOMe						
1638.538	23.51	10Hex			x			
828.274	23.52	5Hex		x				
1602.518	23.54	12Pent						
828.274	23.64	5Hex			x			
1314.433	23.71	8Hex						x
1092.359	23.74	5Hex:2Pent						
1206.391	23.97	9Pent						
798.264	24.02	4Hex:1Pent		x				
798.264	24.43	4Hex:1Pent		x				
1314.433	24.44	8Hex						x
930.306	24.5	4Hex:2Pent						
1000.317	24.51	6Pent:1GlcAOMe						
1092.359	24.78	5Hex:2Pent						
828.274	24.92	5Hex	x	x	x			
1224.401	24.96	5Hex:3Pent						

2772.908	24.97	17Hex							
1734.56	25.02	13Pent							
990.327	25.1	6Hex			x				
930.306	25.19	4Hex:2Pent							
1092.359	25.44	5Hex:2Pent							
798.264	25.49	4Hex:1Pent			x				
942.307	25.73	7Pent							
828.274	25.79	5Hex			x	x			
1224.401	25.81	5Hex:3Pent							
1062.349	26.27	4Hex:3Pent							
798.264	26.37	4Hex:1Pent			x				
828.274	26.44	5Hex	x						
1386.454	26.47	6Hex:3Pent							
1314.433	26.49	8Hex							x
828.274	26.59	5Hex				x			
2934.961	26.68	18Hex							
828.274	26.7	5Hex			x				
1386.454	26.73	6Hex:3Pent							
1000.317	26.88	6Pent:1GlcAOMe							
930.306	27.07	4Hex:2Pent							
1338.433	27.1	10Pent							
1152.38	27.23	7Hex			x				
1386.454	27.33	6Hex:3Pent							
1000.317	27.34	6Pent:1GlcAOMe							
1062.349	27.35	4Hex:3Pent							
1152.38	27.47	7Hex			x				
1132.359	27.61	7Pent:1GlcAOMe							
3097.014	27.67	19Hex							
1074.349	28.15	8Pent							
3259.066	28.55	20Hex							
1152.38	28.61	7Hex			x				
1470.476	28.63	11Pent							
1152.38	28.84	7Hex			x				
990.327	29	6Hex							
3421.119	29.1	21Hex							
1132.359	29.69	7Pent:1GlcAOMe							
990.327	30.9	6Hex			x	x			
990.327	31	6Hex							
990.327	39.26	6Hex			x				
990.327	39.56	6Hex		x					
990.327	40.09	6Hex	x						
990.327	40.8	6Hex							
990.327	41.49	6Hex			x				

Mass (Dal)	RT (min)	Compound	Galactomannan	Arabinan	Xylan	Arabinoxylan	Amylose	Amylopectin	Xyloglucan
504.169	1.51	3Hex	x						
504.169	1.78	3Hex	x						
504.169	1.97	3Hex							
414.137	2	3Pent				x			
474.158	2.01	2Hex:1Pent							
504.169	2.34	3Hex							
504.169	2.56	3Hex	x						
666.222	2.58	4Hex							
474.158	2.69	2Hex:1Pent							
474.158	2.91	2Hex:1Pent							
666.222	2.91	4Hex							
414.137	2.96	3Pent		x					
666.222	3.36	4Hex							
666.222	3.51	4Hex	x						
504.169	3.68	3Hex					x	x	
414.137	3.94	3Pent		x					
666.222	4.01	4Hex	x						
414.137	4.4	3Pent				x			
666.222	4.41	4Hex	x						
666.222	4.51	4Hex							
414.137	4.77	3Pent		x					
666.222	4.94	4Hex	x						
414.137	4.97	3Pent		x					
636.211	5.84	3Hex:1Pen	x						
666.222	5.91	4Hex							
636.211	5.99	3Hex:1Pent							
636.211	6.3	3Hex:1Pen	x						
504.169	6.87	3Hex							
474.158	6.88	2Hex:1Pent							x
636.211	7.05	3Hex:1Pent							
636.211	7.43	3Hex:1Pen	x						
666.222	7.5	4Hex							
474.158	7.68	2Hex:1Pent							x
546.18	8.01	4Pent				x			
666.222	8.18	4Hex							
666.222	8.34	4Hex	x						
504.169	8.71	3Hex							
636.211	8.72	3Hex:1Pent							
828.274	9.15	5Hex							
828.274	9.2	5Hex	x						
666.222	9.32	4Hex							
828.274	9.5	5Hex	x						
636.211	9.52	3Hex:1Pen	x						
1152.38	9.59	7Hex							
990.327	9.72	6Hex							
414.137	9.83	3Pent			x	x			
636.211	9.91	3Hex:1Pen	x						
1152.38	9.99	7Hex							
504.169	10.05	3Hex							
636.211	10.1	3Hex:1Pent							
828.274	10.13	5Hex	x						
546.18	10.19	4Pent				x			

990.327	10.28	6Hex							
1152.38	10.5	7Hex							
828.274	10.54	5Hex	x						
546.18	10.55	4Pent		x					
828.274	10.68	5Hex							
798.264	10.78	4Hex:1Pent							
474.158	10.94	2Hex:1Pent							x
798.264	10.97	4Hex:1Pen	x						
666.222	10.98	4Hex					x	x	
798.264	11.03	4Hex:1Pent							
546.18	11.03	4Pent		x					
828.274	11.06	5Hex	x						
1314.433	11.16	8Hex							
828.274	11.19	5Hex							
546.18	11.2	4Pent				x			
828.274	11.25	5Hex	x						
504.169	11.38	3Hex							
798.264	11.38	4Hex:1Pent							
798.264	11.5	4Hex:1Pen	x						
546.18	11.58	4Pent				x			
546.18	11.66	4Pent		x					
798.264	11.85	4Hex:1Pen	x						
990.327	11.93	6Hex	x						
960.317	12.14	5Hex:1Pent							
798.264	12.15	4Hex:1Pen	x						
546.18	12.16	4Pent				x			
636.211	12.21	3Hex:1Pent							x
990.327	12.23	6Hex	x						
1152.38	12.24	7Hex							
546.18	12.26	4Pent		x					
960.317	12.27	5Hex:1Pent							
474.158	12.39	2Hex:1Pent							
678.222	12.39	5Pent				x			
990.327	12.4	6Hex	x						
990.327	12.46	6Hex							
576.19	12.47	3Pent1Hex							x
798.264	12.5	4Hex:1Pen	x						
636.211	12.51	3Hex:1Pent							
960.317	12.55	5Hex:1Pent							
504.169	12.57	3Hex							
990.327	12.6	6Hex	x						
960.317	12.65	5Hex:1Pen	x						
828.274	12.65	5Hex							
678.222	12.77	5Pent				x			
474.158	12.83	2Hex:1Pent							
828.274	12.85	5Hex					x	x	
990.327	12.86	6Hex	x						
960.317	12.88	5Hex:1Pen	x						
636.211	13.03	3Hex:1Pent							x
960.317	13.04	5Hex:1Pen	x						
604.19	13.06	ent:1GlcAOMe			x				
1152.38	13.1	7Hex							
1152.38	13.11	7Hex	x						
1092.359	13.22	6Hex:1Pent							
960.317	13.23	5Hex:1Pen	x						
828.274	13.47	5Hex							
1152.38	13.51	7Hex	x						
678.222	13.52	5Pent		x					
960.317	13.62	5Hex:1Pen	x						
1152.38	13.65	7Hex							
1314.433	13.68	8Hex							
606.201	13.71	2Hex:2Pent							x
504.169	13.73	3Hex							
828.274	13.73	5Hex							
678.222	13.81	5Pent				x			
1092.359	13.83	6Hex:1Pent							
1092.359	13.84	6Hex:1Pen	x						

990.327	13.85	6Hex					x	x	
1284.422	13.86	7Hex:1Pent							
604.19	14	ent:1GlcAOMe			x				
810.264	14.03	6Pent				x			
828.274	14.03	5Hex							
678.222	14.03	5Pent		x					
1314.433	14.06	8Hex	x						
678.222	14.11	5Pent				x			
666.222	14.13	4Hex							
798.264	14.2	4Hex:1Pent							
474.158	14.21	2Hex:1Pent							
678.222	14.21	5Pent		x					
1092.359	14.24	6Hex:1Pen	x						
604.19	14.27	ent:1GlcAOMe			x				
1314.433	14.34	8Hex	x						
678.222	14.36	5Pent				x			
990.327	14.38	6Hex							
474.158	14.39	2Hex:1Pent							
678.222	14.5	5Pent		x					
1152.38	14.53	7Hex					x	x	
1284.422	14.56	7Hex:1Pen	x						
1476.486	14.62	9Hex							
1314.433	14.67	8Hex	x						
1092.359	14.7	6Hex:1Pen	x						
1476.486	14.75	9Hex							
798.264	14.76	4Hex:1Pent							
828.274	14.81	5Hex							
678.222	14.86	5Pent				x			
1284.422	14.9	7Hex:1Pen	x						
1314.433	14.9	8Hex	x						
1446.475	14.91	8Hex:1Pent							
768.253	14.92	3Hex:2Pent							x
810.264	14.97	6Pent				x			
678.222	14.98	5Pent		x					
1314.433	15.08	8Hex					x	x	
1476.486	15.1	9Hex	x						
810.264	15.11	6Pent		x					
1314.433	15.14	8Hex							
768.253	15.15	3Hex:2Pent							x
504.169	15.17	3Hex							
990.327	15.19	6Hex							
798.264	15.21	4Hex:1Pent							
504.169	15.24	3Hex							
1314.433	15.27	8Hex	x						
546.18	15.33	4Pent			x				
1446.475	15.33	8Hex:1Pent							
828.274	15.34	5Hex							
810.264	15.36	6Pent				x			
1284.422	15.4	7Hex:1Pen	x						
828.274	15.45	5Hex							
1152.38	15.49	7Hex							
960.317	15.56	5Hex:1Pent							
810.264	15.57	6Pent		x					
1314.433	15.58	8Hex	x						
810.264	15.65	6Pent				x			
1152.38	15.65	7Hex							
942.307	15.69	7Pent				x			
636.211	15.72	3Hex:1Pent							x
1152.38	15.72	7Hex							
678.222	15.82	5Pent				x			
828.274	15.85	5Hex							
1476.486	15.97	9Hex	x				x	x	
930.306	16	4Hex:2Pent							x
990.327	16	6Hex							
798.264	16.05	4Hex:1Pent							
1284.422	16.06	7Hex:1Pen	x						
810.264	16.13	6Pent		x		x			

990.327	16.23	6Hex							
828.274	16.32	5Hex							
810.264	16.32	6Pent		x					
1284.422	16.36	7Hex:1Pen	x						
990.327	16.47	6Hex							
604.19	16.47	ent:1GlcAOMe			x				
810.264	16.52	6Pent		x					
636.211	16.55	3Hex:1Pent							
942.307	16.61	7Pent				x			
990.327	16.61	6Hex							
828.274	16.62	5Hex							
636.211	16.66	3Hex:1Pent							x
1314.433	16.7	8Hex							
1314.433	16.72	8Hex							
798.264	16.73	4Hex:1Pent							
678.222	16.76	5Pent				x			
798.264	16.84	4Hex:1Pent							x
636.211	16.85	3Hex:1Pent							
828.274	16.91	5Hex							
1152.38	17.15	7Hex							
678.222	17.15	5Pent				x			
810.264	17.21	6Pent				x			
1152.38	17.24	7Hex							
960.317	17.25	5Hex:1Pent							
1638.538	17.25	10Hex					x	x	
942.307	17.26	7Pent		x					
636.211	17.32	3Hex:1Pent							x
942.307	17.44	7Pent				x			
666.222	17.46	4Hex							
1314.433	17.49	8Hex							
942.307	17.51	7Pent		x					
666.222	17.58	4Hex							
1152.38	17.58	7Hex							
960.317	17.64	5Hex:1Pent							
1152.38	17.64	7Hex							
736.232	17.68	ent:1GlcAOMe			x				
942.307	17.78	7Pent		x					
1314.433	17.86	8Hex							
798.264	17.94	4Hex:1Pent							x
768.253	18.13	3Hex:2Pent							x
1314.433	18.17	8Hex							
1062.349	18.18	4Hex:3Pent							x
942.307	18.29	7Pent				x			
1074.349	18.42	8Pent		x					
1314.433	18.54	8Hex							
768.253	18.65	3Hex:2Pent							x
1800.591	18.77	11Hex					x	x	
930.306	18.78	4Hex:2Pent							x
1314.433	18.79	8Hex							
678.222	18.79	5Pent			x	x			
736.232	18.87	ent:1GlcAOMe			x				
636.211	18.88	3Hex:1Pent							
1284.422	18.89	7Hex:1Pent							
1074.349	18.97	8Pent				x			
1092.359	18.98	5Hex:2Pent							x
930.306	19.2	4Hex:2Pent							x
1314.433	19.2	8Hex							
666.222	19.28	4Hex							
1314.433	19.45	8Hex							
666.222	19.46	4Hex							
1284.422	19.53	7Hex:1Pent							
1152.38	19.55	7Hex							
736.232	19.55	ent:1GlcAOMe			x				
930.306	19.61	4Hex:2Pent							x
1074.349	19.65	8Pent				x			
666.222	19.7	4Hex							
1284.422	19.75	7Hex:1Pent							

1206.391	19.75	9Pent	x					
636.211	19.88	3Hex:1Pent						
1092.359	19.94	5Hex:2Pent						x
1314.433	19.96	8Hex						
1962.644	20.05	12Hex				x	x	
636.211	20.14	3Hex:1Pent						
1314.433	20.18	8Hex						
1284.422	20.2	7Hex:1Pent						
1074.349	20.29	8Pent			x			
900.296	20.34	3Hex:3Pent						x
1314.433	20.38	8Hex						
666.222	20.41	4Hex						
1074.349	20.56	8Pent			x			
1314.433	20.64	8Hex						
1062.349	20.7	4Hex:3Pent						x
1314.433	20.78	8Hex						
1476.486	20.81	9Hex						
2124.697	20.88	13Hex				x	x	
1074.349	20.91	8Pent			x			
868.274	20.93	ent:1GlcAOMe		x				
1062.349	20.95	4Hex:3Pent						x
1092.359	21.24	5Hex:1Pent						
1224.401	21.27	5Hex:3Pent						x
810.264	21.35	6Pent			x			
1338.433	21.37	10Pent	x					
1206.391	21.45	9Pent			x			
2286.75	21.47	14Hex				x	x	
1314.433	21.54	8Hex						
1206.391	21.65	9Pent			x			
1092.359	21.72	5Hex:1Pent						
798.264	21.76	4Hex:1Pent						x
810.264	21.92	6Pent		x	x			
1314.433	21.95	8Hex						
1092.359	22.03	5Hex:1Pent						
2448.802	22.12	15Hex				x	x	
1470.476	22.32	11Pent	x					
1206.391	22.51	9Pent			x			
1314.433	22.55	8Hex						
828.274	22.69	5Hex						
1254.412	22.74	5Hex:2Pent						x
942.307	22.79	7Pent			x			
990.327	22.79	6Hex						
868.274	22.82	ent:1GlcAOMe		x				
930.306	22.84	4Hex:2Pent						x
798.264	22.86	4Hex:1Pent						x
2610.855	22.96	16Hex				x	x	
868.274	23.13	ent:1GlcAOMe		x				
1092.359	23.14	5Hex:2Pent						x
798.264	23.17	4Hex:1Pent						x
1206.391	23.29	9Pent			x			
1314.433	23.36	8Hex						
868.274	23.46	ent:1GlcAOMe		x				
1638.538	23.51	10Hex						
828.274	23.52	5Hex						
1602.518	23.54	12Pent	x					
828.274	23.64	5Hex						
1314.433	23.71	8Hex						
1092.359	23.74	5Hex:2Pent						x
1206.391	23.97	9Pent			x			
798.264	24.02	4Hex:1Pent						
798.264	24.43	4Hex:1Pent						
1314.433	24.44	8Hex						
930.306	24.5	4Hex:2Pent						x
1000.317	24.51	ent:1GlcAOMe		x				
1092.359	24.78	5Hex:2Pent						x
828.274	24.92	5Hex						
1224.401	24.96	5Hex:3Pent						x

2772.908	24.97	17Hex				x	x	
1734.56	25.02	13Pent	x					
990.327	25.1	6Hex						
930.306	25.19	4Hex:2Pent						x
1092.359	25.44	5Hex:2Pent						x
798.264	25.49	4Hex:1Pent						
942.307	25.73	7Pent		x	x			
828.274	25.79	5Hex						
1224.401	25.81	5Hex:3Pent						x
1062.349	26.27	4Hex:3Pent						x
798.264	26.37	4Hex:1Pent						
828.274	26.44	5Hex						
1386.454	26.47	6Hex:3Pent						x
1314.433	26.49	8Hex						
828.274	26.59	5Hex						
2934.961	26.68	18Hex				x	x	
828.274	26.7	5Hex						
1386.454	26.73	6Hex:3Pent						x
1000.317	26.88	ent:1GlcAOMe		x				
930.306	27.07	4Hex:2Pent						x
1338.433	27.1	10Pent			x			
1152.38	27.23	7Hex						
1386.454	27.33	6Hex:3Pent						x
1000.317	27.34	ent:1GlcAOMe		x				
1062.349	27.35	4Hex:3Pent						x
1152.38	27.47	7Hex						
1132.359	27.61	ent:1GlcAOMe		x				
3097.014	27.67	19Hex				x	x	
1074.349	28.15	8Pent		x				
3259.066	28.55	20Hex				x	x	
1152.38	28.61	7Hex						
1470.476	28.63	11Pent			x			
1152.38	28.84	7Hex						
990.327	29	6Hex				x	x	
3421.119	29.1	21Hex				x	x	
1132.359	29.69	ent:1GlcAOMe		x				
990.327	30.9	6Hex						
990.327	31	6Hex				x	x	
990.327	39.26	6Hex						
990.327	39.56	6Hex						
990.327	40.09	6Hex						
990.327	40.8	6Hex	x	x				
990.327	41.49	6Hex						
