# Supplementary material for: A nonenzymatic method for cleaving polysaccharides to yield oligosaccharides for structural analysis
Source: Nat Commun. 2020 Aug 7;11:3963. doi: 10.1038/s41467-020-17778-1 (PMC7414865; doi:10.1038/s41467-020-17778-1)
Supplement: Supplementary file 6 — Supplementary Data 4 [file 41467_2020_17778_MOESM6_ESM.pdf]

Supplementary Data 4

Polysaccharide fingerprinting of wheat and oat bran. Compounds are referred to as their acryonym: Hexose/Hex, Pentose/Pent, O-methylated Glucuronic Acid/GlcAOMe. Polysaccharides are abbreviated: Curdlan/Curd, Cellulose/Cell, β-Glucan/β-Glc, Lichenan/Lich, Galactan/Gal, Mannan/Man, Glucomannan/GlcMan, Galactomannan/GalMan, Arabinan/Ara, Xylan/Xyl, Arabinoxylan/AraXyl, Amylose/Amy, Amylopectin/AmyP, Xyloglucan/XylGlc. When an oligosaccharide can be from multiple polysaccharides, it is denoted in the column which contains multiple polysaccharide names. When an oligosaccharide can only be found in one polysaccharide, it is denoted in the column with only one polysaccharide name. An “X” denotes the presence of the corresponding oligosaccharide from the FITDOG depolymerization of the parent polysaccharide. An “O” denotes an oligosaccharide that was not found in any polysaccharide standard.

Mass (m/z)	RT (Min)	Compound	Arabinan	Xylan	Arabinoxylan	Xylan, Arabinoxylan	β-Glucan	Lichenan
414.14	1	3Pent						
606.2	10.8	2Hex:2Pent						
768.26	12.8	3Hex:2Pent						
546.18	17.2	4Pent						
414.14	2.6	3Pent						
474.16	12.6	2Hex:1Pent						
576.19	10.3	3Pent:1Hex						
636.21	18.4	3Hex:1Pent						
736.23	13.1	4Pent:1GlcAOMe						
736.23	13.4	4Pent:1GlcAOMe						
798.27	16.1	4Hex:1Pent						
798.27	17.4	4Hex:1Pent						
798.27	24.2	4Hex:1Pent						
798.27	24.5	4Hex:1Pent						
504.17	10.1	3Hex						
504.17	15.2	3Hex						x
828.28	23.6	5Hex						x
828.28	26.6	5Hex						x
504.17	11.4	3Hex					x	
666.22	17.5	4Hex					x	
798.27	25.5	4Hex:1Pent					x	
798.27	26.4	4Hex:1Pent					x	
828.28	22.7	5Hex					x	
990.33	39.3	6Hex					x	
414.14	4.4	3Pent			x			
678.22	14.9	5Pent			x			
678.22	15.8	5Pent			x			
678.22	16.8	5Pent			x			
678.22	17.1	5Pent			x			
546.18	15.3	4Pent		x				
604.19	16.5	3Pent:1GlcAOMe		x				
1074.35	28.2	8Pent		x				

414.14	3	3Pent	x	
504.17	3.7	3Hex		
666.22	11	4Hex		
899.29	18.8	11Hex		
980.32	20	12Hex		
990.33	29	5Hex		
990.33	31	5Hex		
990.33	40.8	5Hex		
1061.34	20.9	13Hex		
1142.37	21.5	14Hex		
1223.39	22.1	15Hex		
1304.44	23	16Hex		
1314.44	15.1	8Hex		
1385.44	25	17Hex		
1466.47	26.7	18Hex		
1548.5	27.7	19Hex		
1628.53	28.5	20Hex		
1638.54	17.2	10Hex		
1709.53	29.1	21Hex		
666.22	19.7	4Hex		
666.22	20.4	4Hex		
828.28	25.8	5Hex		
414.14	9.8	3Pent		x
678.22	18.8	5Pent		x
810.27	21.9	6Pent		x
942.31	25.7	7Pent		x
1476.49	16	9Hex		
828.28	12.9	5Hex		
990.33	13.9	6Hex		
1152.38	14.5	7Hex		
504.17	13.7	3Hex		
666.22	19.5	4Hex		
828.28	24.9	5Hex		

Mass (m/z)	RT (Min)	Compound	β-Glcan, Cellulose, Lichenan	β-Glucan, Lichenan	Glucomannan	Amylose	Amylose, Amylopectin, Glucomannan
414.14	1	3Pent					
606.2	10.8	2Hex:2Pent					
768.26	12.8	3Hex:2Pent					
546.18	17.2	4Pent					
414.14	2.6	3Pent					
474.16	12.6	2Hex:1Pent					
576.19	10.3	3Pent:1Hex					
636.21	18.4	3Hex:1Pent					
736.23	13.1	4Pent:1GlcAOMe					
736.23	13.4	4Pent:1GlcAOMe					
798.27	16.1	4Hex:1Pent					
798.27	17.4	4Hex:1Pent					
798.27	24.2	4Hex:1Pent					
798.27	24.5	4Hex:1Pent					
504.17	10.1	3Hex			x		
504.17	15.2	3Hex					
828.28	23.6	5Hex					
828.28	26.6	5Hex					
504.17	11.4	3Hex					
666.22	17.5	4Hex					
798.27	25.5	4Hex:1Pent					
798.27	26.4	4Hex:1Pent					
828.28	22.7	5Hex					
990.33	39.3	6Hex					
414.14	4.4	3Pent					
678.22	14.9	5Pent					
678.22	15.8	5Pent					
678.22	16.8	5Pent					
678.22	17.1	5Pent					
546.18	15.3	4Pent					
604.19	16.5	3Pent:1GlcAOMe					
1074.35	28.2	8Pent					

414.14	3	3Pent				
504.17	3.7	3Hex			x	
666.22	11	4Hex			x	
899.29	18.8	11Hex			x	
980.32	20	12Hex			x	
990.33	29	5Hex			x	
990.33	31	5Hex			x	
990.33	40.8	5Hex			x	
1061.34	20.9	13Hex			x	
1142.37	21.5	14Hex			x	
1223.39	22.1	15Hex			x	
1304.44	23	16Hex			x	
1314.44	15.1	8Hex			x	
1385.44	25	17Hex			x	
1466.47	26.7	18Hex			x	
1548.5	27.7	19Hex			x	
1628.53	28.5	20Hex			x	
1638.54	17.2	10Hex			x	
1709.53	29.1	21Hex			x	
666.22	19.7	4Hex		x		
666.22	20.4	4Hex		x		
828.28	25.8	5Hex		x		
414.14	9.8	3Pent				
678.22	18.8	5Pent				
810.27	21.9	6Pent				
942.31	25.7	7Pent				
1476.49	16	9Hex				x
828.28	12.9	5Hex				x
990.33	13.9	6Hex				x
1152.38	14.5	7Hex				x
504.17	13.7	3Hex	x			
666.22	19.5	4Hex	x			
828.28	24.9	5Hex	x			

Mass (m/z)	RT (Min)	Compound	Wheat Bran	Oat Bran
414.14	1	3Pent	x	x
606.2	10.8	2Hex:2Pent	x	x
768.26	12.8	3Hex:2Pent	x	x
546.18	17.2	4Pent	O	
414.14	2.6	3Pent		O
474.16	12.6	2Hex:1Pent		O
576.19	10.3	3Pent:1Hex		O
636.21	18.4	3Hex:1Pent		O
736.23	13.1	[illegible]Pent:1GlcAOMe		O
736.23	13.4	[illegible]Pent:1GlcAOMe		O
798.27	16.1	4Hex:1Pent		O
798.27	17.4	4Hex:1Pent		O
798.27	24.2	4Hex:1Pent		O
798.27	24.5	4Hex:1Pent		O
504.17	10.1	3Hex		x
504.17	15.2	3Hex	x	x
828.28	23.6	5Hex	x	x
828.28	26.6	5Hex	x	x
504.17	11.4	3Hex	x	x
666.22	17.5	4Hex	x	x
798.27	25.5	4Hex:1Pent	x	x
798.27	26.4	4Hex:1Pent	x	x
828.28	22.7	5Hex	x	x
990.33	39.3	6Hex		x
414.14	4.4	3Pent	x	
678.22	14.9	5Pent	x	
678.22	15.8	5Pent	x	
678.22	16.8	5Pent	x	
678.22	17.1	5Pent	x	
546.18	15.3	4Pent	x	
604.19	16.5	[illegible]Pent:1GlcAOM	x	
1074.35	28.2	8Pent	x	

414.14	3	3Pent	x	x
504.17	3.7	3Hex	x	x
666.22	11	4Hex	x	x
899.29	18.8	11Hex	x	x
980.32	20	12Hex	x	x
990.33	29	5Hex	x	x
990.33	31	5Hex	x	x
990.33	40.8	5Hex	x	x
1061.34	20.9	13Hex	x	x
1142.37	21.5	14Hex	x	x
1223.39	22.1	15Hex	x	x
1304.44	23	16Hex	x	x
1314.44	15.1	8Hex	x	x
1385.44	25	17Hex	x	x
1466.47	26.7	18Hex	x	x
1548.5	27.7	19Hex	x	x
1628.53	28.5	20Hex	x	x
1638.54	17.2	10Hex	x	x
1709.53	29.1	21Hex	x	x
666.22	19.7	4Hex	x	x
666.22	20.4	4Hex	x	x
828.28	25.8	5Hex	x	x
414.14	9.8	3Pent	x	
678.22	18.8	5Pent	x	
810.27	21.9	6Pent	x	
942.31	25.7	7Pent	x	
1476.49	16	9Hex	x	x
828.28	12.9	5Hex	x	x
990.33	13.9	6Hex	x	x
1152.38	14.5	7Hex	x	x
504.17	13.7	3Hex	x	x
666.22	19.5	4Hex	x	x
828.28	24.9	5Hex	x	x
