# Supplementary material for: A nonenzymatic method for cleaving polysaccharides to yield oligosaccharides for structural analysis
Source: Nat Commun. 2020 Aug 7;11:3963. doi: 10.1038/s41467-020-17778-1 (PMC7414865; doi:10.1038/s41467-020-17778-1)
Supplement: Supplementary file 7 — Supplementary Data 5 [file 41467_2020_17778_MOESM7_ESM.pdf]

Supplementary Data 5.

NMR spectra (^{1}H, ^{13}C, COSY, HSQC, HMBC, H2BC) and MS/MS (HPLC-Q-TOF MS) of collected oligosaccharide fractions of galactomannan FITDOG product. The selected chemical shifts (listed below each spectrum), along with monosaccharide and linkage data, were calculated using the CASPER program, where the oligosaccharide structures were predicted (shown before each set of NMR spectra). Oligosaccharides are referred to as the number of their component hexoses – hex – order.

A. 3hex1

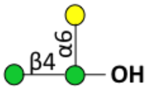

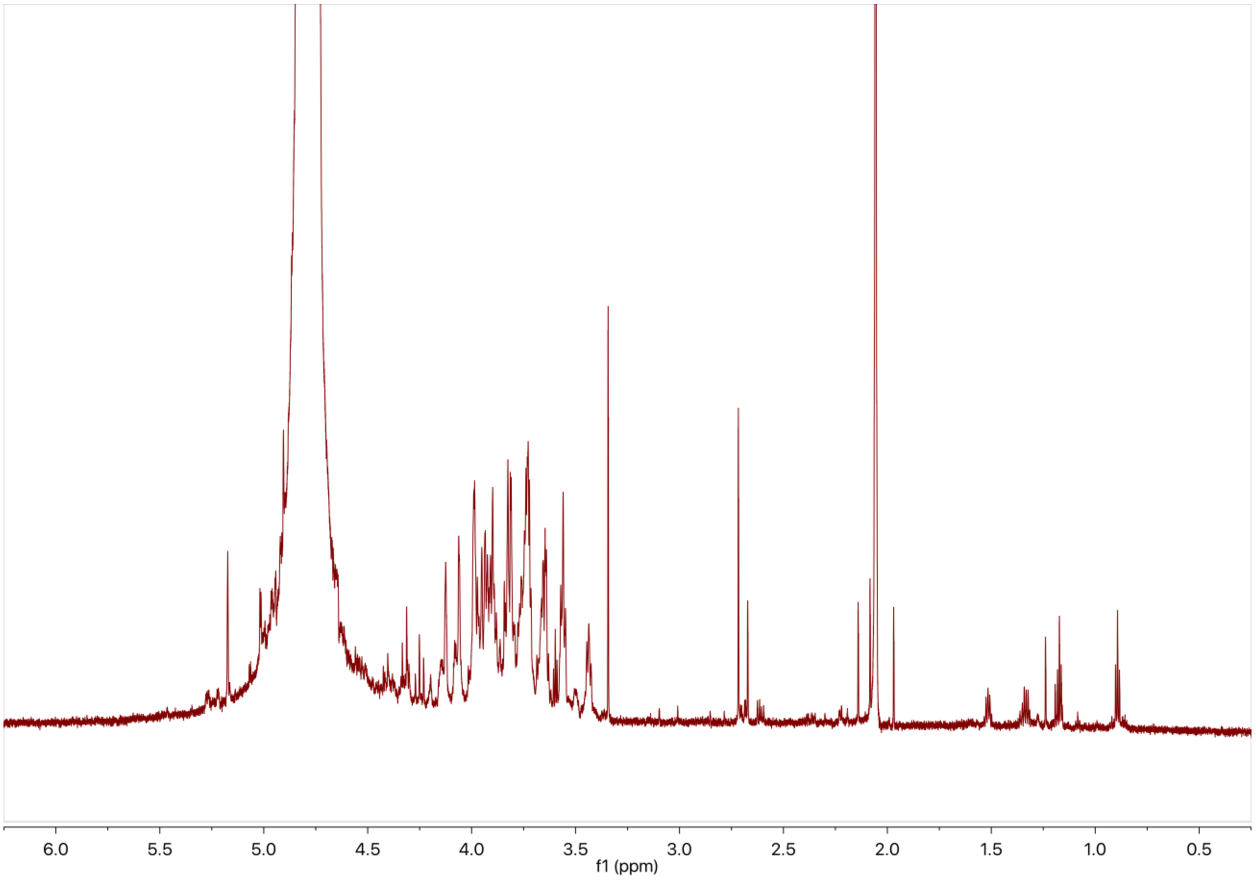

a. **3hex1 ^{1}H NMR** (800 MHz, Deuterium Oxide) δ 5.17, 5.02, 4.90, 4.73, 4.12, 4.06, 3.99, 3.95, 3.94, 3.92, 3.91, 3.90, 3.84, 3.83, 3.81, 3.81, 3.76, 3.74, 3.73, 3.72, 3.66, 3.65, 3.64, 3.60, 3.57, 3.56, 3.55, 3.44.

b. 3hex1 MS/MS

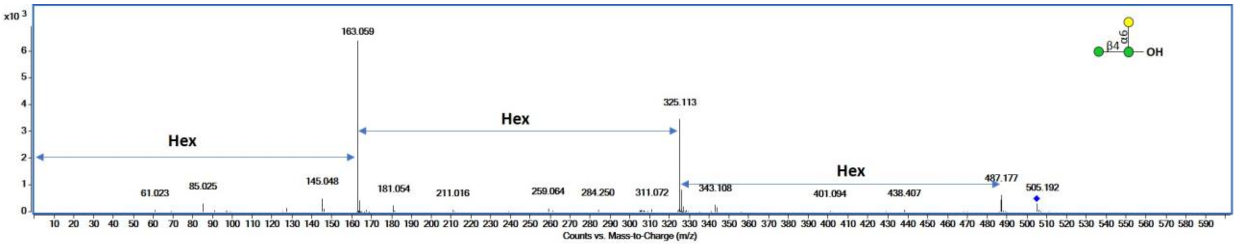

x10 3
163.059
325.113
Hex
Hex
Hex
61.023
85.025
145.048
181.054
211.016
259.064
284.250
311.072
343.108
401.094
438.407
487.177
505.192
β4
α6
OH
Counts vs. Mass-to-Charge (m/z)

B. 4hex1

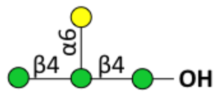

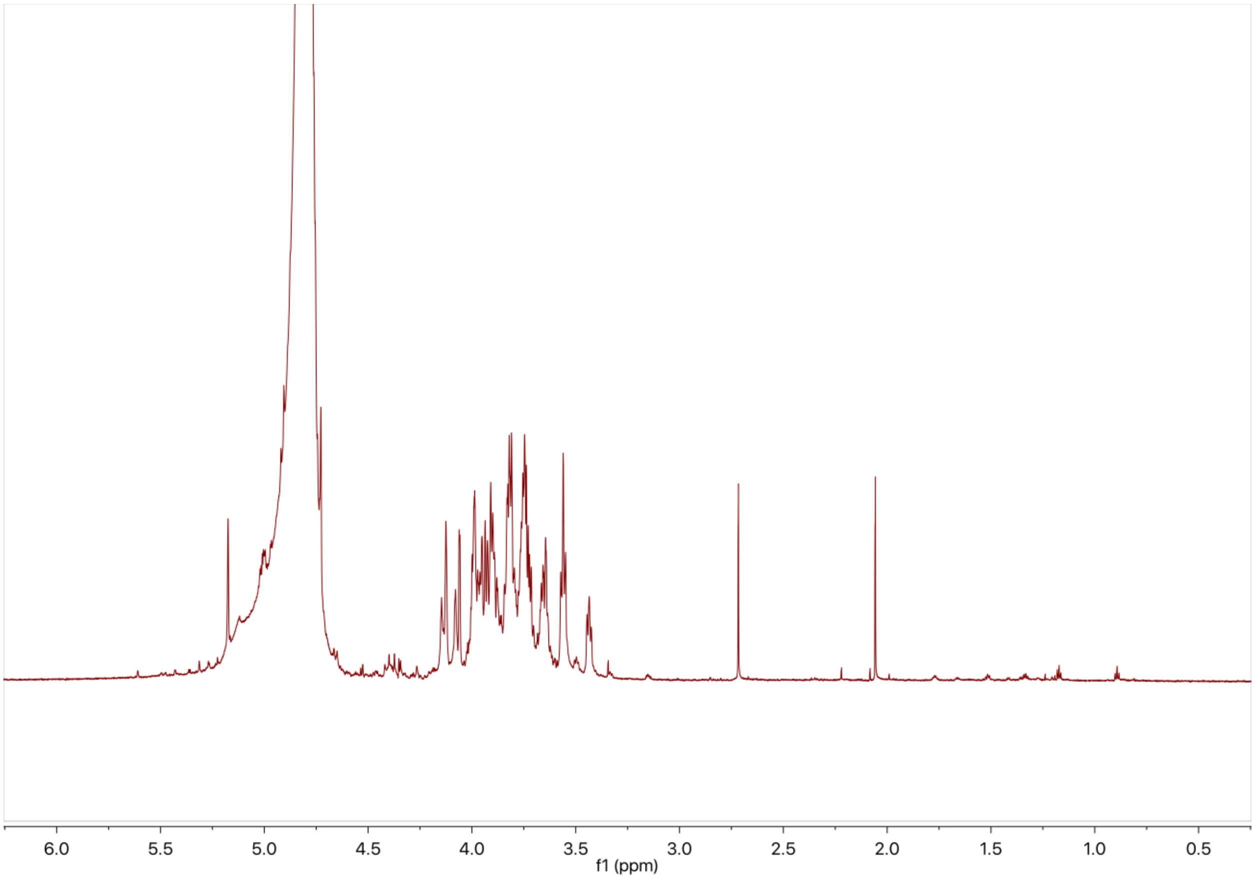

a. 4hex1 ^{1}H NMR (800 MHz, Deuterium Oxide) δ 5.17, 4.92, 4.91, 4.73, 4.15, 4.13, 4.08, 4.06, 4.00, 3.99, 3.97, 3.96, 3.95, 3.94, 3.93, 3.91, 3.90, 3.89, 3.88, 3.83, 3.82, 3.81, 3.80, 3.77, 3.75, 3.75, 3.74, 3.73, 3.72, 3.71, 3.67, 3.66, 3.64, 3.56, 3.43.

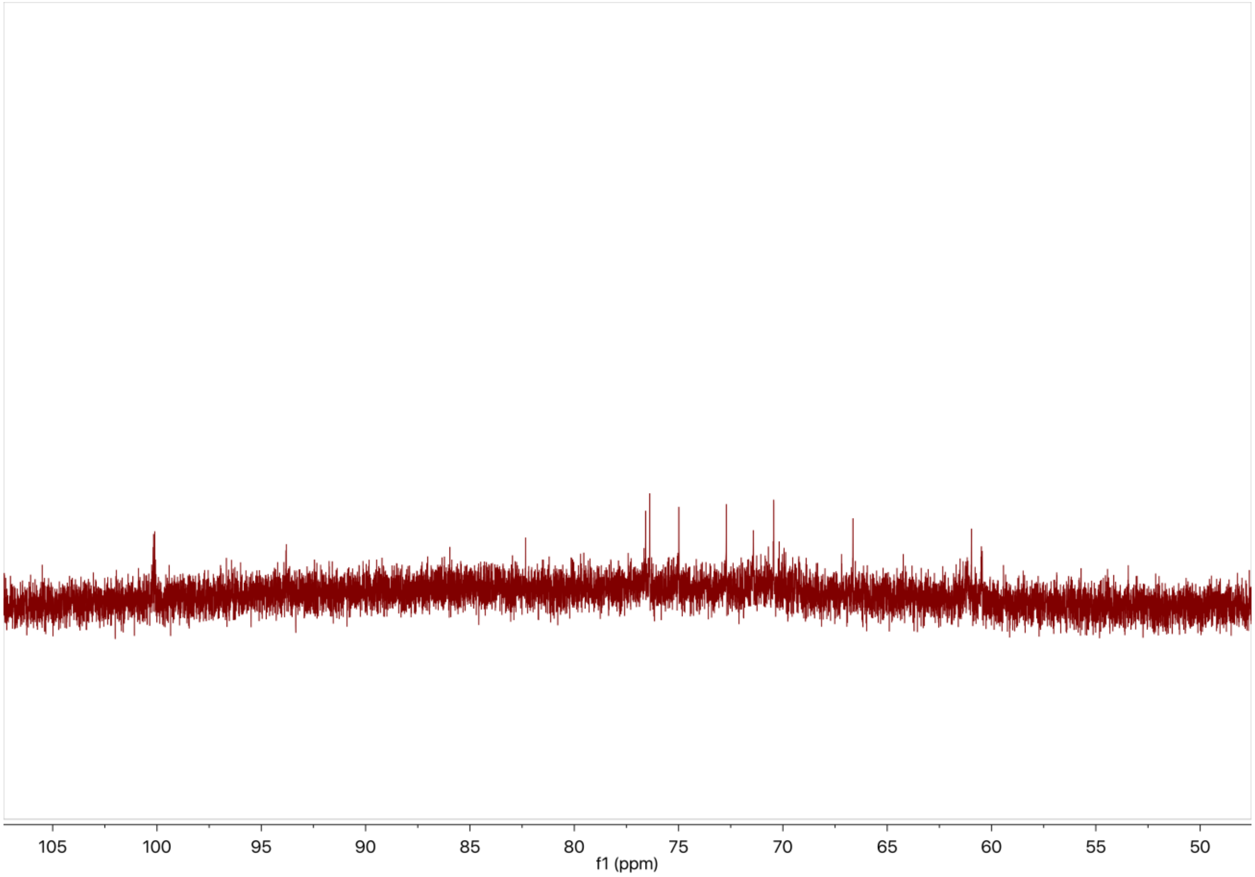

b. 4hex1 ^{13}C NMR (201 MHz, Deuterium Oxide) δ 100.16, 76.58, 76.38, 74.98, 72.71, 71.41, 70.44, 66.64, 60.96, 60.44.

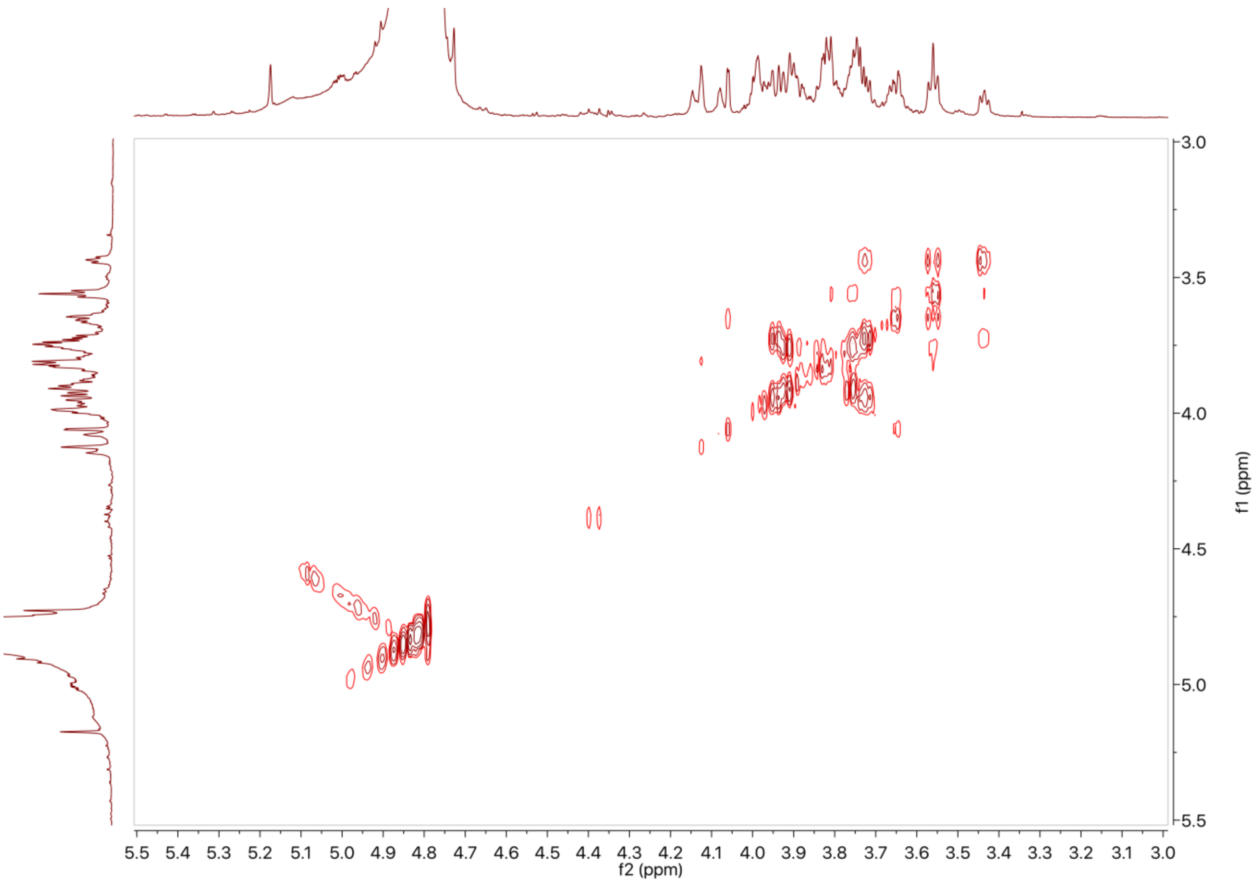

c. 4hex1 COSY

^{1}H NMR (800 MHz, Deuterium Oxide) δ 4.59, 4.61, 4.67, 4.71, 4.76, 4.79, 4.38, 4.38, 3.81, 4.12, 4.08, 4.06, 3.65, 4.00, 3.97, 3.97, 3.73, 3.94, 3.94, 3.76, 3.91, 3.92, 3.76, 3.97, 3.89, 3.75, 3.74, 3.86, 3.84, 3.84, 3.56, 3.79, 3.78, 3.91, 3.56, 3.75, 3.91, 3.56, 3.72, 3.44, 3.94, 3.72, 3.94, 4.00, 3.71, 3.68, 3.68, 3.65, 3.65, 4.06, 3.44, 3.65, 3.56, 3.80, 3.76, 3.56, 3.44, 3.65, 3.44, 3.72, 3.44, 3.56, 4.97

^{1}H NMR (800 MHz, Deuterium Oxide) δ 5.08, 5.07, 5.00, 4.96, 4.92, 4.89, 4.40, 4.37, 4.13, 4.12, 4.08, 4.06, 4.06, 4.00, 3.98, 3.97, 3.95, 3.95, 3.94, 3.93, 3.93, 3.91, 3.91, 3.90, 3.89, 3.89, 3.87, 3.86, 3.84, 3.83, 3.81, 3.80, 3.78, 3.77, 3.76, 3.76, 3.76, 3.75, 3.73, 3.73, 3.73, 3.71, 3.71, 3.70, 3.70, 3.69, 3.67, 3.66, 3.65, 3.64, 3.57, 3.57, 3.56, 3.56, 3.56, 3.55, 3.55, 3.55, 3.45, 3.44, 3.44, 3.44, 3.25.

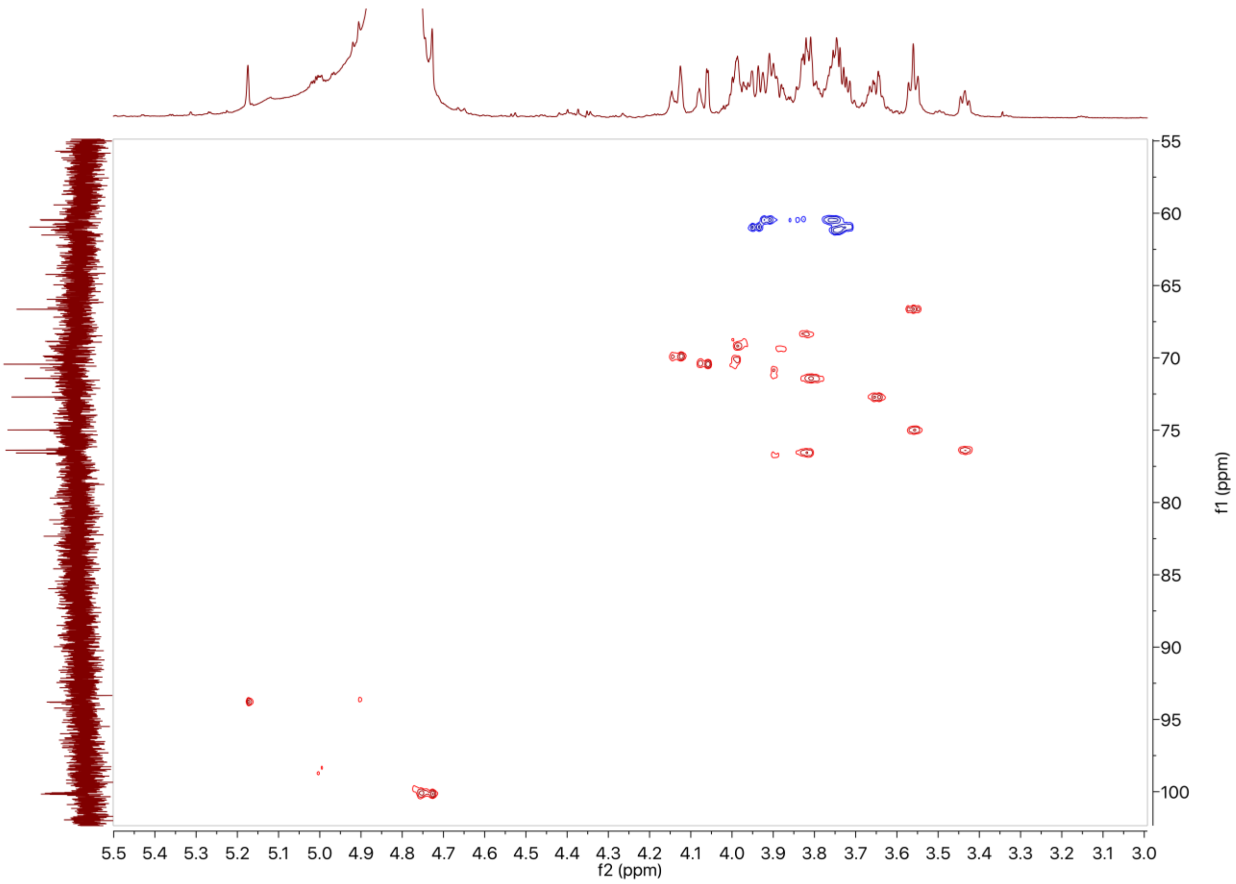

d. 4hex1 HSQC

^{13}C NMR (201 MHz, Deuterium Oxide) δ 93.78, 93.63, 99.79, 100.09, 100.16, 69.89, 70.38, 70.44, 70.15, 69.18, 68.89, 60.96, 60.95, 60.47, 70.85, 69.39, 68.35, 76.56, 71.42, 60.47, 61.15, 72.71, 66.63, 74.99, 76.40

^{1}H NMR (800 MHz, Deuterium Oxide) δ 5.17, 4.90, 4.77, 4.75, 4.73, 4.12, 4.08, 4.06, 3.99, 3.99, 3.97, 3.95, 3.93, 3.91, 3.90, 3.88, 3.82, 3.82, 3.81, 3.76, 3.74, 3.64, 3.56, 3.56, 3.43.

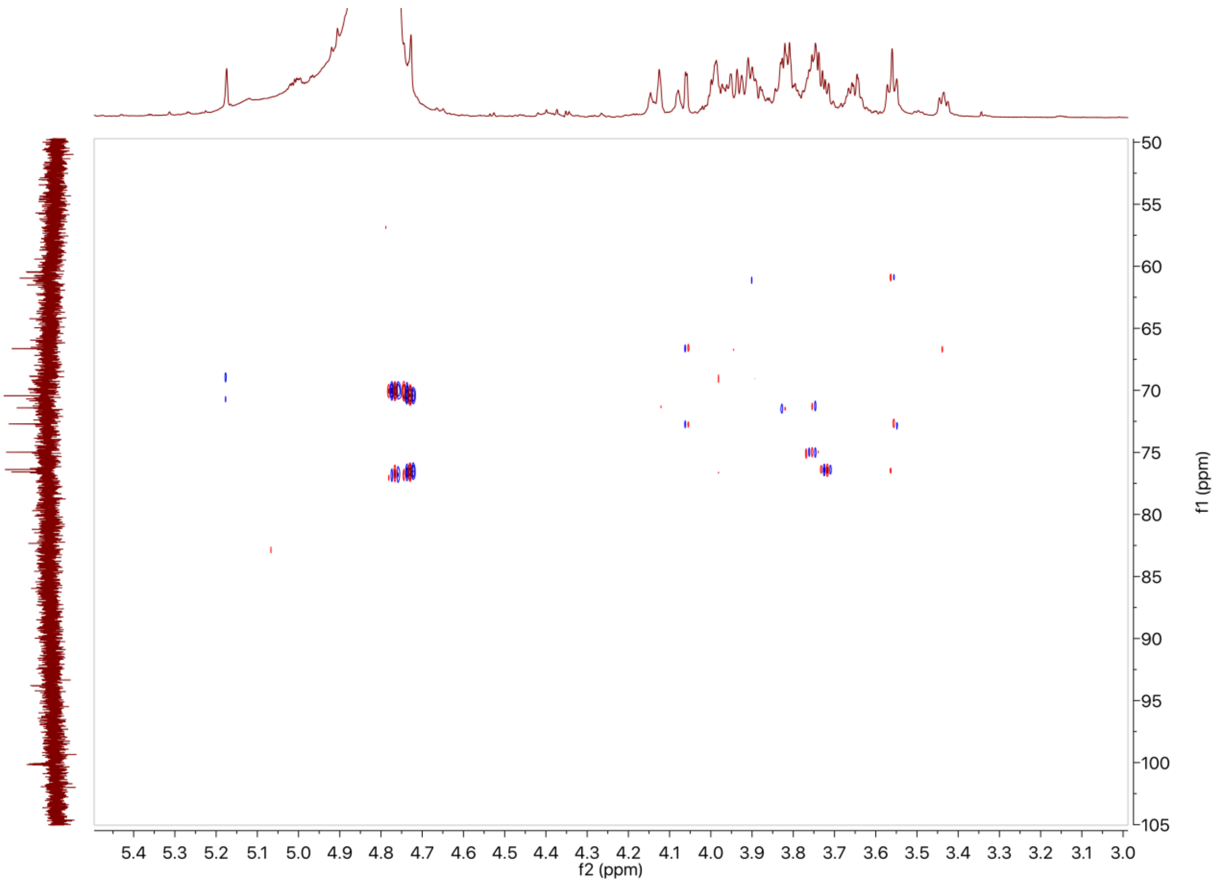

e. 4hex1 HMBC

^{13}C NMR (201 MHz, Deuterium Oxide) δ 70.05, 70.03, 76.73, 70.04, 76.80, 69.99, 70.10, 76.64, 70.35, 76.57, 70.41, 76.52, 70.41, 74.98, 76.42, 76.44, 76.39

^{1}H NMR (800 MHz, Deuterium Oxide) δ 4.78, 4.77, 4.77, 4.77, 4.76, 4.76, 4.75, 4.74, 4.74, 4.73, 4.73, 4.72, 4.72, 3.75, 3.73, 3.72, 3.71.

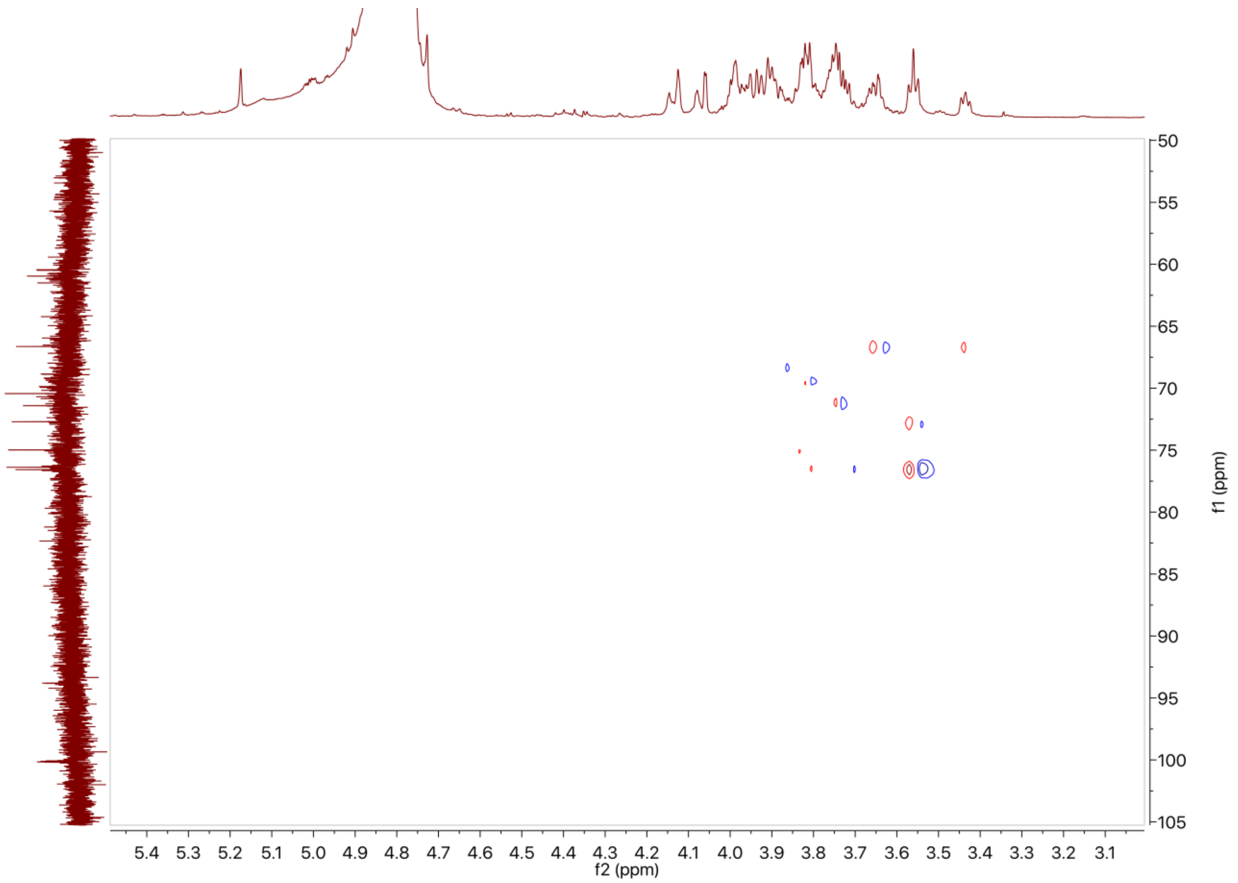

f. 4hex1 H2BC

^{13}C NMR (201 MHz, Deuterium Oxide) δ 69.43, 71.20, 66.69, 66.74, 76.56, 72.83, 76.50

^{1}H NMR (800 MHz, Deuterium Oxide) δ 3.80, 3.73, 3.66, 3.63, 3.57, 3.57, 3.54.

g. 4hex1 MS/MS

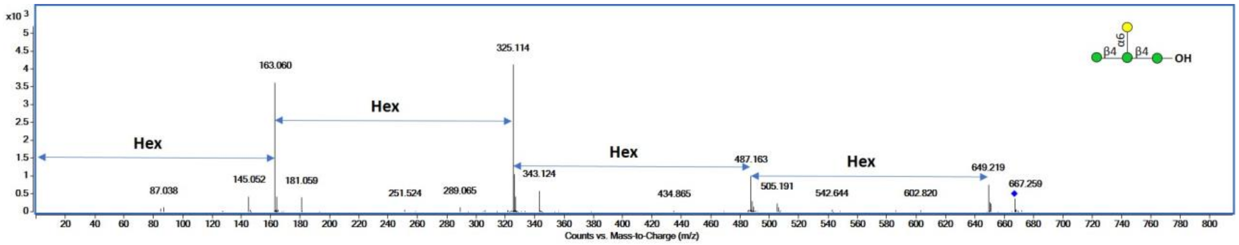
x10 3
163.060
325.114
Hex
Hex
Hex
Hex
487.163
649.219
667.259
87.038
145.052
181.059
251.524
289.065
343.124
434.865
505.191
542.644
602.820
α6
β4
β4
OH
Counts vs. Mass-to-Charge (m/z)

C. 5hex1

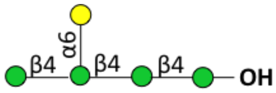

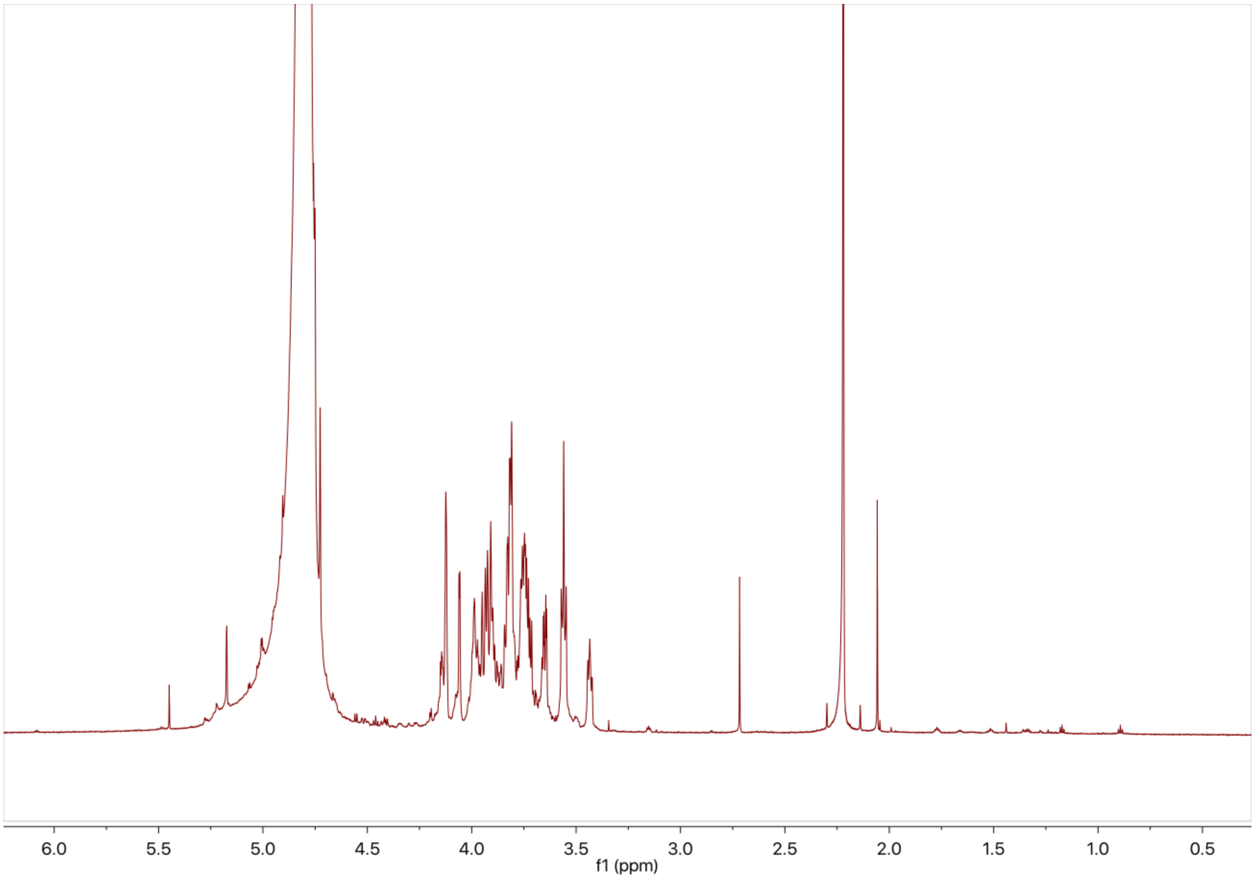

a. 5hex1 ^{1}H NMR (800 MHz, Deuterium Oxide) δ 5.17, 5.17, 4.73, 4.12, 4.06, 4.06, 3.99, 3.99, 3.97, 3.95, 3.93, 3.92, 3.91, 3.90, 3.89, 3.84, 3.83, 3.83, 3.82, 3.82, 3.81, 3.81, 3.80, 3.79, 3.76, 3.76, 3.75, 3.74, 3.74, 3.73, 3.72, 3.71, 3.66, 3.65, 3.65, 3.64, 3.57, 3.56, 3.55, 3.44, 3.43, 3.42.

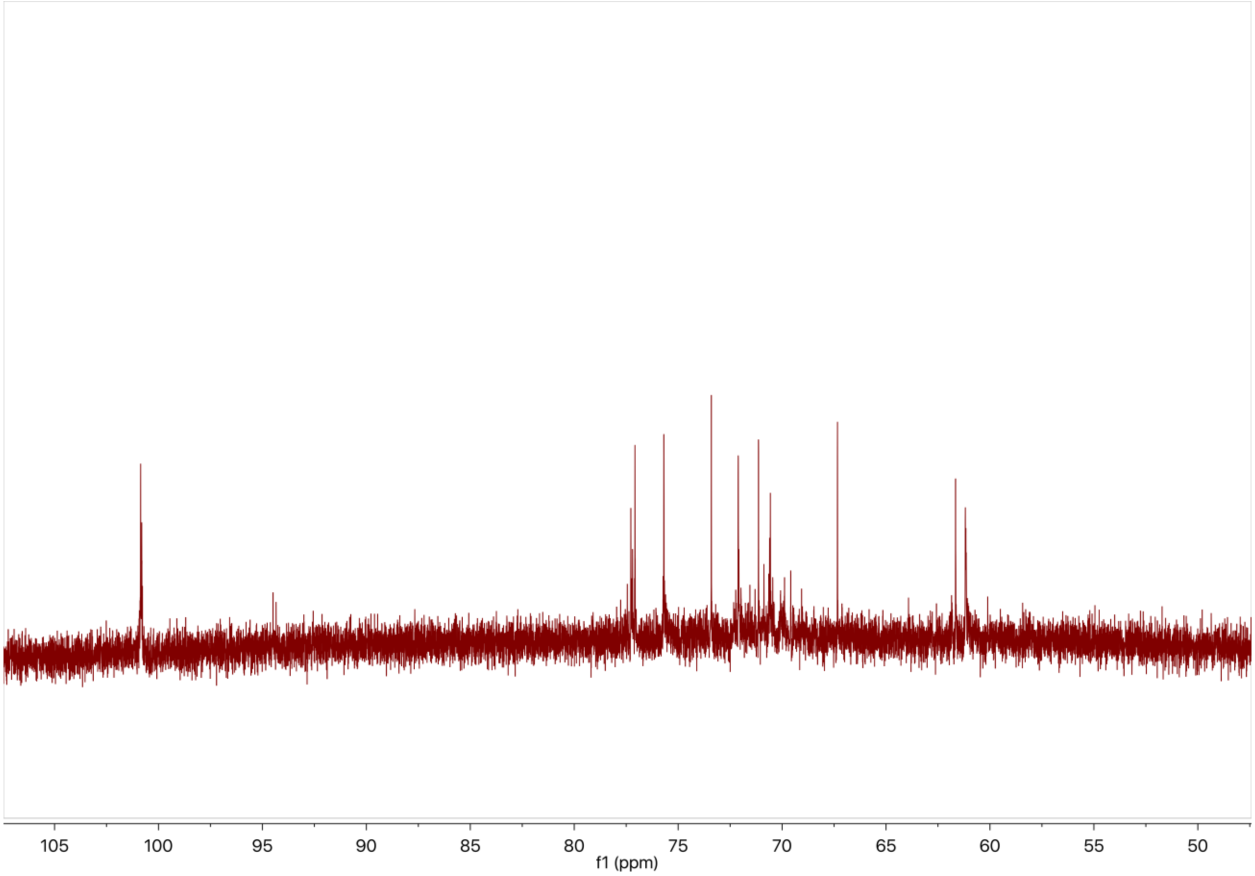

b. 5hex1 ^{13}C NMR (201 MHz, Deuterium Oxide) δ 100.86, 100.81, 94.49, 77.28, 77.20, 77.08, 75.69, 73.40, 72.11, 71.14, 70.87, 70.60, 70.56, 69.58, 67.33, 61.66, 61.18.

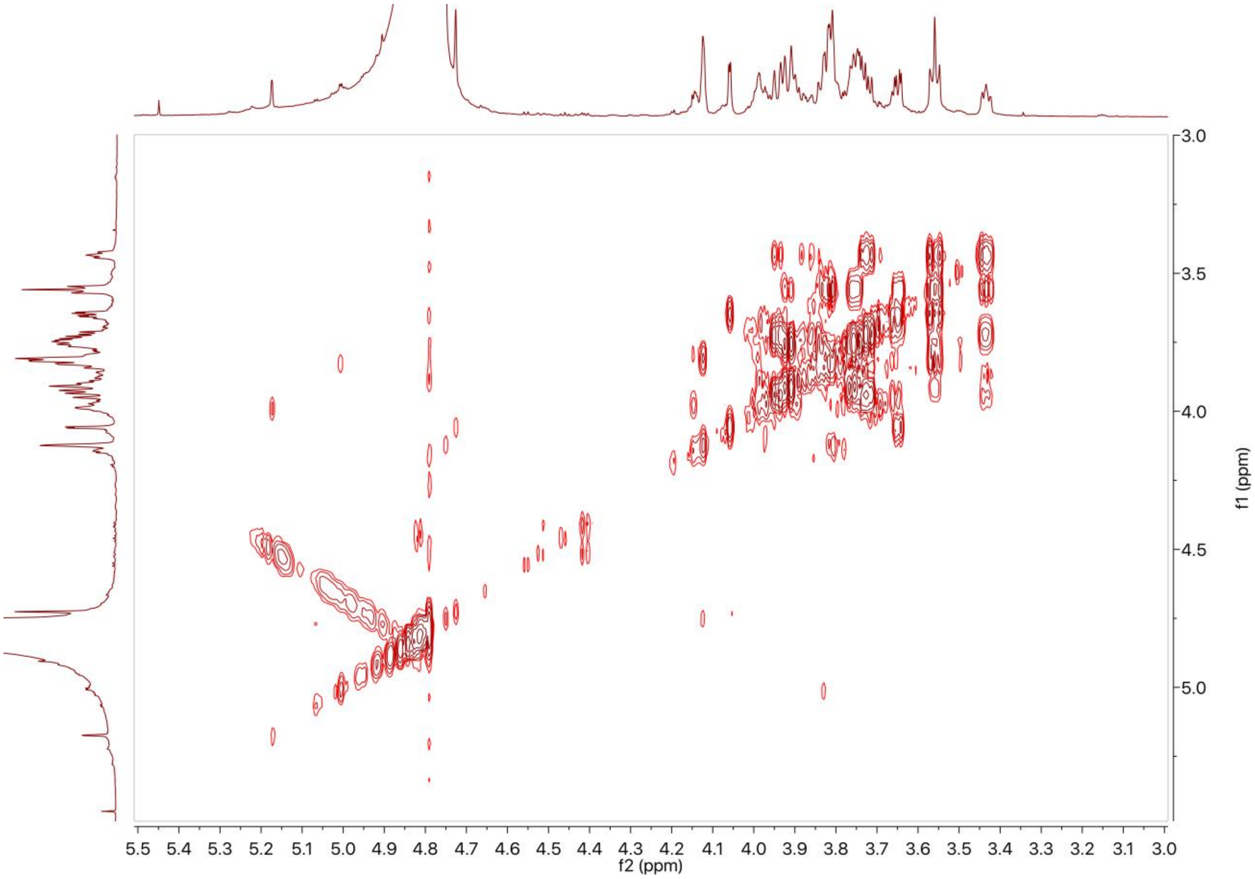

c. 5hex1 COSY

^{1}H NMR (800 MHz, Deuterium Oxide) δ 4.48, 4.50, 3.99, 4.53, 4.64, 4.67, 4.70, 4.74, 4.77, 4.80, 4.46, 4.12, 4.06, 4.56, 4.41, 4.46, 4.51, 4.41, 4.41, 4.14, 4.75, 3.81, 4.12, 3.65, 4.06, 3.70, 4.01, 3.81, 3.89, 3.68, 4.09, 3.74, 3.97, 3.72, 3.94, 3.44, 3.94, 3.72, 3.43, 3.75, 3.55, 3.91, 3.76, 3.91, 3.97, 3.89, 3.74, 3.43, 3.87, 3.87, 3.74, 3.44, 3.72, 3.85, 3.84, 3.56, 3.84, 3.56, 3.83, 3.84, 3.56, 4.13, 3.81, 3.78, 4.14, 3.77, 3.84, 3.91, 3.56, 3.75, 3.91, 3.91, 3.76, 3.72, 3.94, 3.44, 3.94, 3.44, 3.72, 3.68, 3.98, 3.68, 3.67, 3.67, 3.95, 4.06, 3.65, 3.95, 4.06, 3.62, 3.56, 3.44, 3.65, 3.79, 3.56, 3.75, 3.91, 3.65, 3.81, 3.56, 3.81, 3.56, 3.44, 3.65, 3.50, 3.83, 3.56, 3.44, 3.94, 3.72, 3.56, 3.44, 3.87

^{1}H NMR (800 MHz, Deuterium Oxide) δ 5.20, 5.18, 5.17, 5.15, 5.04, 5.01, 4.98, 4.93, 4.90, 4.87, 4.82, 4.75, 4.72, 4.56, 4.51, 4.47, 4.42, 4.42, 4.40, 4.15, 4.12, 4.12, 4.12, 4.06, 4.06, 4.00, 4.00, 3.99, 3.99, 3.98, 3.97, 3.97, 3.97, 3.95, 3.95, 3.95, 3.94, 3.94, 3.93, 3.92, 3.92, 3.92, 3.91, 3.91, 3.90, 3.89, 3.89, 3.88, 3.88, 3.87, 3.86, 3.86, 3.86, 3.86, 3.84, 3.83, 3.83, 3.82, 3.82, 3.81, 3.81, 3.81, 3.80, 3.79, 3.78, 3.78, 3.78, 3.77, 3.76, 3.76, 3.75, 3.74, 3.74, 3.73, 3.73, 3.73, 3.71, 3.71, 3.71, 3.70, 3.69, 3.69, 3.68, 3.66, 3.66, 3.65, 3.65, 3.65, 3.64, 3.62, 3.58, 3.57, 3.57, 3.57, 3.57, 3.56, 3.56, 3.56, 3.56, 3.56, 3.55, 3.55, 3.55, 3.55, 3.50, 3.50, 3.44, 3.44, 3.44, 3.44, 3.43, 3.43, 3.43.

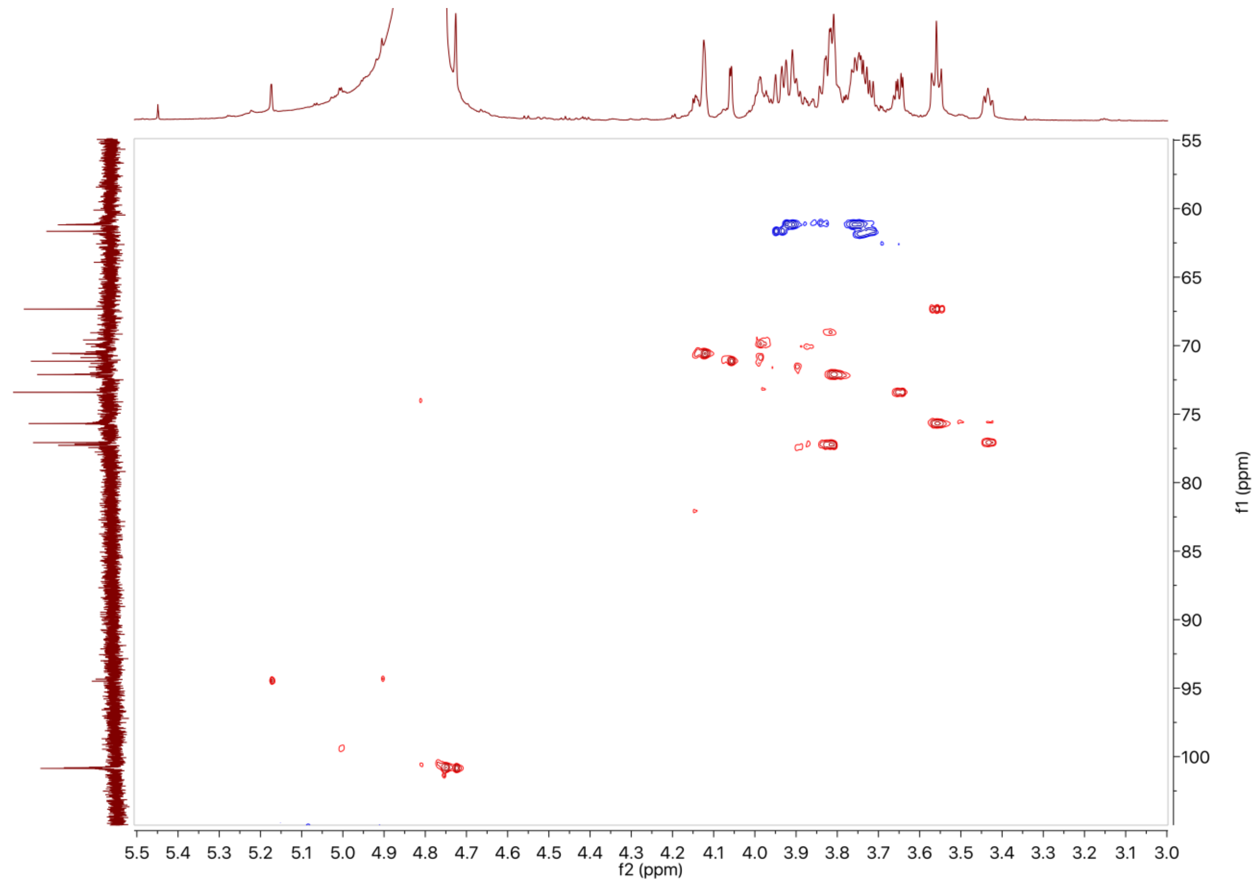

d. 5hex1 HSQC

^{13}C NMR (201 MHz, Deuterium Oxide) δ 94.46, 99.42, 94.31, 100.79, 100.84, 70.58, 71.12, 69.88, 70.86, 61.65, 61.63, 61.17, 61.16, 77.45, 71.53, 70.06, 61.05, 69.01, 77.23, 72.10, 61.16, 61.84, 61.75, 61.69, 62.53, 73.40, 73.40, 67.34, 67.32, 75.68, 67.33, 77.07

^{1}H NMR (800 MHz, Deuterium Oxide) δ 5.17, 5.00, 4.90, 4.75, 4.72, 4.12, 4.06, 3.99, 3.99, 3.95, 3.93, 3.92, 3.91, 3.90, 3.90, 3.87, 3.84, 3.82, 3.82, 3.81, 3.76, 3.74, 3.73, 3.72, 3.69, 3.65, 3.64, 3.57, 3.56, 3.56, 3.55, 3.43.

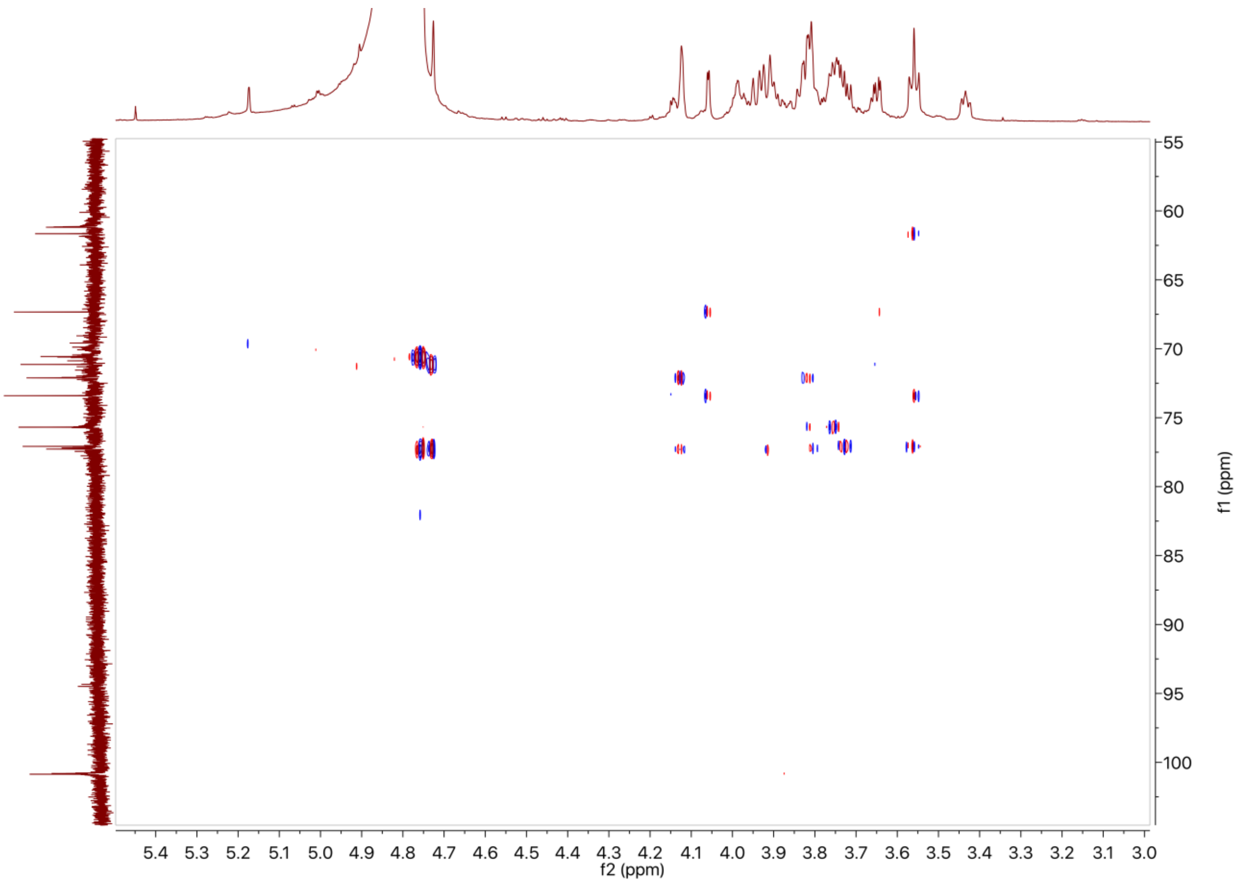

e. 5hex1 HMBC

^{13}C NMR (201 MHz, Deuterium Oxide) δ 69.63, 70.08, 71.27, 70.75, 70.58, 70.62, 70.60, 77.34, 82.04, 70.60, 77.28, 77.22, 70.61, 71.14, 77.24, 71.14, 77.25, 77.24, 71.13, 72.11, 77.26, 72.11, 72.10, 77.27, 72.11, 72.13, 77.31, 67.31, 73.42, 67.29, 73.35, 73.44, 67.36, 77.30, 77.35, 72.09, 72.14, 72.11, 75.64, 72.15, 72.12, 77.23, 77.23, 75.67, 75.69, 75.73, 75.67, 76.99, 75.68, 77.02, 77.18, 77.10, 77.09, 77.07, 71.12, 67.34, 77.13, 61.72, 77.01, 61.64, 77.08, 73.41, 61.67, 77.10, 73.43, 77.09, 61.63, 73.44, 77.09

^{1}H NMR (800 MHz, Deuterium Oxide) δ 5.18, 5.01, 4.91, 4.82, 4.78, 4.78, 4.77, 4.77, 4.76, 4.76, 4.76, 4.75, 4.75, 4.74, 4.74, 4.73, 4.73, 4.72, 4.72, 4.14, 4.13, 4.13, 4.13, 4.12, 4.12, 4.12, 4.12, 4.07, 4.07, 4.06, 4.06, 4.05, 4.05, 3.92, 3.91, 3.83, 3.83, 3.82, 3.82, 3.81, 3.81, 3.80, 3.79, 3.77, 3.76, 3.76, 3.75, 3.74, 3.74, 3.74, 3.74, 3.73, 3.72, 3.71, 3.65, 3.64, 3.58, 3.57, 3.57, 3.56, 3.56, 3.56, 3.56, 3.56, 3.55, 3.55, 3.55, 3.55, 3.54.

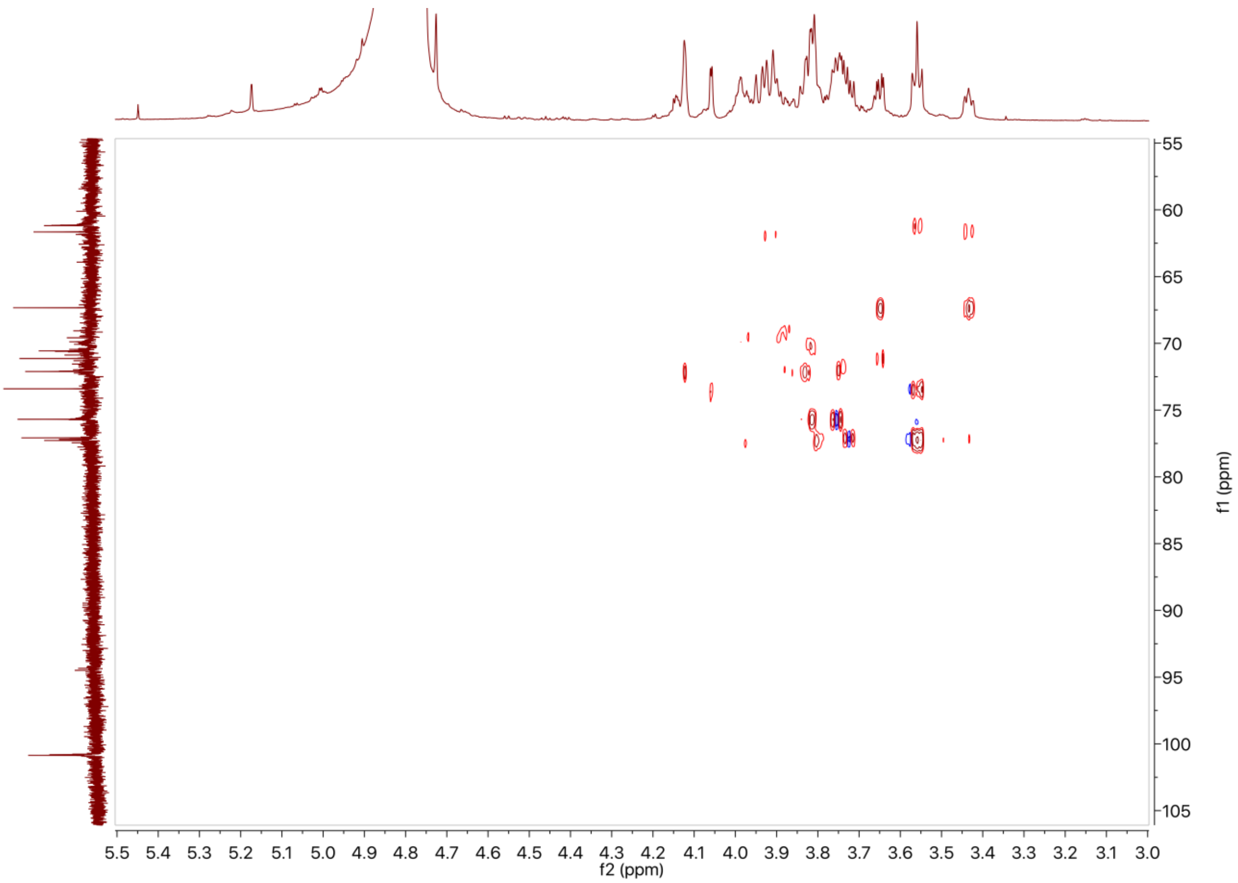

f. 5hex1 H2BC

^{13}C NMR (201 MHz, Deuterium Oxide) δ 72.18, 72.16, 75.73, 77.29, 75.71, 72.03, 77.17, 77.08, 71.17, 67.39, 71.13, 61.22, 77.24, 67.25, 73.46, 67.38

^{1}H NMR (800 MHz, Deuterium Oxide) δ 4.12, 3.83, 3.81, 3.80, 3.75, 3.75, 3.72, 3.72, 3.66, 3.65, 3.64, 3.57, 3.56, 3.56, 3.55, 3.43.

g. 5hex1 MS/MS

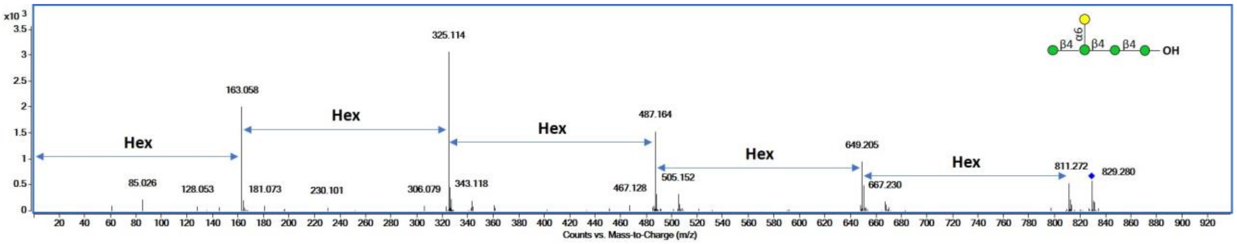

x10 3
325.114
163.058
487.164
649.205
811.272
829.280
85.026
128.053
181.073
230.101
306.079
343.118
467.128
505.152
667.230
Hex
Hex
Hex
Hex
Hex
β4
α6
β4
β4
OH
Counts vs. Mass-to-Charge (m/z)

D. 6hex1

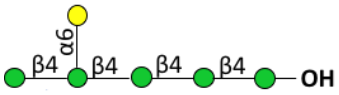

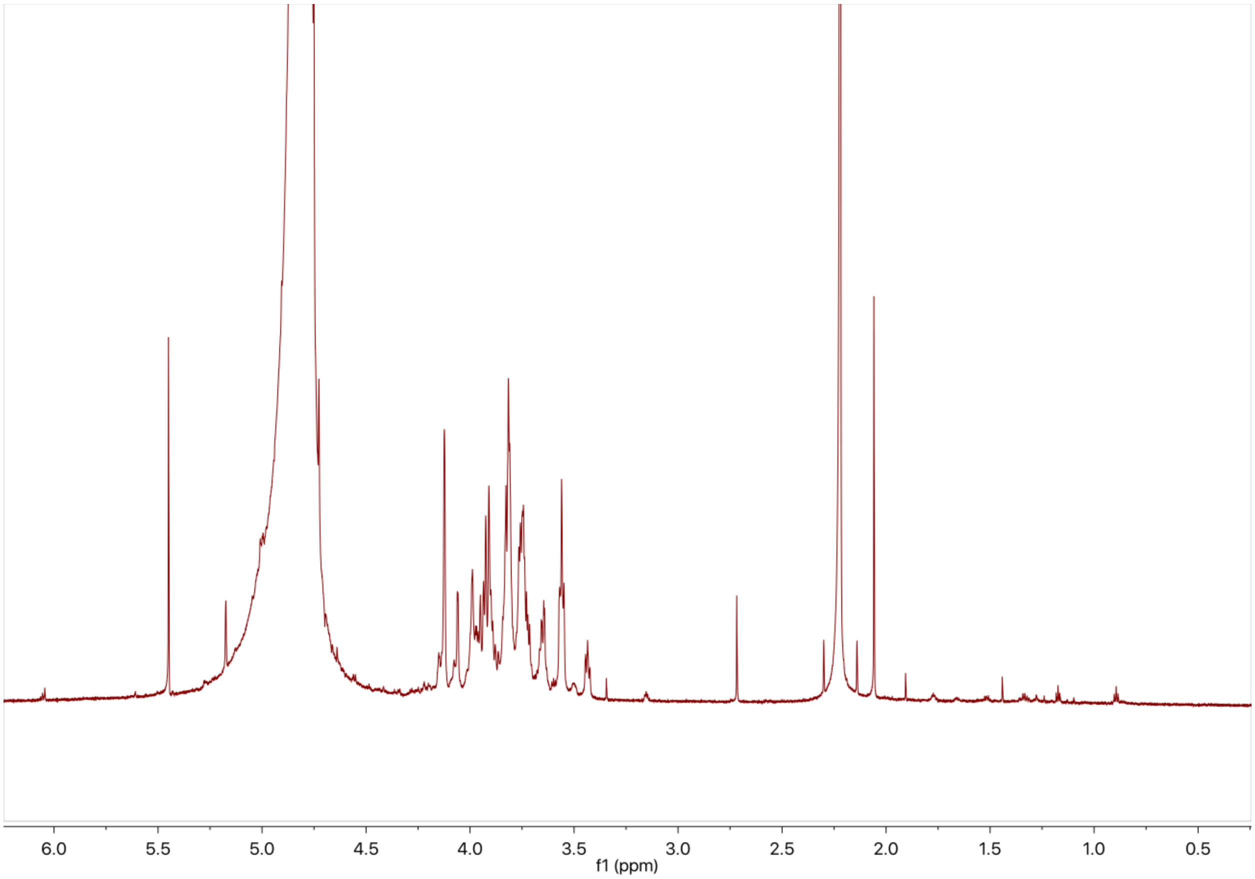

a. 6hex1 ^{1}H NMR (800 MHz, Deuterium Oxide) δ 5.17, 4.73, 4.12, 4.06, 4.06, 4.00, 3.99, 3.99, 3.97, 3.97, 3.96, 3.95, 3.94, 3.92, 3.91, 3.90, 3.89, 3.88, 3.86, 3.84, 3.83, 3.83, 3.82, 3.82, 3.81, 3.81, 3.79, 3.77, 3.76, 3.75, 3.74, 3.74, 3.73, 3.72, 3.71, 3.66, 3.65, 3.65, 3.64, 3.57, 3.57, 3.56, 3.55, 3.44, 3.43, 3.42.

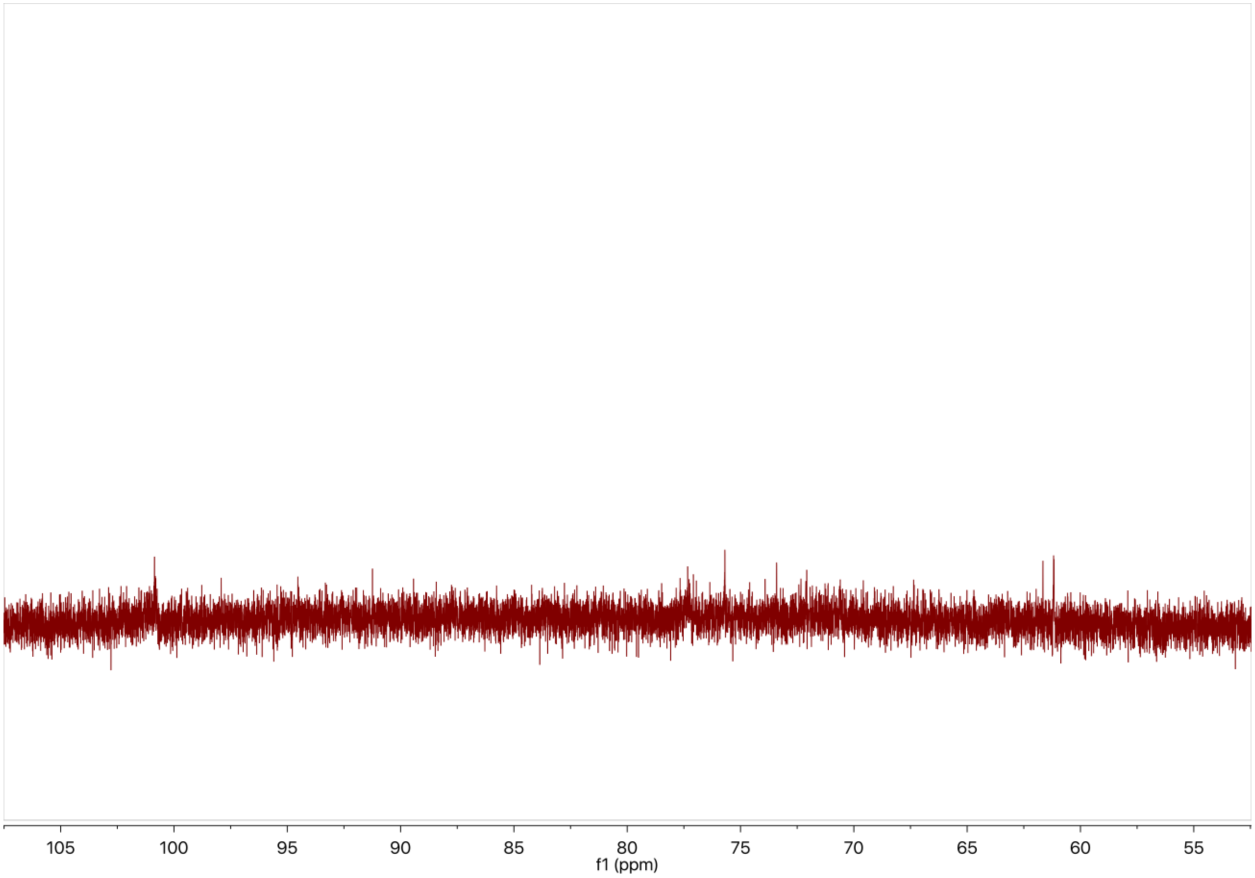

b. 6hex1 ^{13}C NMR (201 MHz, Deuterium Oxide) δ 100.86, 75.69, 73.41, 61.66, 61.18.

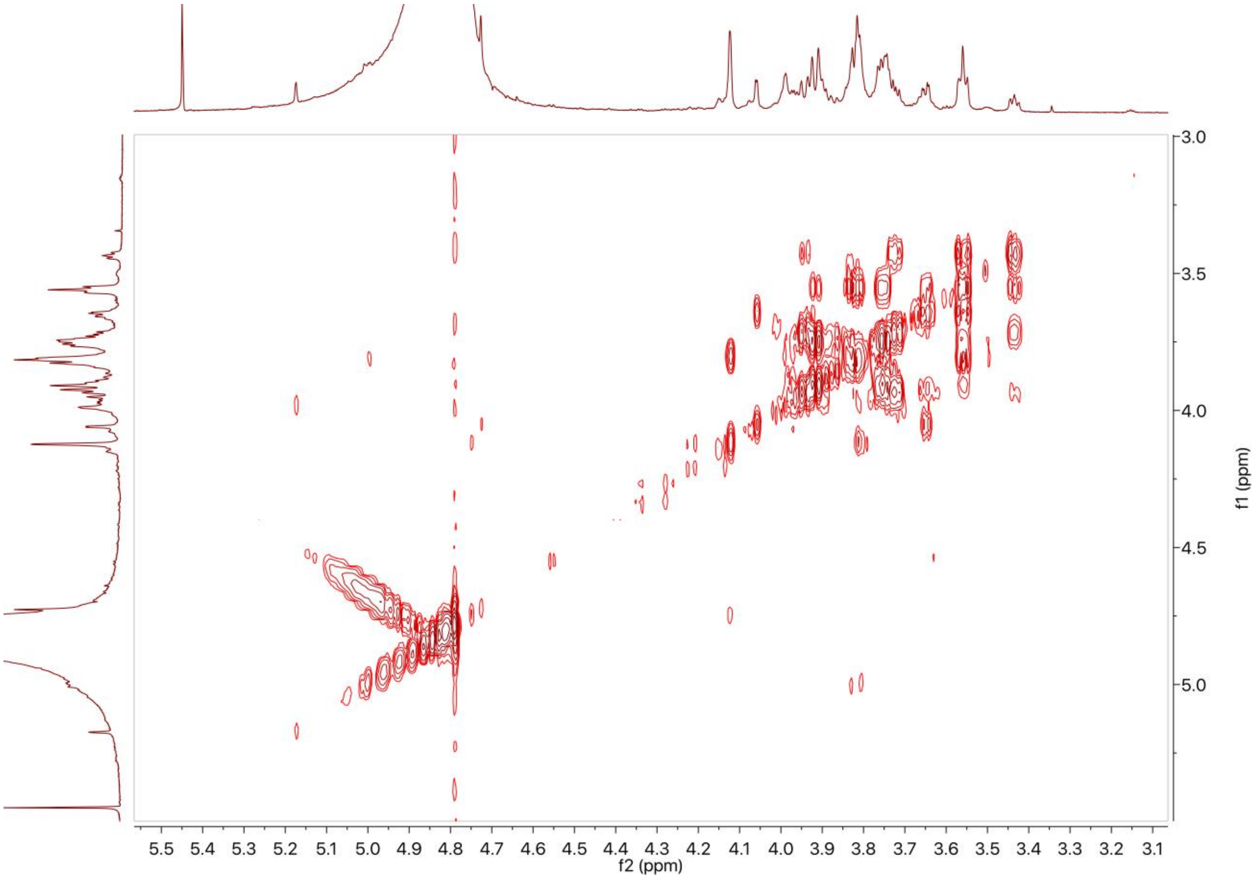

c. 6hex1 COSY

^{1}H NMR (800 MHz, Deuterium Oxide) δ 3.98, 5.17, 4.70, 4.96, 4.73, 4.74, 4.92, 4.77, 4.89, 4.78, 4.79, 4.86, 4.84, 4.82, 4.80, 4.78, 4.74, 4.11, 4.04, 4.75, 4.11, 3.80, 3.64, 4.05, 3.96, 3.72, 3.93, 3.72, 3.94, 3.75, 3.91, 3.55, 3.75, 3.55, 3.91, 3.88, 3.86, 3.74, 3.86, 3.55, 3.76, 3.83, 3.55, 3.82, 3.55, 4.11, 3.81, 3.55, 3.76, 3.74, 3.55, 3.91, 3.91, 3.75, 3.71, 3.93, 3.43, 3.93, 3.72, 3.66, 3.66, 3.64, 3.64, 4.05, 3.56, 3.43, 3.64, 3.80, 3.55, 3.74, 3.80, 3.55, 3.55, 3.80, 3.43, 3.64, 3.49, 3.43, 3.71, 3.43

^{1}H NMR (800 MHz, Deuterium Oxide) δ 5.17, 5.17, 4.97, 4.96, 4.95, 4.93, 4.92, 4.90, 4.89, 4.89, 4.87, 4.86, 4.84, 4.83, 4.81, 4.79, 4.75, 4.75, 4.72, 4.12, 4.12, 4.12, 4.06, 4.06, 3.97, 3.95, 3.95, 3.93, 3.93, 3.92, 3.92, 3.92, 3.91, 3.91, 3.91, 3.89, 3.88, 3.86, 3.86, 3.84, 3.83, 3.83, 3.82, 3.82, 3.81, 3.81, 3.81, 3.81, 3.78, 3.76, 3.76, 3.75, 3.74, 3.74, 3.73, 3.73, 3.72, 3.71, 3.71, 3.68, 3.67, 3.66, 3.64, 3.64, 3.64, 3.57, 3.57, 3.57, 3.57, 3.56, 3.56, 3.56, 3.55, 3.55, 3.55, 3.55, 3.50, 3.44, 3.44, 3.43.

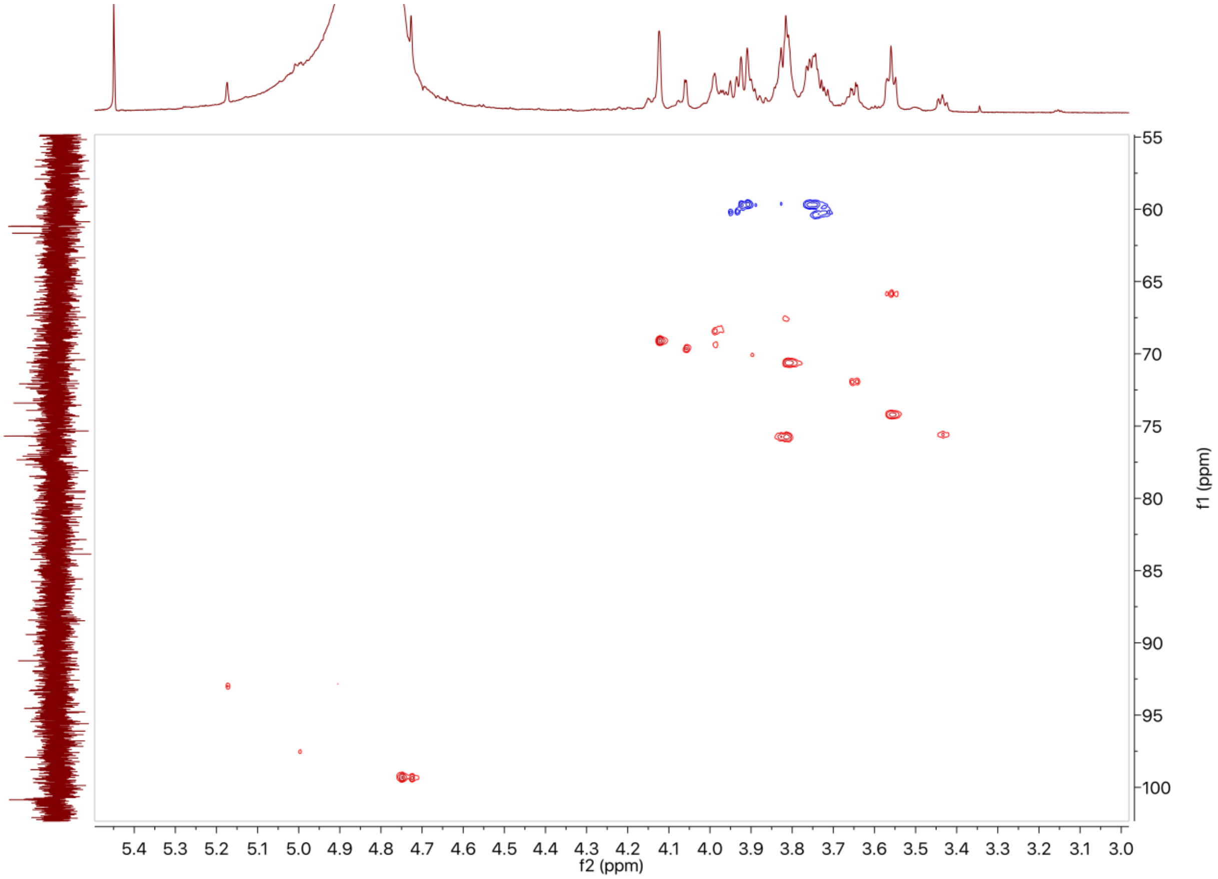

d. 6hex1 HSQC

^{13}C NMR (201 MHz, Deuterium Oxide) δ 93.00, 97.52, 99.31, 99.35, 69.10, 69.65, 68.44, 60.15, 59.71, 59.67, 70.06, 75.73, 70.62, 59.69, 60.33, 71.96, 71.93, 65.84, 74.21, 75.60

^{1}H NMR (800 MHz, Deuterium Oxide) δ 5.17, 5.00, 4.75, 4.72, 4.12, 4.06, 3.99, 3.93, 3.92, 3.91, 3.90, 3.81, 3.81, 3.76, 3.74, 3.65, 3.64, 3.56, 3.56, 3.43.

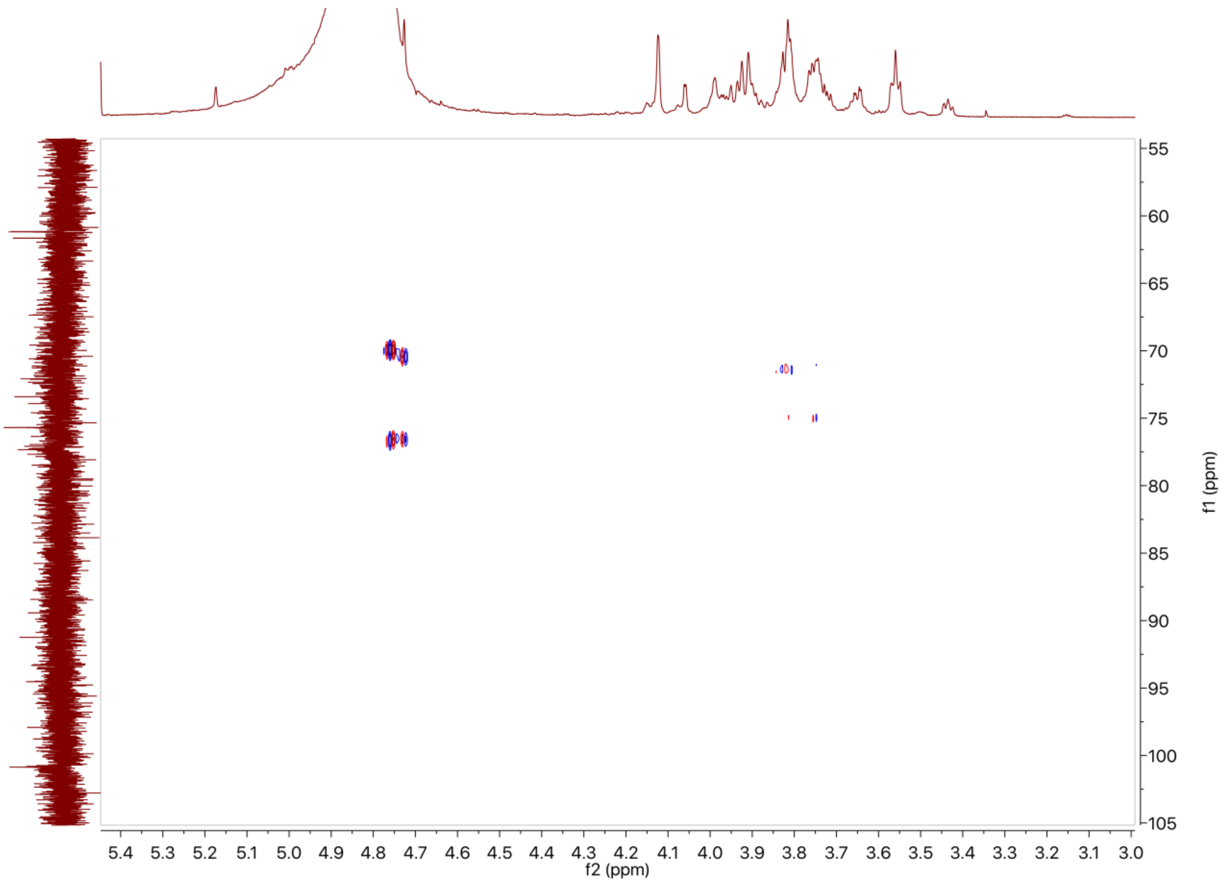

e. 6hex1 HMBC

^{13}C NMR (201 MHz, Deuterium Oxide) δ 69.93, 76.63, 69.91, 76.55, 69.92, 76.50, 76.54, 70.43, 70.45, 76.56, 71.36, 71.34, 71.42, 74.97

^{1}H NMR (800 MHz, Deuterium Oxide) δ 4.77, 4.76, 4.76, 4.75, 4.75, 4.74, 4.73, 4.73, 4.72, 4.72, 3.83, 3.82, 3.81, 3.75.

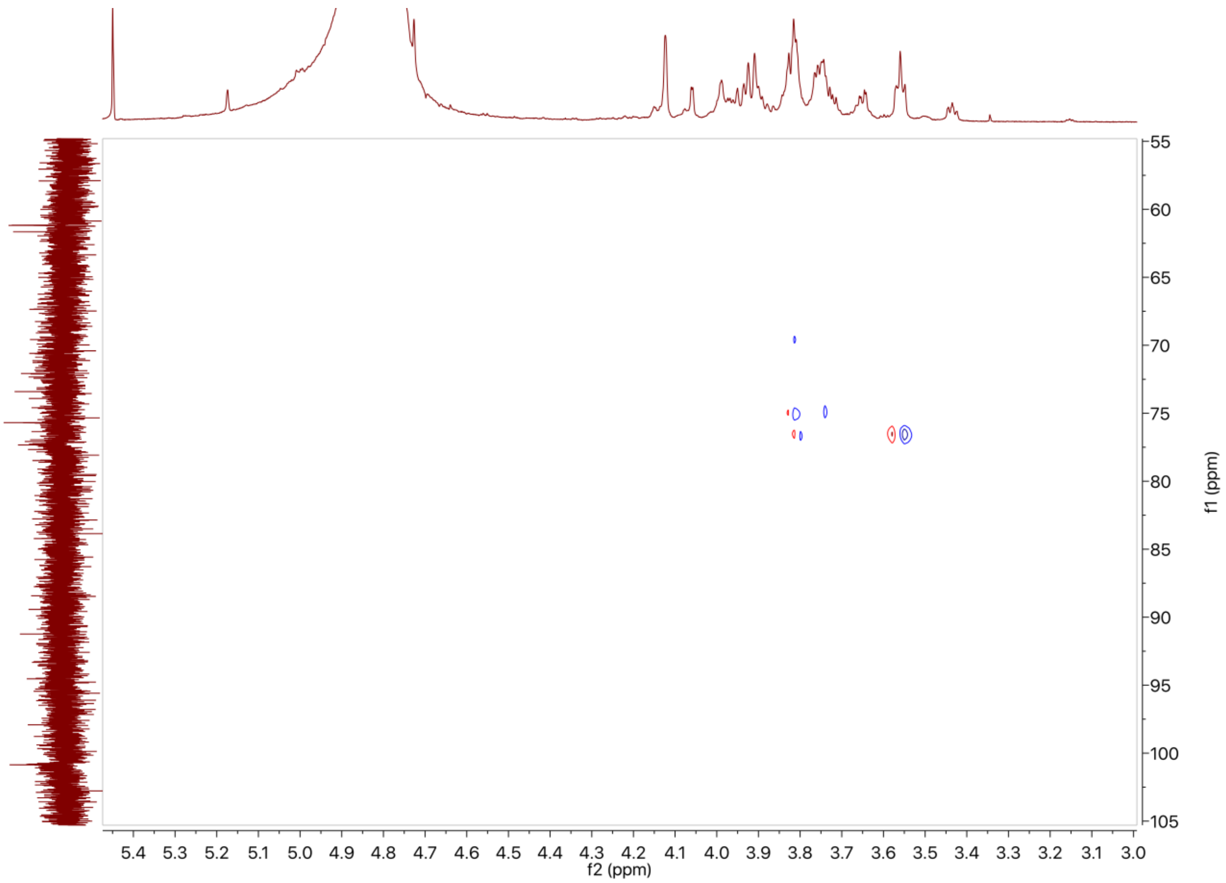

f. 6hex1 H2BC

^{13}C NMR (201 MHz, Deuterium Oxide) δ 75.08, 76.53, 76.56

^{1}H NMR (800 MHz, Deuterium Oxide) δ 3.81, 3.58, 3.55.

g. 6hex1 MS/MS

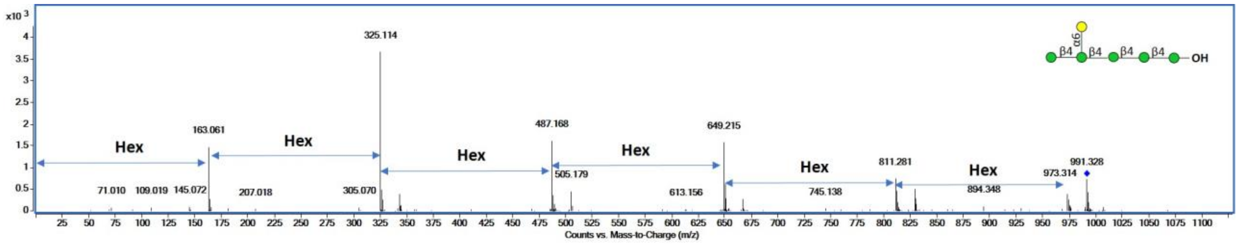
x10 3
Hex
Hex
Hex
Hex
Hex
Hex
325.114
163.061
487.168
649.215
811.281
991.328
973.314
505.179
71.010
109.019
145.072
207.018
305.070
613.156
745.138
894.348
α6
β4
β4
β4
β4
OH
Counts vs. Mass-to-Charge (m/z)

E. 5hex2

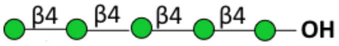

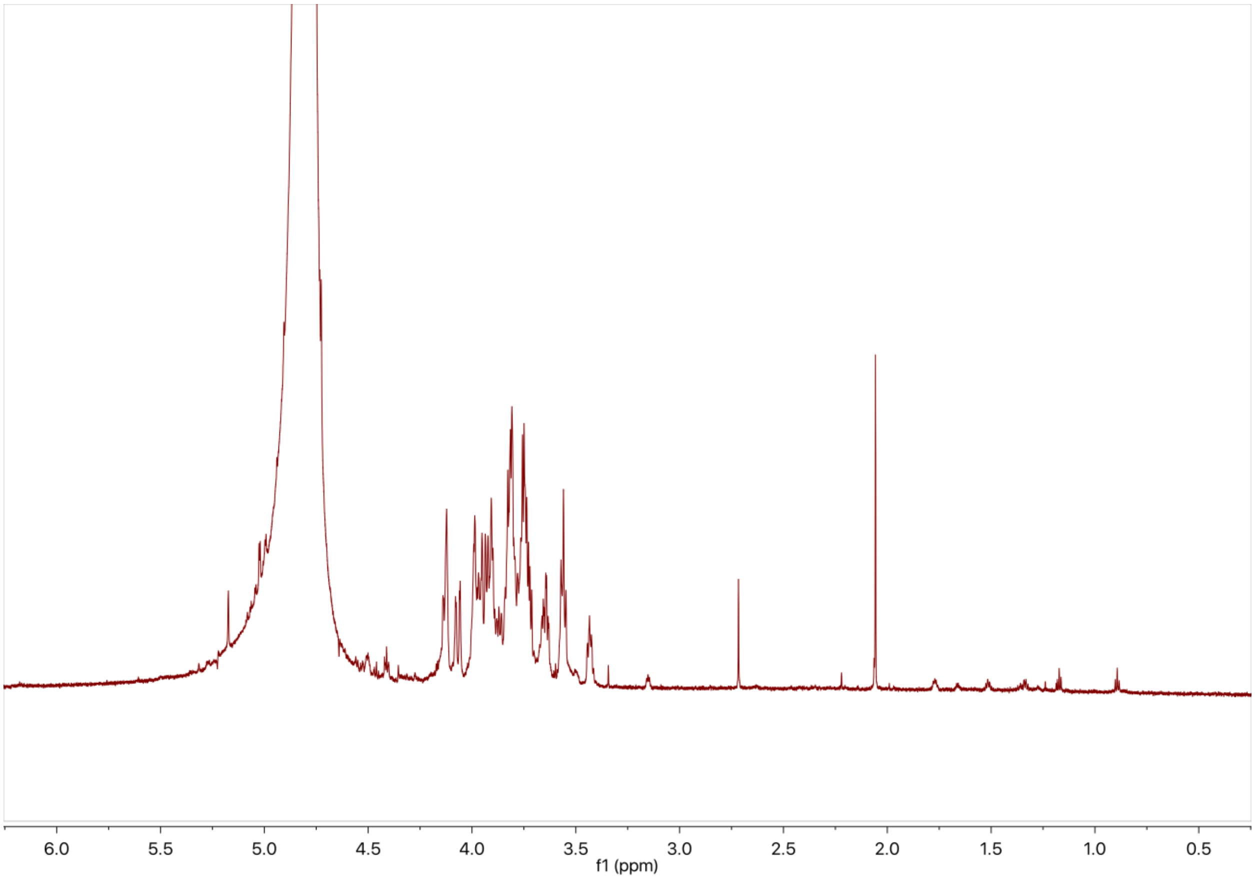

a. 5hex2 ^{1}H NMR (800 MHz, Deuterium Oxide) δ 5.17, 4.73, 4.14, 4.12, 4.08, 4.06, 3.99, 3.99, 3.98, 3.97, 3.96, 3.95, 3.94, 3.92, 3.91, 3.90, 3.89, 3.88, 3.87, 3.86, 3.84, 3.83, 3.82, 3.81, 3.78, 3.76, 3.76, 3.75, 3.74, 3.74, 3.73, 3.72, 3.71, 3.66, 3.66, 3.65, 3.64, 3.63, 3.63, 3.56, 3.55, 3.43.

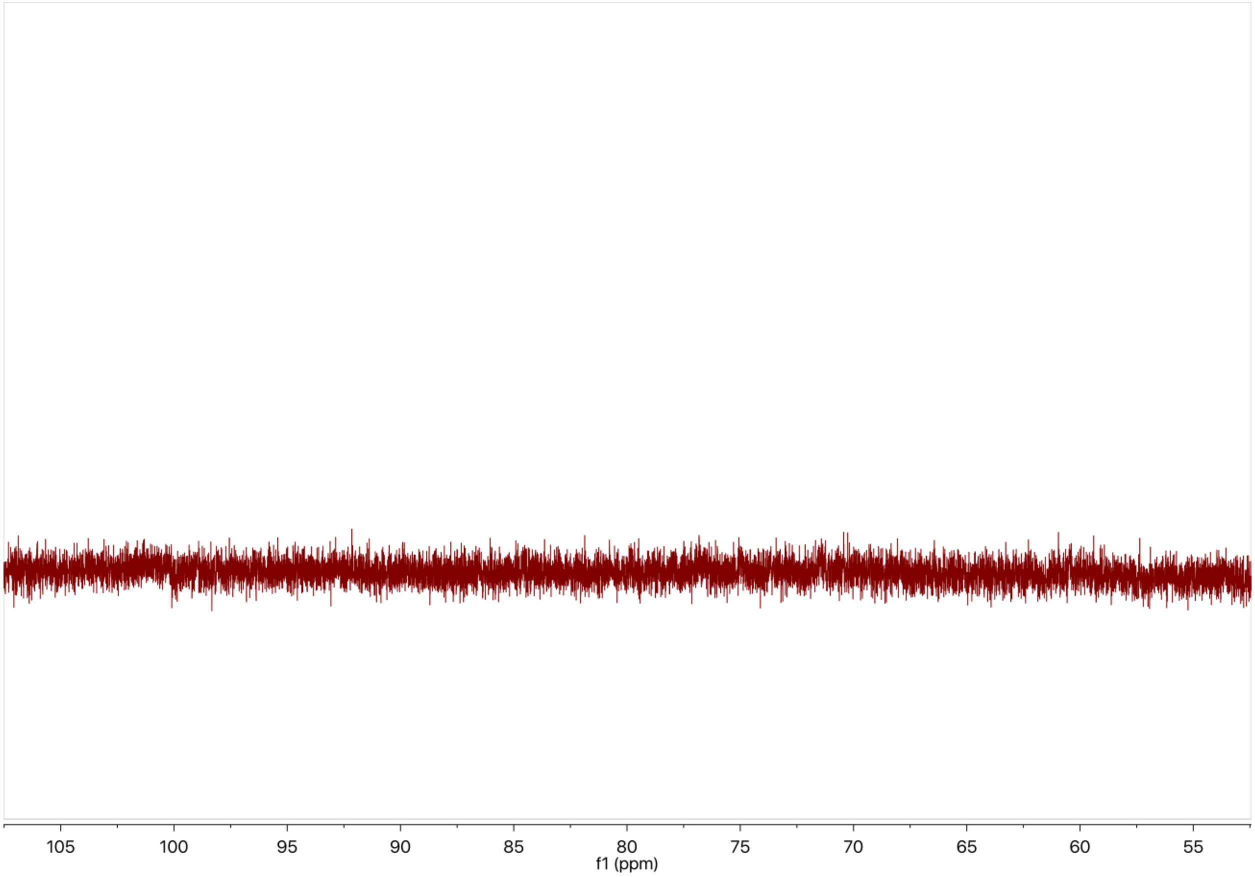

b. 5hex2 ^{13}C NMR (201 MHz, Deuterium Oxide) No signal detected.

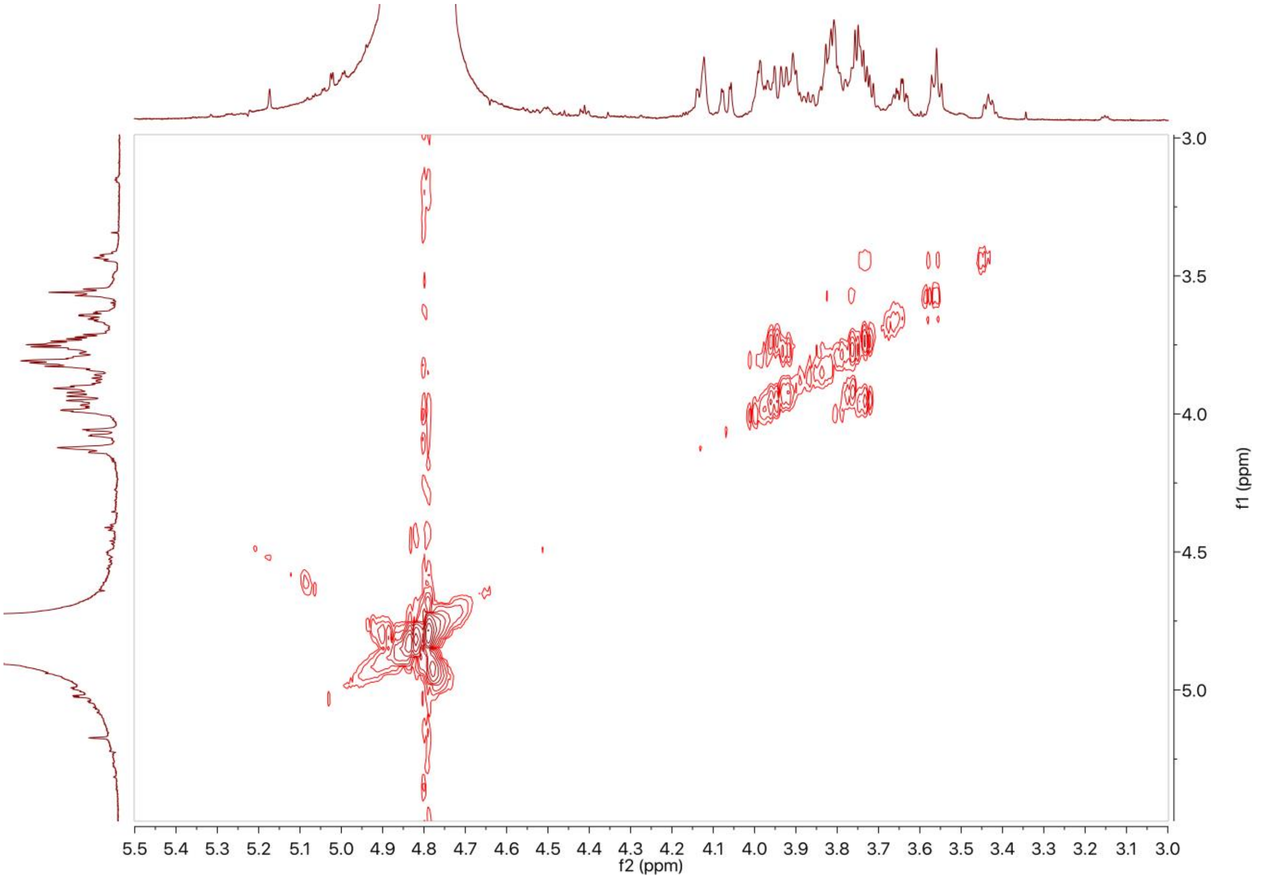

c. 5hex2 COSY

^{1}H NMR (800 MHz, Deuterium Oxide) δ 4.80, 4.80, 4.92, 4.01, 4.00, 3.74, 3.96, 3.96, 3.73, 3.93, 3.92, 3.77, 3.86, 3.85, 3.79, 3.98, 3.93, 3.76, 3.92, 3.77, 3.73, 3.95, 3.73, 3.95, 3.68, 3.66, 3.58, 3.57, 3.58, 3.45

^{1}H NMR (800 MHz, Deuterium Oxide) δ 4.90, 4.89, 4.78, 4.01, 4.00, 3.96, 3.96, 3.95, 3.95, 3.93, 3.92, 3.92, 3.87, 3.84, 3.79, 3.79, 3.77, 3.76, 3.76, 3.75, 3.73, 3.73, 3.72, 3.72, 3.68, 3.66, 3.59, 3.56, 3.56, 3.45.

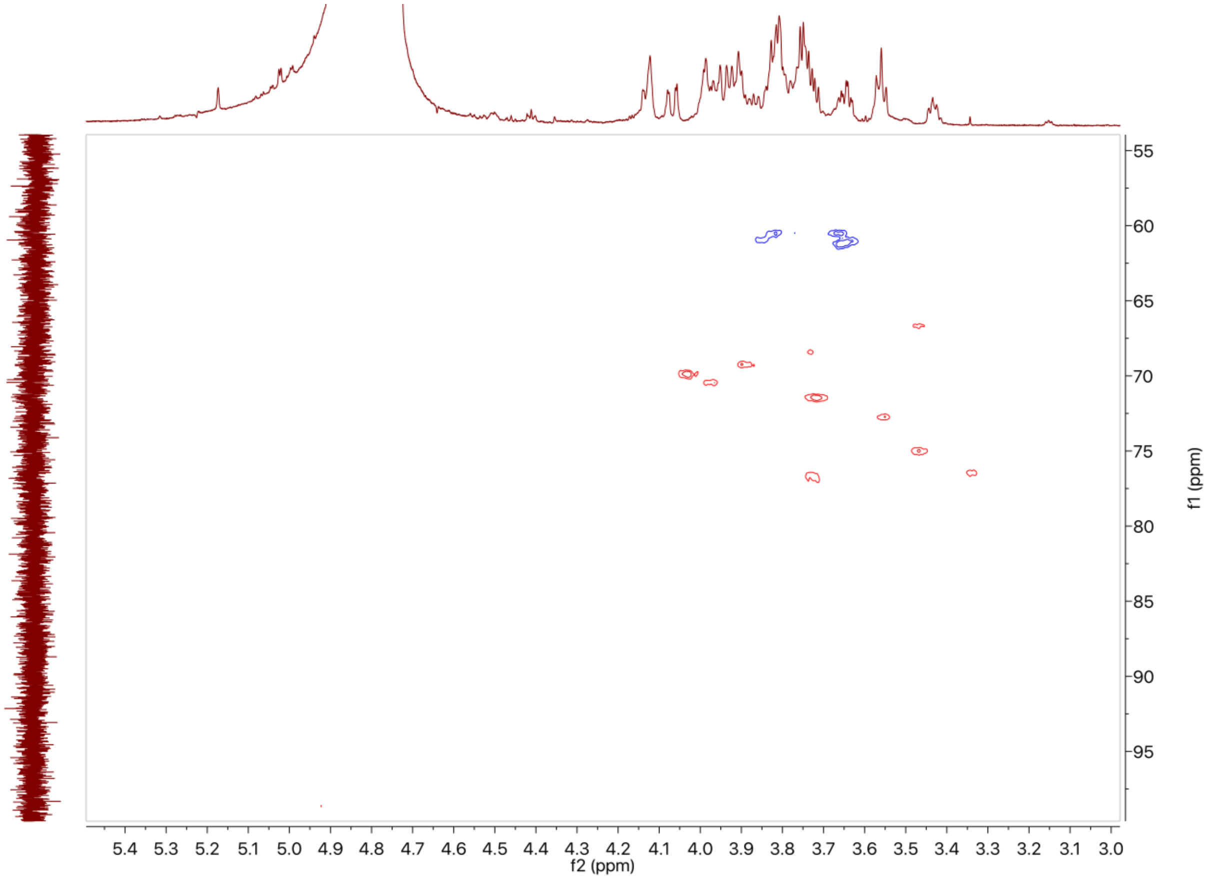

d. 5hex2 HSQC

^{13}C NMR (201 MHz, Deuterium Oxide) δ 69.89, 70.46, 69.24, 60.53, 71.46, 61.21, 61.11, 72.73, 74.99, 76.47

^{1}H NMR (800 MHz, Deuterium Oxide) δ 4.03, 3.97, 3.90, 3.82, 3.72, 3.66, 3.64, 3.55, 3.47, 3.34.

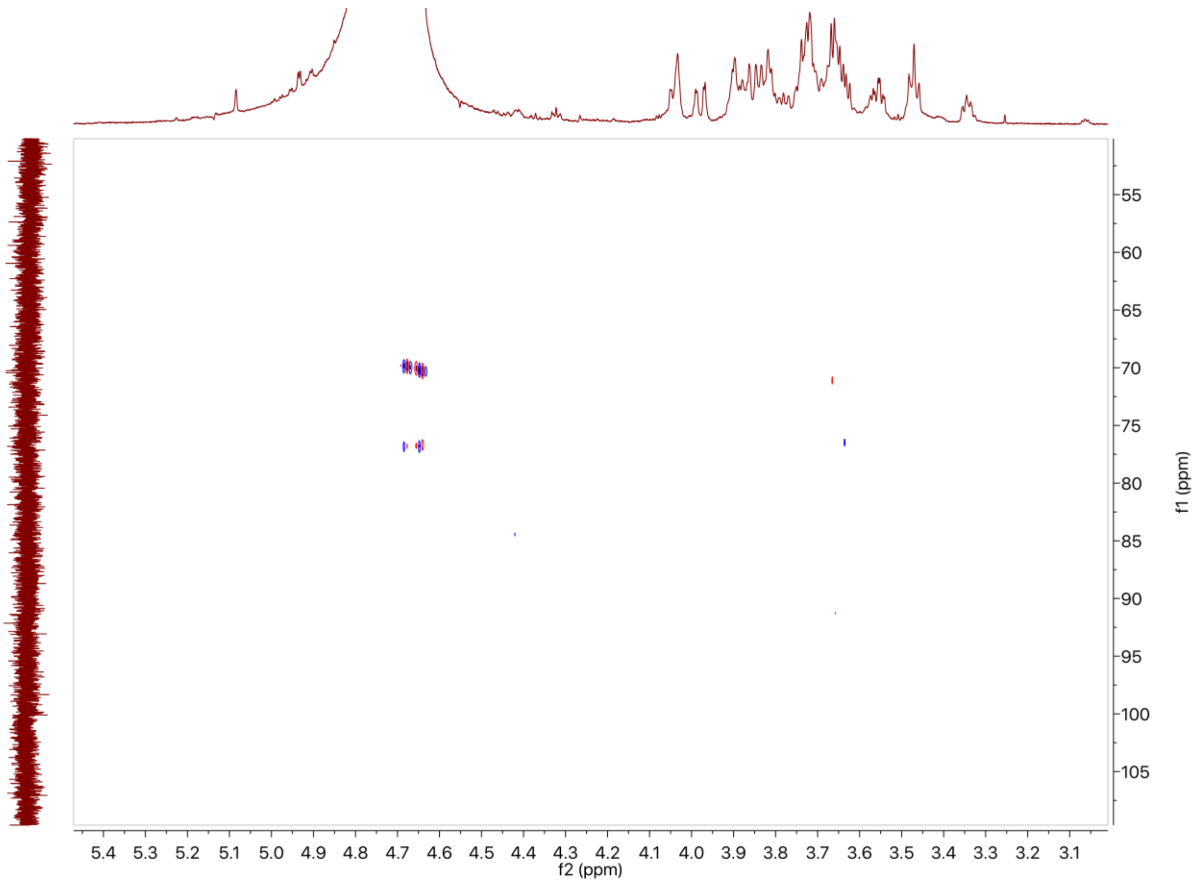

e. 5hex2 HMBC

^{13}C NMR (201 MHz, Deuterium Oxide) δ 69.85, 69.86, 70.06, 70.22, 70.33, 70.36

^{1}H NMR (800 MHz, Deuterium Oxide) δ 4.69, 4.68, 4.66, 4.65, 4.64, 4.63.

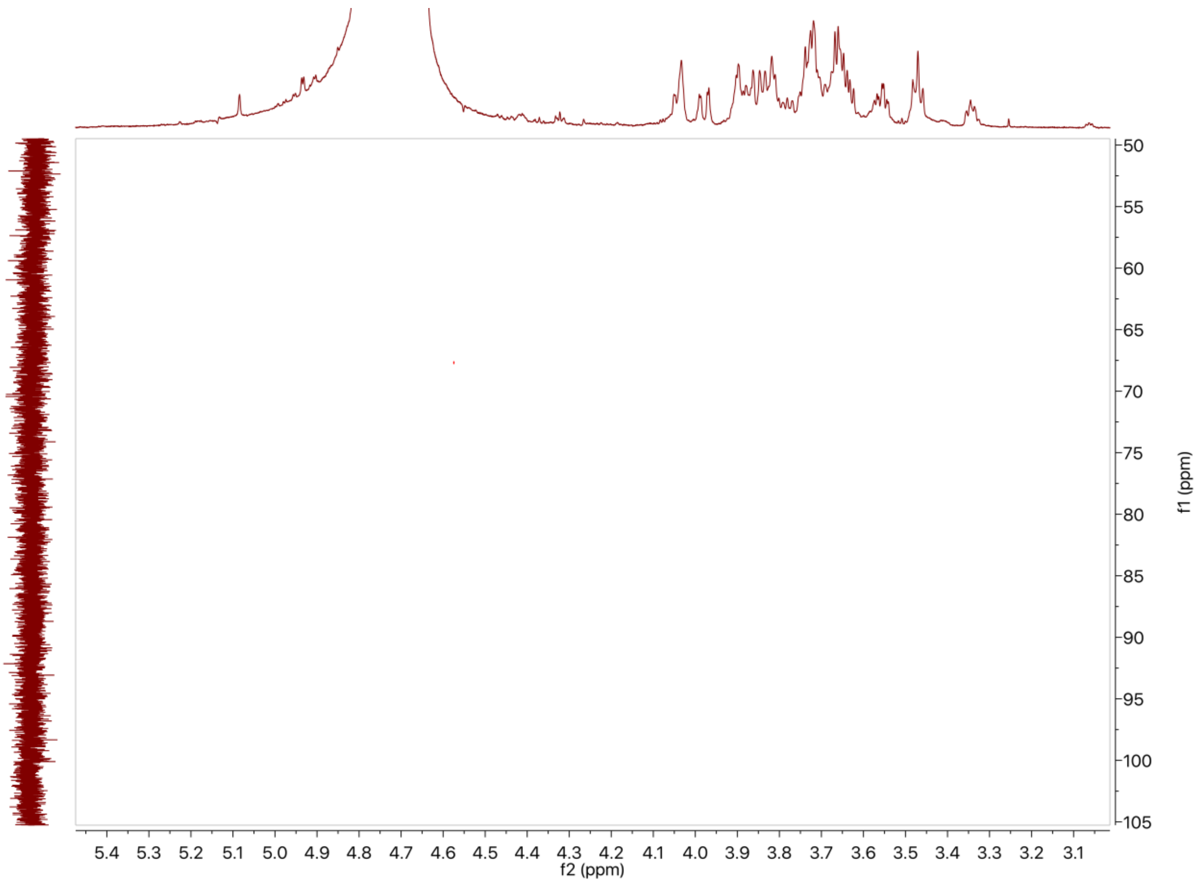

f. 5hex2 H2BC

No signal detected.

g. 5hex2 MS/MS

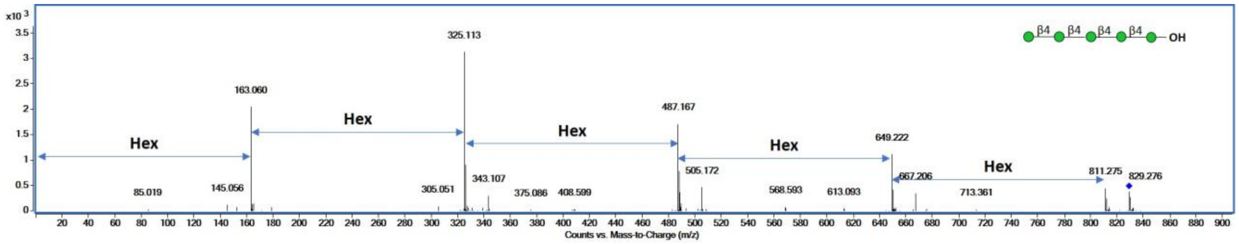
x10 3
325.113
163.060
487.167
649.222
505.172
343.107
667.206
811.275
829.276
85.019
145.056
305.051
375.086
408.599
568.593
613.093
713.361
Hex
Hex
Hex
Hex
Hex
β4
β4
β4
β4
OH
Counts vs. Mass-to-Charge (m/z)

F. 4hex2

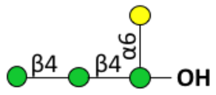

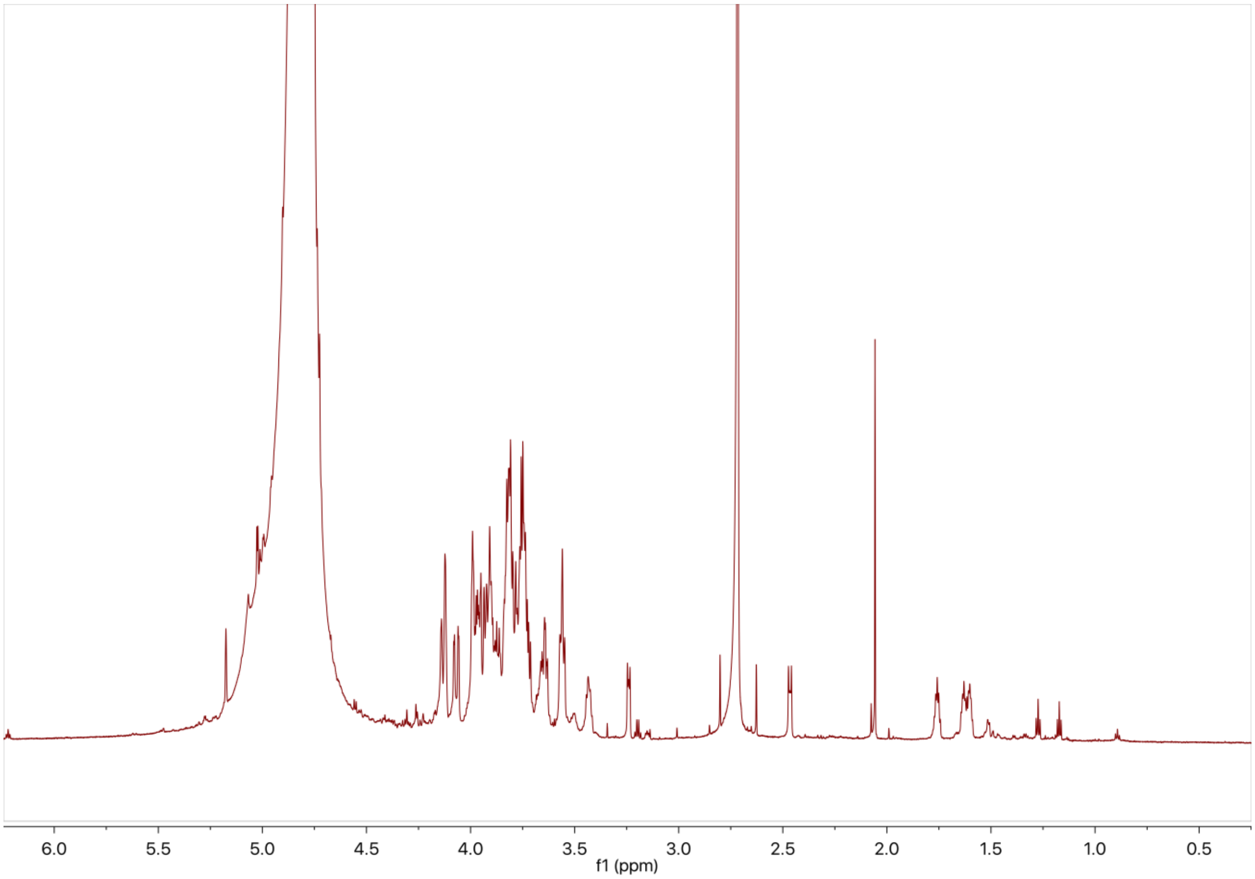

a. 4hex2 ^{1}H NMR (800 MHz, Deuterium Oxide) δ 5.18, 5.17, 5.07, 5.03, 5.02, 4.73, 4.14, 4.12, 4.08, 4.06, 3.99, 3.97, 3.97, 3.95, 3.93, 3.92, 3.91, 3.89, 3.87, 3.86, 3.83, 3.82, 3.81, 3.80, 3.78, 3.76, 3.76, 3.75, 3.74, 3.73, 3.72, 3.71, 3.64, 3.56, 3.43.

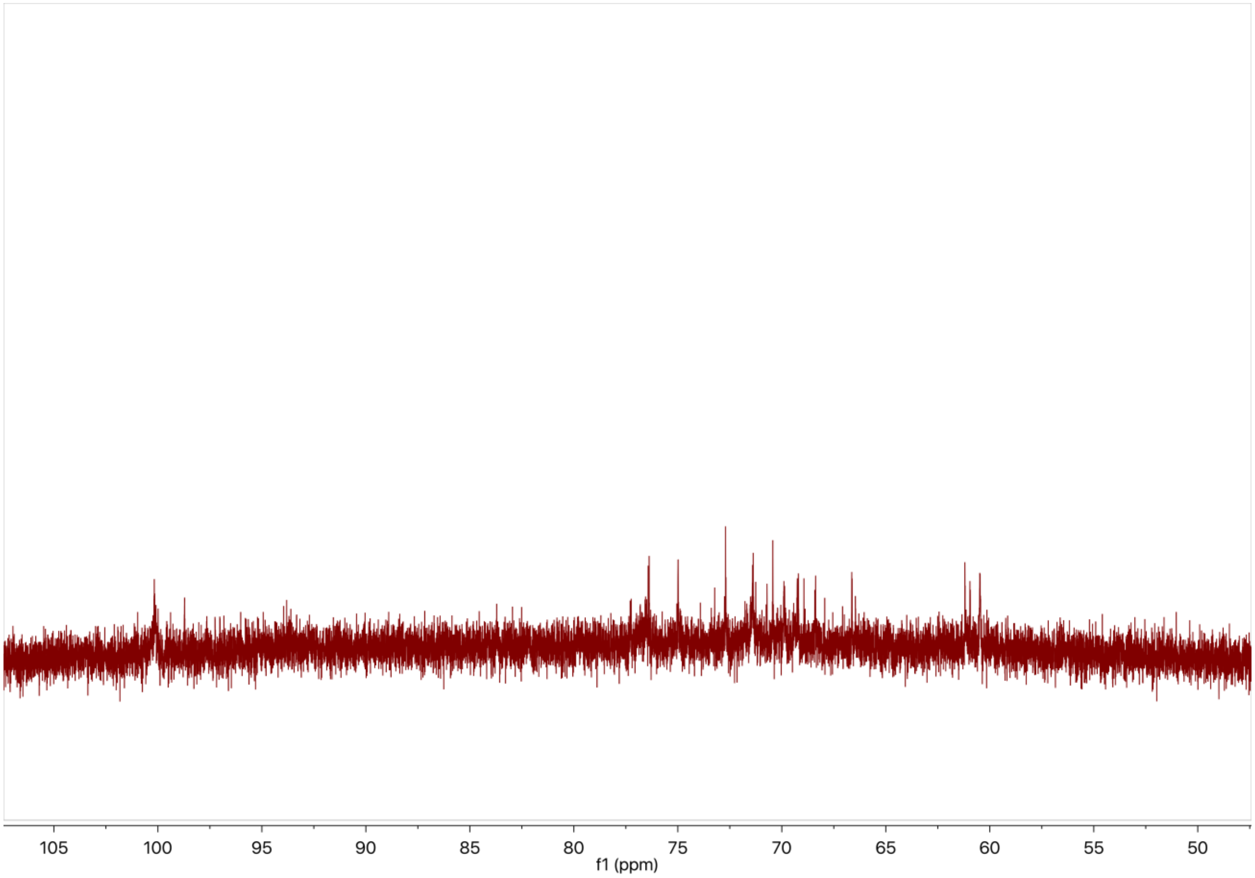

b. 4hex2 ^{13}C NMR (201 MHz, Deuterium Oxide) δ 100.18, 98.72, 76.43, 74.98, 72.71, 71.37, 70.71, 70.43, 69.20, 68.94, 68.38, 66.62, 61.20, 60.48.

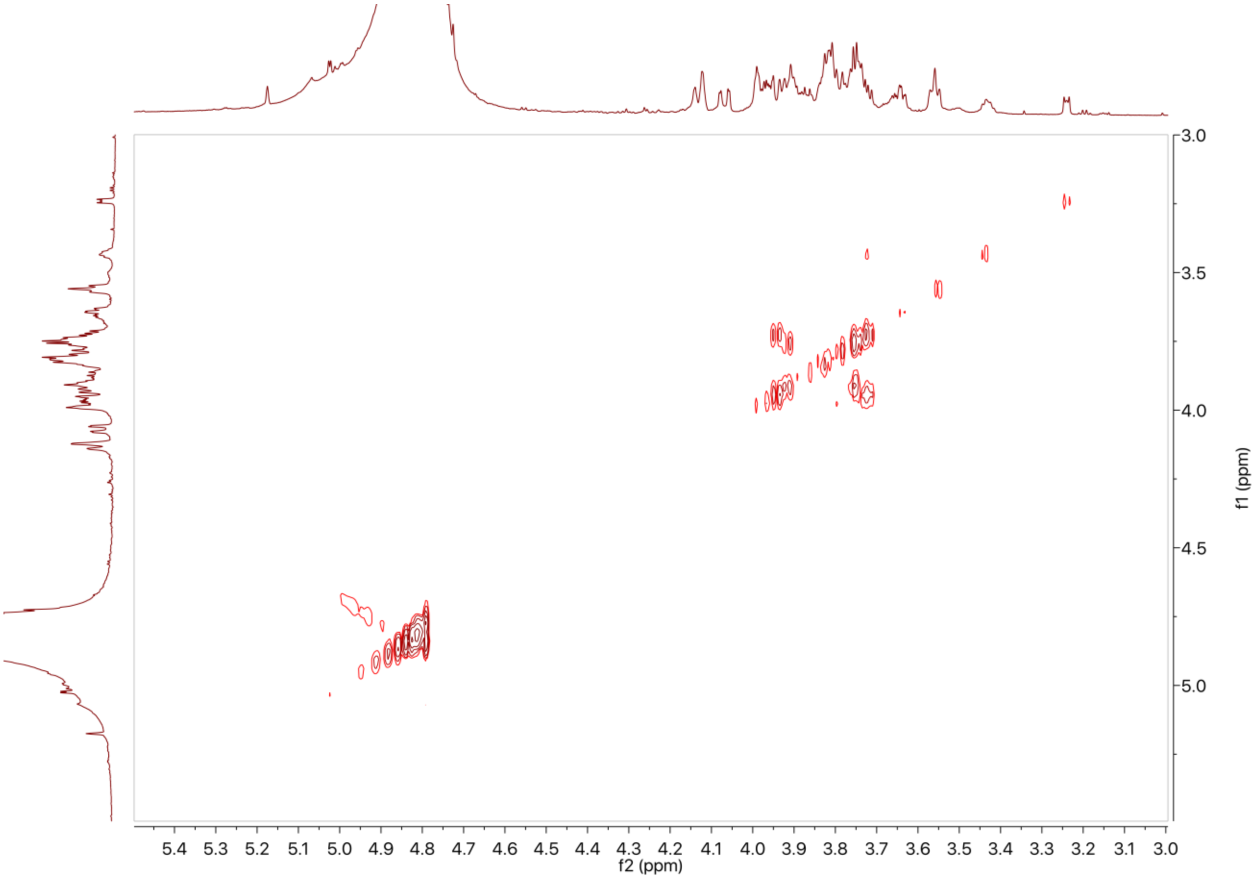

c. 4hex2 COSY

^{1}H NMR (800 MHz, Deuterium Oxide) δ 5.03, 4.68, 4.71, 4.95, 4.75, 4.91, 4.78, 4.88, 4.86, 4.84, 4.83, 4.81, 4.78, 3.98, 3.97, 3.73, 3.94, 3.94, 3.72, 3.75, 3.91, 3.92, 3.76, 3.88, 3.87, 3.82, 3.83, 3.82, 3.78, 3.98, 3.78, 3.75, 3.91, 3.75, 3.72, 3.94, 3.43, 3.94, 3.73, 3.65, 3.64, 3.56, 3.56, 3.44, 3.43, 3.24, 3.24

^{1}H NMR (800 MHz, Deuterium Oxide) δ 5.02, 4.99, 4.97, 4.95, 4.93, 4.91, 4.90, 4.88, 4.86, 4.84, 4.82, 4.81, 4.79, 3.99, 3.97, 3.95, 3.95, 3.93, 3.93, 3.92, 3.92, 3.91, 3.91, 3.89, 3.86, 3.84, 3.83, 3.81, 3.80, 3.80, 3.78, 3.76, 3.75, 3.74, 3.73, 3.72, 3.72, 3.71, 3.71, 3.64, 3.63, 3.56, 3.55, 3.44, 3.43, 3.25, 3.23.

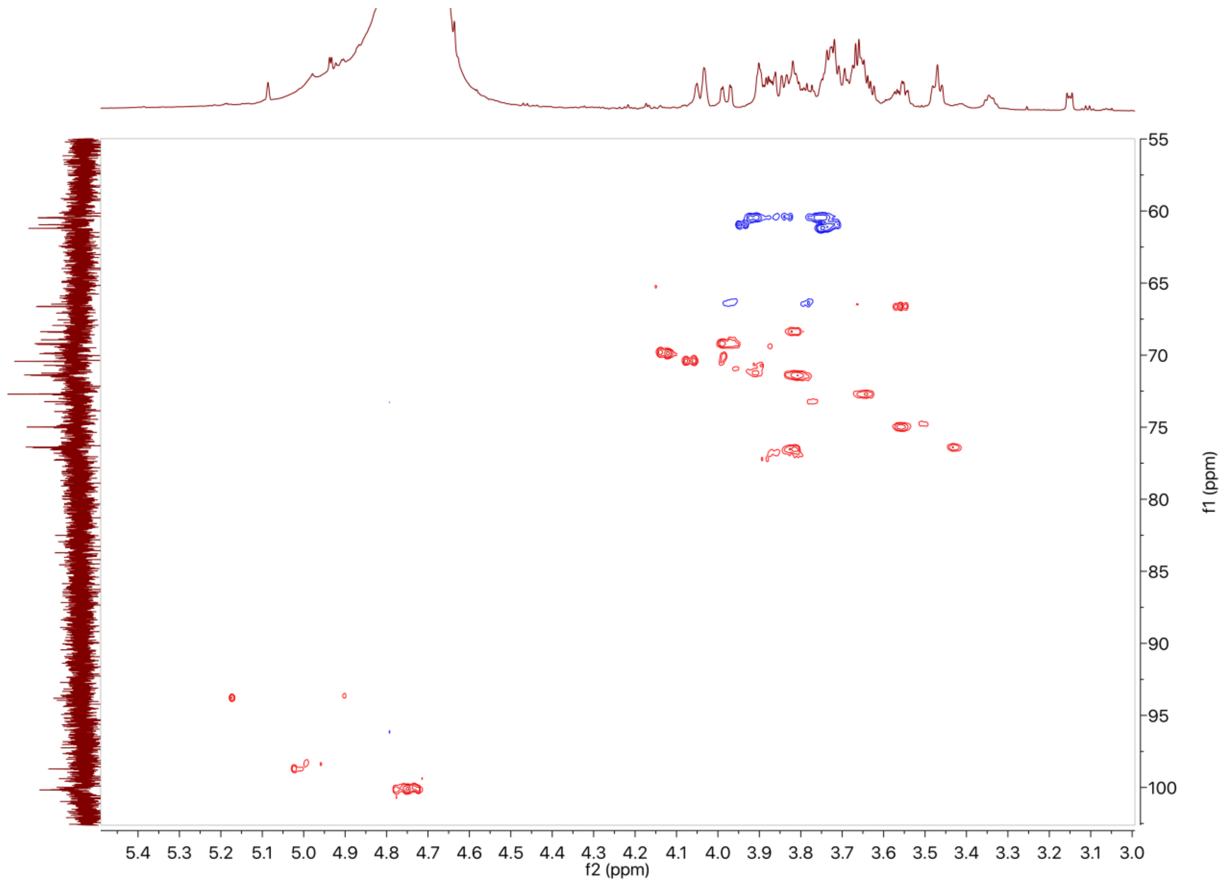

d. 4hex2 HSQC

^{13}C NMR (201 MHz, Deuterium Oxide) δ 93.78, 98.70, 98.34, 98.29, 100.14, 100.10, 100.01, 100.14, 69.79, 69.88, 70.40, 70.40, 70.47, 69.20, 70.07, 60.95, 60.91, 60.45, 71.24, 70.72, 77.23, 68.37, 76.52, 71.41, 66.39, 73.21, 60.46, 61.19, 72.70, 66.62, 74.98, 76.39

^{1}H NMR (800 MHz, Deuterium Oxide) δ 5.17, 5.02, 4.99, 4.99, 4.78, 4.75, 4.73, 4.72, 4.14, 4.12, 4.08, 4.06, 3.99, 3.99, 3.99, 3.95, 3.93, 3.91, 3.91, 3.89, 3.88, 3.82, 3.81, 3.81, 3.78, 3.77, 3.75, 3.75, 3.64, 3.56, 3.56, 3.43.

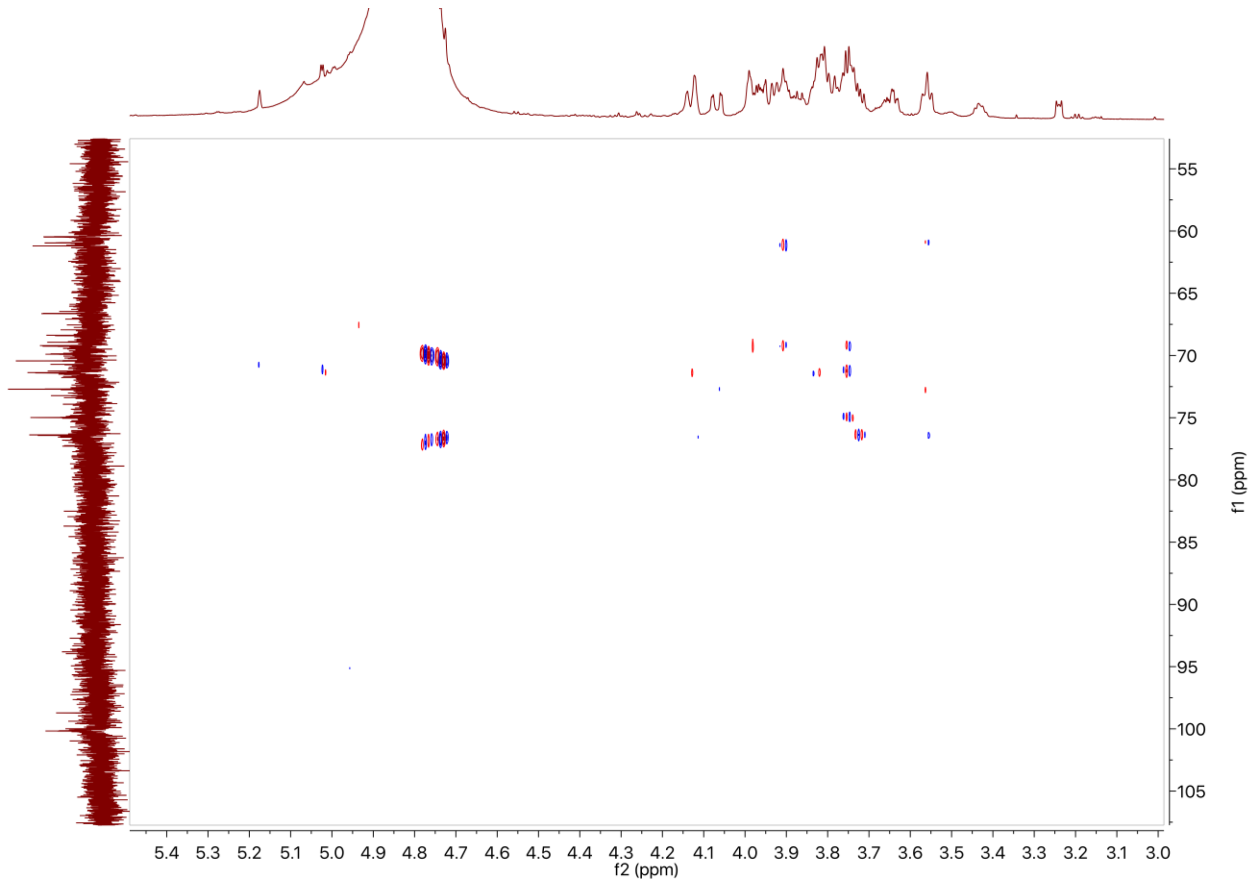

e. 4hex2 HMBC

^{13}C NMR (201 MHz, Deuterium Oxide) δ 69.82, 69.86, 77.02, 76.85, 69.96, 70.00, 76.69, 70.07, 76.74, 70.36, 76.67, 70.43, 76.60, 70.42, 61.13, 61.15, 71.36, 71.24, 71.22, 76.34, 76.38, 76.36

^{1}H NMR (800 MHz, Deuterium Oxide) δ 4.78, 4.77, 4.77, 4.77, 4.77, 4.76, 4.75, 4.75, 4.74, 4.74, 4.73, 4.73, 4.72, 4.72, 3.91, 3.90, 3.82, 3.75, 3.75, 3.73, 3.72, 3.72.

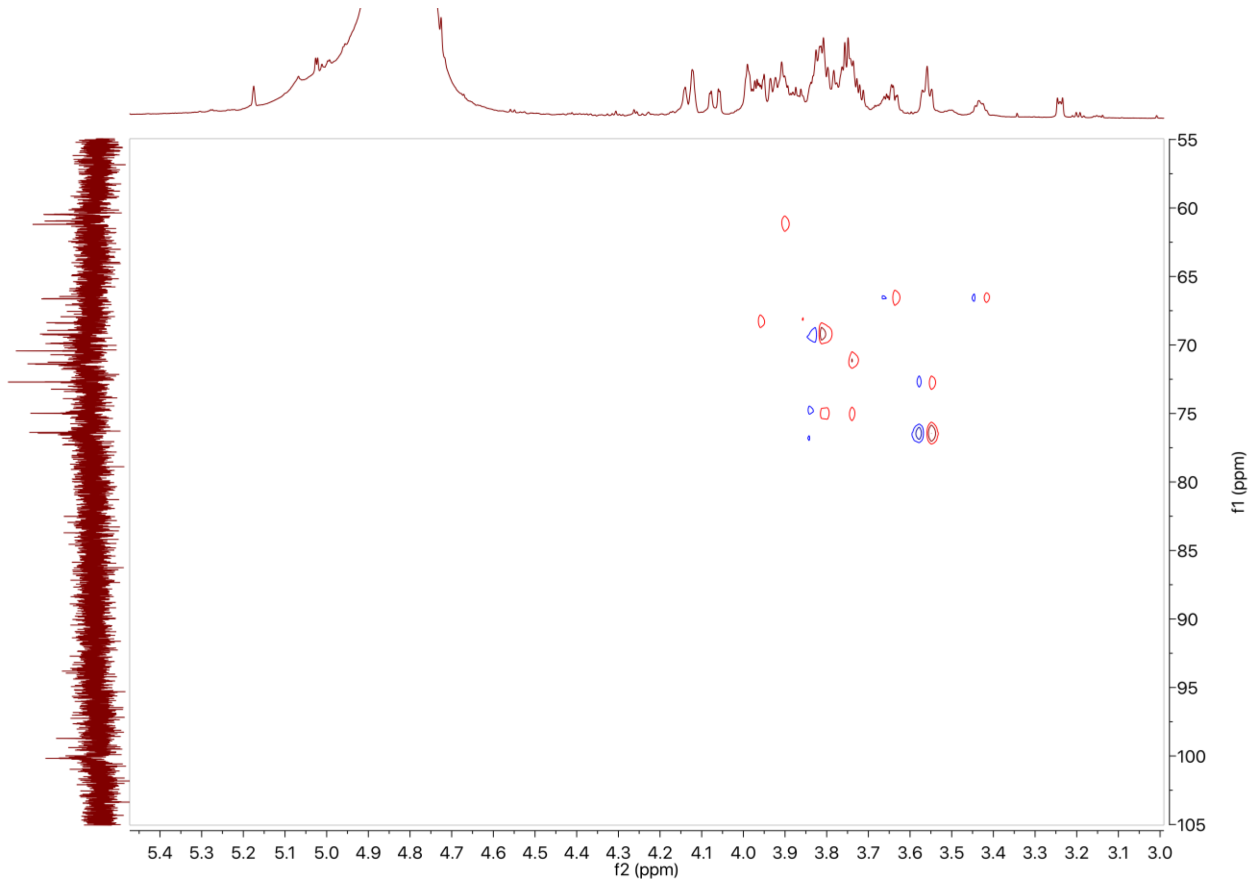

f. 4hex2 H2BC

^{13}C NMR (201 MHz, Deuterium Oxide) δ 69.23, 69.19, 71.14, 66.56, 76.45, 76.44, 66.55

^{1}H NMR (800 MHz, Deuterium Oxide) δ 3.83, 3.81, 3.74, 3.63, 3.58, 3.55, 3.42.

g. 4hex2 MS/MS

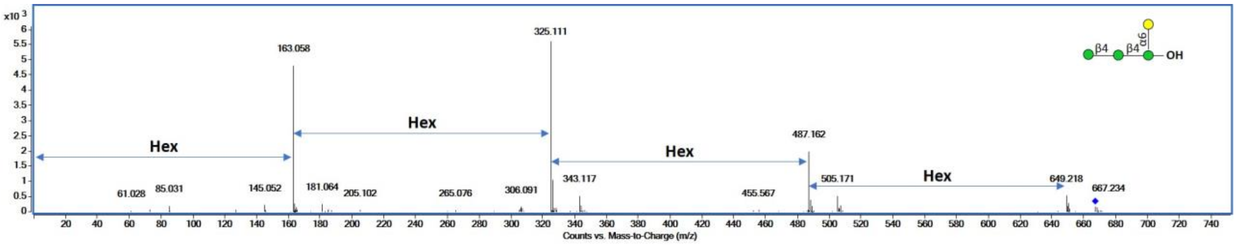

x10 3
163.058
325.111
487.162
649.218
667.234
61.028
85.031
145.052
181.064
205.102
265.076
306.091
343.117
455.567
505.171
Hex
Hex
Hex
Hex
β4
β4
α6
OH
Counts vs. Mass-to-Charge (m/z)

G. 5hex3

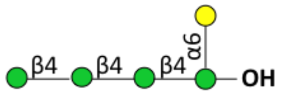

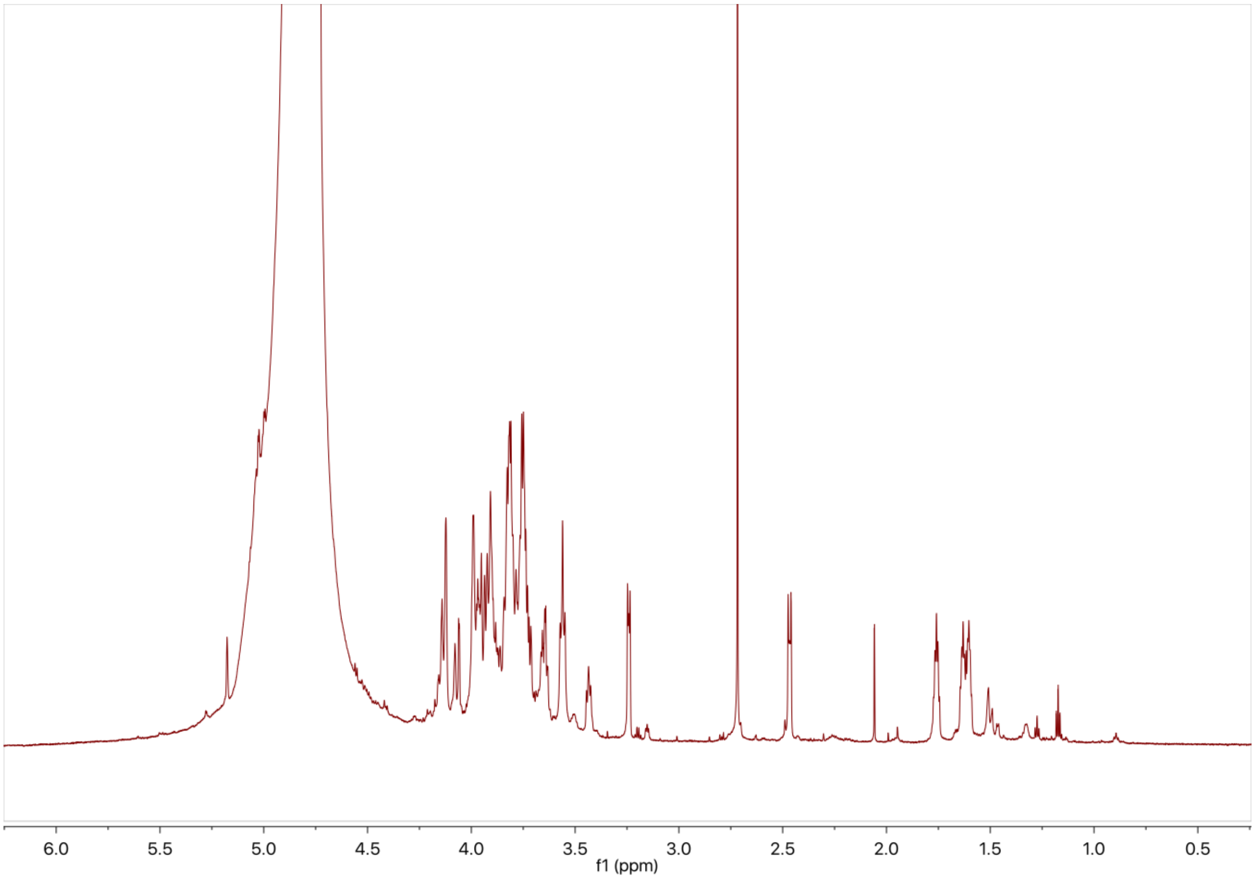

a. 5hex3 ^{1}H NMR (800 MHz, Deuterium Oxide) δ 5.18, 4.73, 4.69, 4.66, 4.14, 4.12, 4.08, 4.06, 4.00, 3.99, 3.97, 3.97, 3.96, 3.95, 3.94, 3.92, 3.91, 3.90, 3.89, 3.88, 3.87, 3.86, 3.84, 3.83, 3.81, 3.81, 3.80, 3.78, 3.77, 3.76, 3.75, 3.74, 3.73, 3.72, 3.71, 3.66, 3.64, 3.63, 3.57, 3.56, 3.55, 3.43.

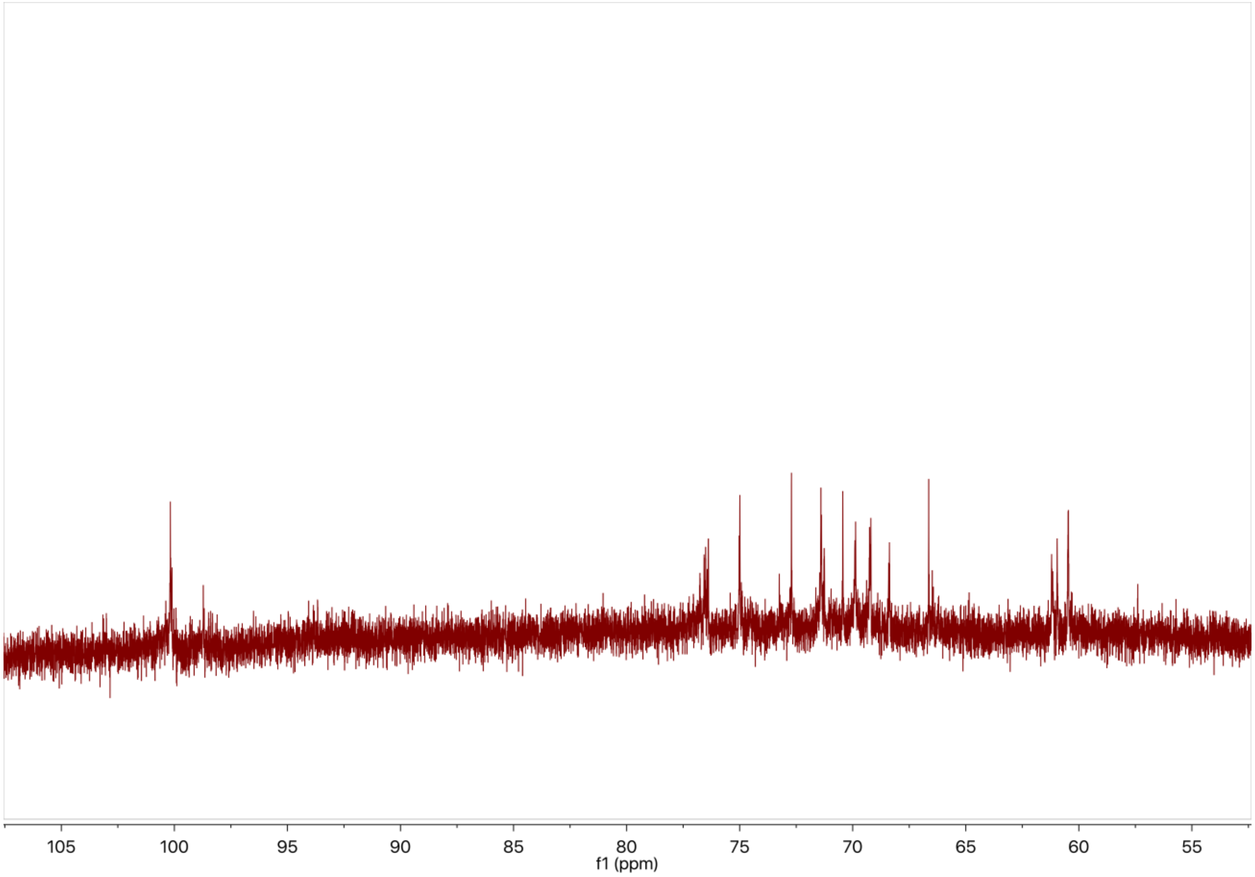

b. 5hex3 ^{13}C NMR (201 MHz, Deuterium Oxide) δ 163.26, 163.08, 162.90, 100.18, 100.11, 76.57, 76.50, 76.38, 74.99, 72.71, 71.41, 70.44, 69.87, 69.20, 68.38, 66.64, 61.20, 60.96, 60.47.

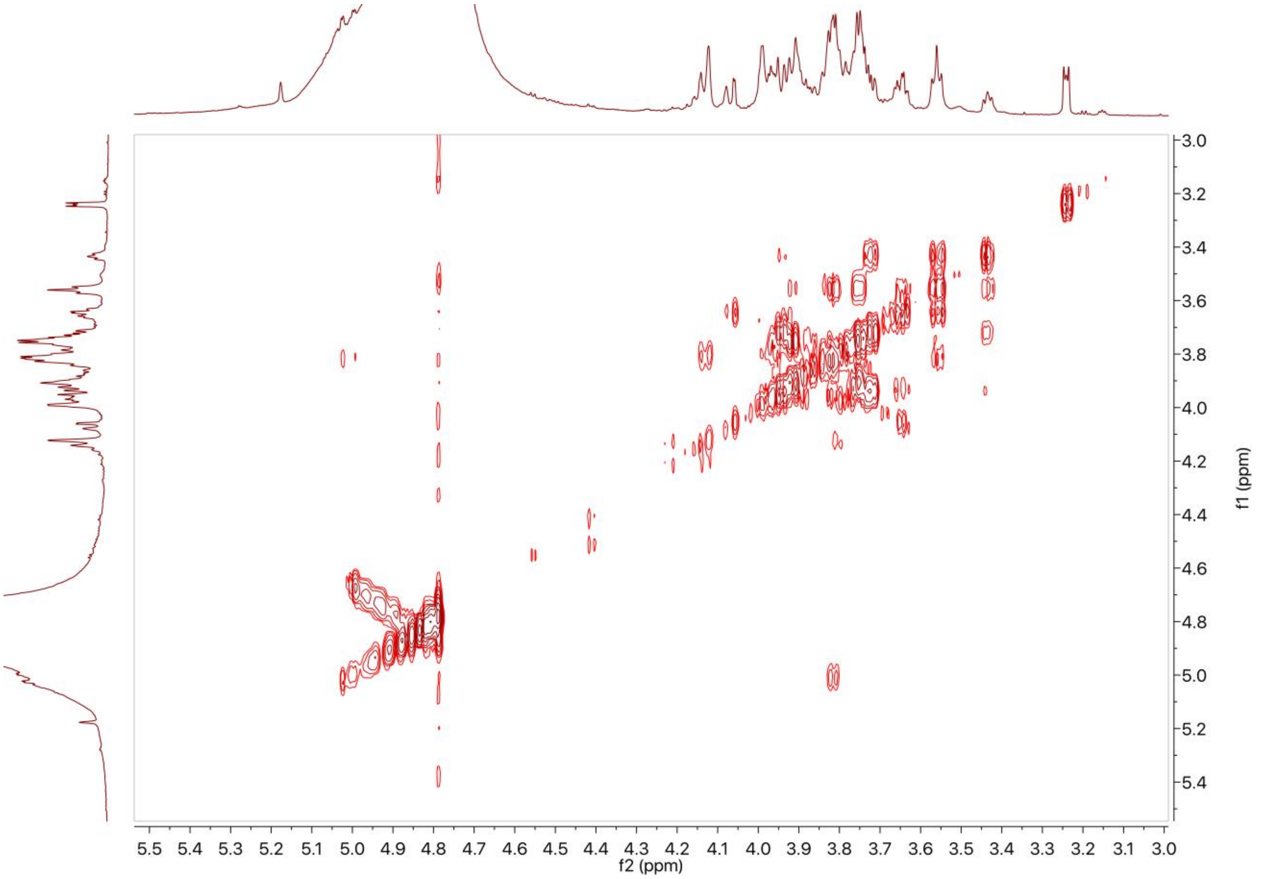

c. 5hex3 COSY

^{1}H NMR (800 MHz, Deuterium Oxide) δ 3.81, 4.66, 4.67, 4.70, 4.74, 4.77, 4.55, 4.55, 4.41, 4.51, 4.51, 4.21, 4.13, 4.21, 4.13, 4.16, 4.15, 4.14, 3.81, 4.11, 3.80, 4.08, 3.64, 4.06, 3.64, 4.02, 4.00, 3.68, 3.98, 3.97, 3.97, 3.77, 3.72, 3.94, 3.43, 3.94, 3.72, 3.75, 3.55, 3.91, 3.75, 3.91, 3.88, 3.88, 3.98, 3.74, 3.86, 3.75, 3.86, 3.83, 3.54, 3.96, 3.83, 3.56, 3.74, 5.01, 3.96, 3.56, 3.82, 4.12, 5.01, 3.55, 3.97, 3.79, 3.98, 3.78, 3.95, 3.77, 3.92, 3.55, 3.75, 3.91, 3.75, 3.94, 3.72, 3.43, 3.94, 3.43, 3.72, 4.02, 3.68, 3.69, 3.66, 3.93, 3.65, 4.05, 3.65, 3.93, 4.06, 3.64, 3.43, 3.65, 3.55, 3.81, 3.74, 3.82, 3.56, 3.64, 3.56, 3.43, 3.64, 3.50, 3.43, 3.94, 3.72, 3.55, 3.43, 3.24, 3.24, 3.19, 3.15

^{1}H NMR (800 MHz, Deuterium Oxide) δ 5.02, 5.00, 4.99, 4.96, 4.93, 4.89, 4.56, 4.55, 4.42, 4.42, 4.40, 4.23, 4.23, 4.21, 4.21, 4.18, 4.16, 4.14, 4.14, 4.12, 4.12, 4.08, 4.08, 4.06, 4.06, 4.02, 4.00, 4.00, 3.99, 3.98, 3.96, 3.96, 3.95, 3.95, 3.95, 3.93, 3.93, 3.92, 3.92, 3.92, 3.91, 3.91, 3.89, 3.88, 3.88, 3.88, 3.87, 3.86, 3.86, 3.84, 3.84, 3.83, 3.82, 3.82, 3.82, 3.82, 3.82, 3.82, 3.82, 3.81, 3.81, 3.81, 3.80, 3.80, 3.79, 3.78, 3.78, 3.77, 3.77, 3.76, 3.75, 3.75, 3.74, 3.73, 3.72, 3.72, 3.71, 3.71, 3.71, 3.69, 3.69, 3.68, 3.67, 3.66, 3.66, 3.65, 3.64, 3.64, 3.64, 3.63, 3.57, 3.57, 3.57, 3.57, 3.56, 3.56, 3.56, 3.56, 3.55, 3.55, 3.55, 3.52, 3.44, 3.44, 3.43, 3.43, 3.43, 3.24, 3.23, 3.19, 3.14.

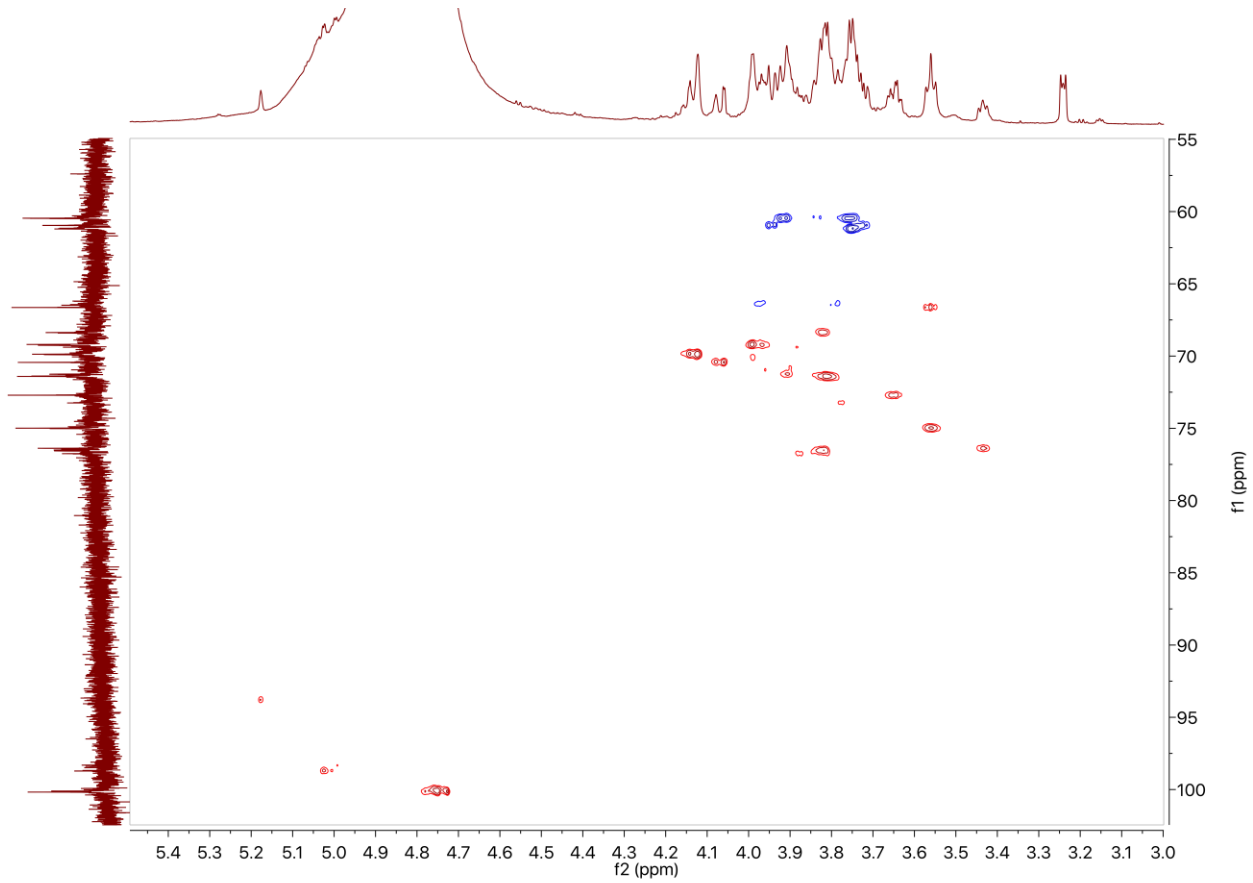

d. 5hex3 HSQC

^{13}C NMR (201 MHz, Deuterium Oxide) δ 93.79, 98.68, 100.09, 100.13, 69.83, 69.87, 70.40, 70.43, 69.19, 70.09, 66.37, 60.95, 60.91, 60.47, 60.45, 71.25, 68.37, 76.52, 71.40, 66.35, 73.23, 60.47, 61.17, 72.69, 66.62, 74.98, 76.38

^{1}H NMR (800 MHz, Deuterium Oxide) δ 5.18, 5.02, 4.75, 4.73, 4.14, 4.12, 4.08, 4.06, 3.99, 3.99, 3.98, 3.95, 3.94, 3.92, 3.91, 3.91, 3.82, 3.82, 3.81, 3.79, 3.77, 3.76, 3.75, 3.64, 3.56, 3.56, 3.43.

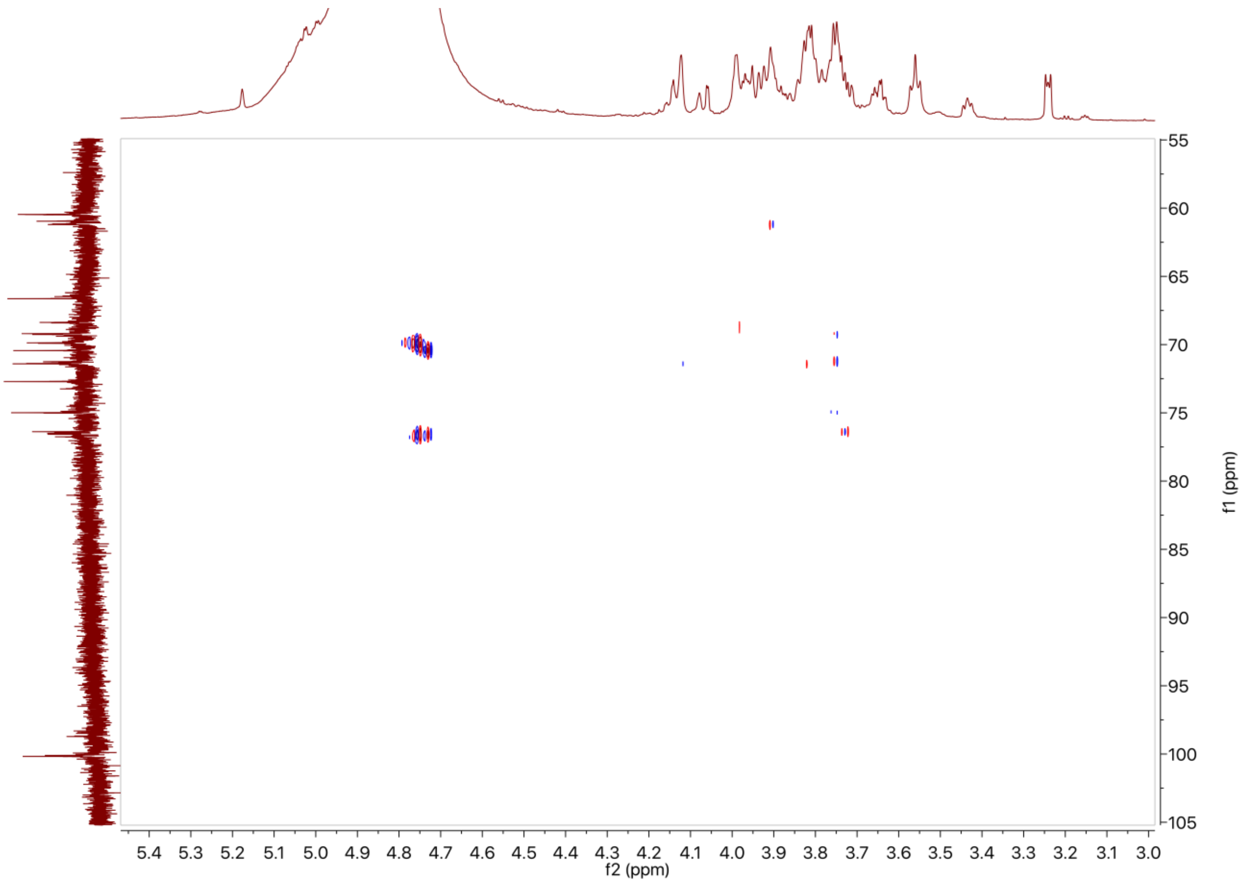

e. 5hex3 HMBC

^{13}C NMR (201 MHz, Deuterium Oxide) δ 69.86, 69.88, 76.79, 69.92, 76.68, 69.91, 76.61, 69.99, 76.58, 70.39, 76.69, 70.43, 76.61, 76.57, 70.41, 71.40, 68.62, 61.23, 61.18, 71.43, 74.93, 71.22, 69.18, 74.98, 71.24, 69.27, 76.40, 76.39, 76.38

^{1}H NMR (800 MHz, Deuterium Oxide) δ 4.78, 4.78, 4.77, 4.77, 4.76, 4.76, 4.76, 4.75, 4.75, 4.74, 4.74, 4.73, 4.73, 4.72, 4.72, 4.12, 3.98, 3.91, 3.90, 3.82, 3.76, 3.75, 3.75, 3.75, 3.75, 3.75, 3.74, 3.73, 3.72.

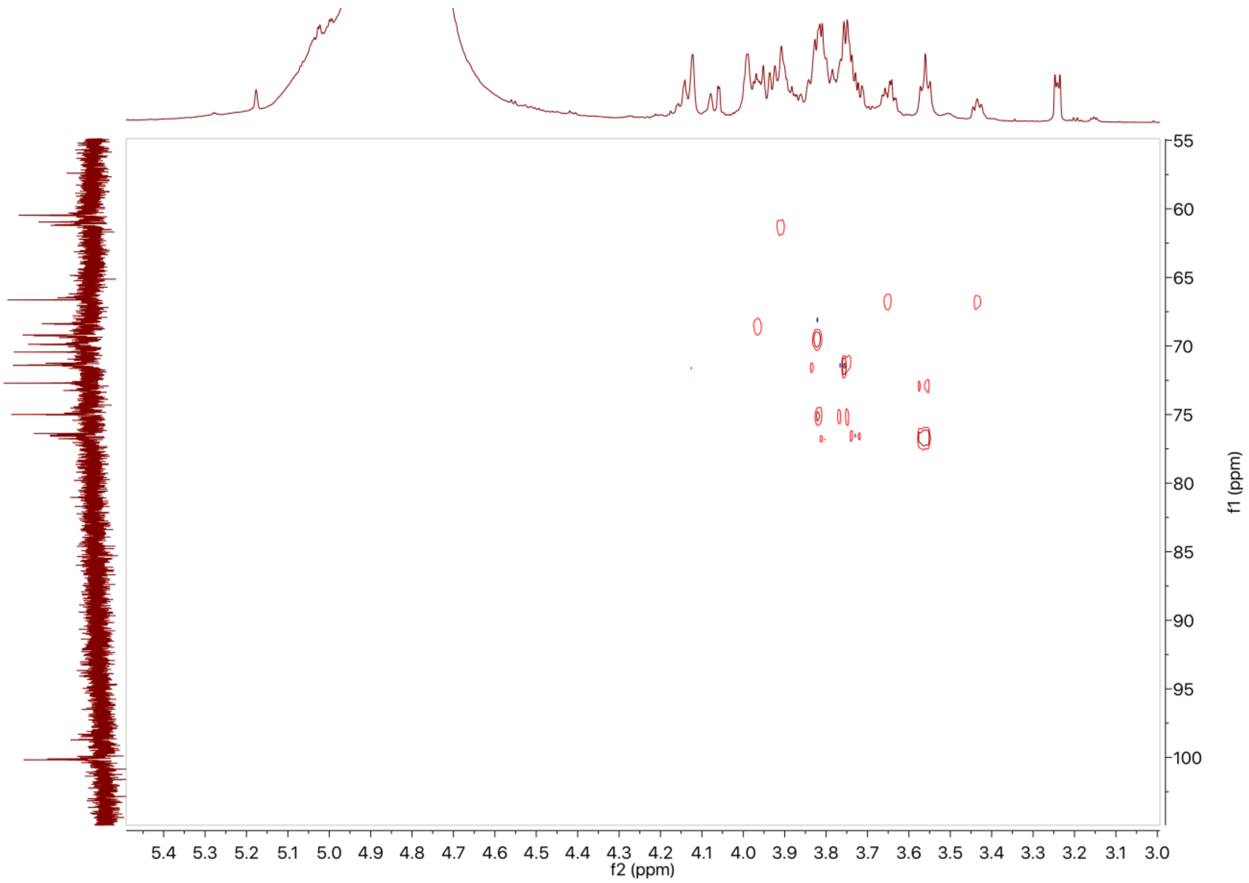

f. 5hex3 H2BC

^{13}C NMR (201 MHz, Deuterium Oxide) δ 71.62, 68.53, 61.31, 71.58, 69.51, 68.11, 75.12, 75.13, 71.40, 71.43, 75.21, 76.57, 76.53, 76.58, 66.77, 76.68, 72.91, 66.81

^{1}H NMR (800 MHz, Deuterium Oxide) δ 4.13, 3.97, 3.91, 3.84, 3.82, 3.82, 3.82, 3.77, 3.77, 3.76, 3.75, 3.74, 3.73, 3.72, 3.65, 3.56, 3.55, 3.44.

g. 5hex3 MS/MS

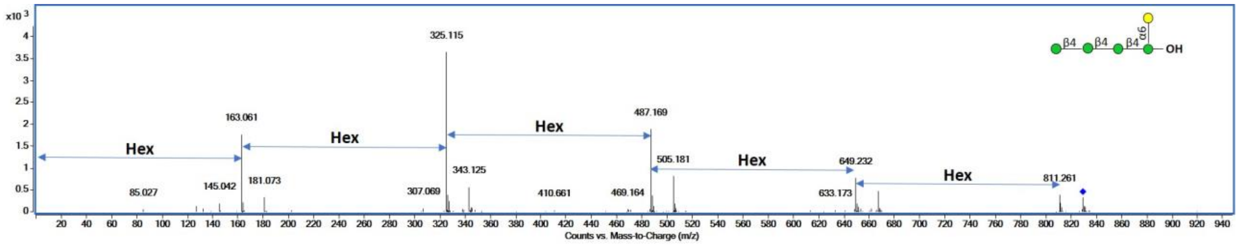

x10 3
4
3.5
3
2.5
2
1.5
1
0.5
0
325.115
163.061
487.169
505.181
649.232
811.261
85.027
145.042
181.073
307.069
343.125
410.661
469.164
633.173
Hex
Hex
Hex
Hex
Hex
β4
β4
β4
α6
OH
20 40 60 80 100 120 140 160 180 200 220 240 260 280 300 320 340 360 380 400 420 440 460 480 500 520 540 560 580 600 620 640 660 680 700 720 740 760 780 800 820 840 860 880 900 920 940
Counts vs. Mass-to-Charge (m/z)

H. 6hex2

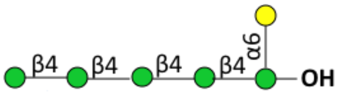

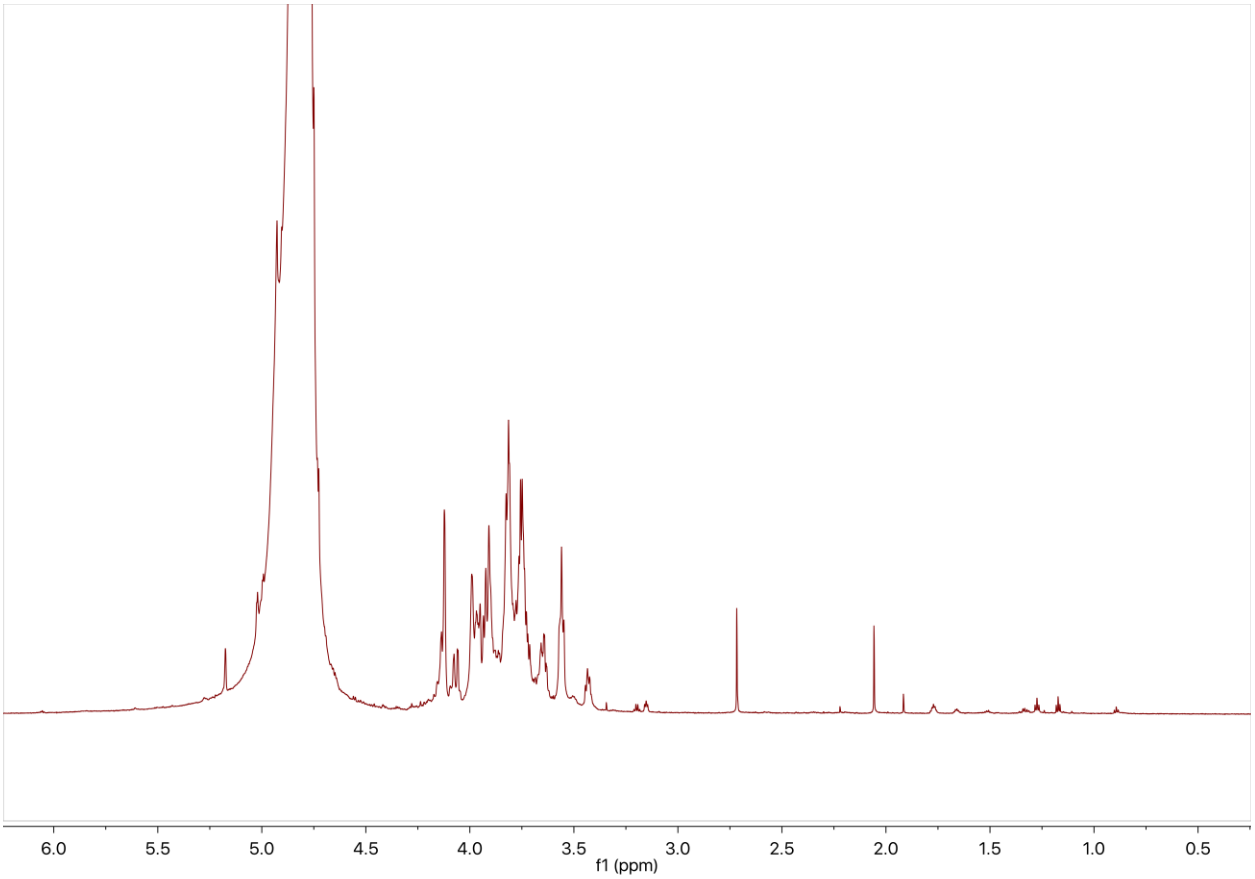

a. 6hex2 ^{1}H NMR (800 MHz, Deuterium Oxide) δ 5.18, 4.93, 4.73, 4.14, 4.14, 4.12, 4.08, 4.08, 4.06, 4.06, 3.99, 3.99, 3.97, 3.96, 3.95, 3.93, 3.92, 3.91, 3.90, 3.89, 3.88, 3.88, 3.86, 3.86, 3.83, 3.81, 3.81, 3.81, 3.79, 3.79, 3.78, 3.76, 3.76, 3.75, 3.74, 3.74, 3.73, 3.72, 3.71, 3.66, 3.66, 3.65, 3.64, 3.64, 3.57, 3.57, 3.56, 3.55, 3.43.

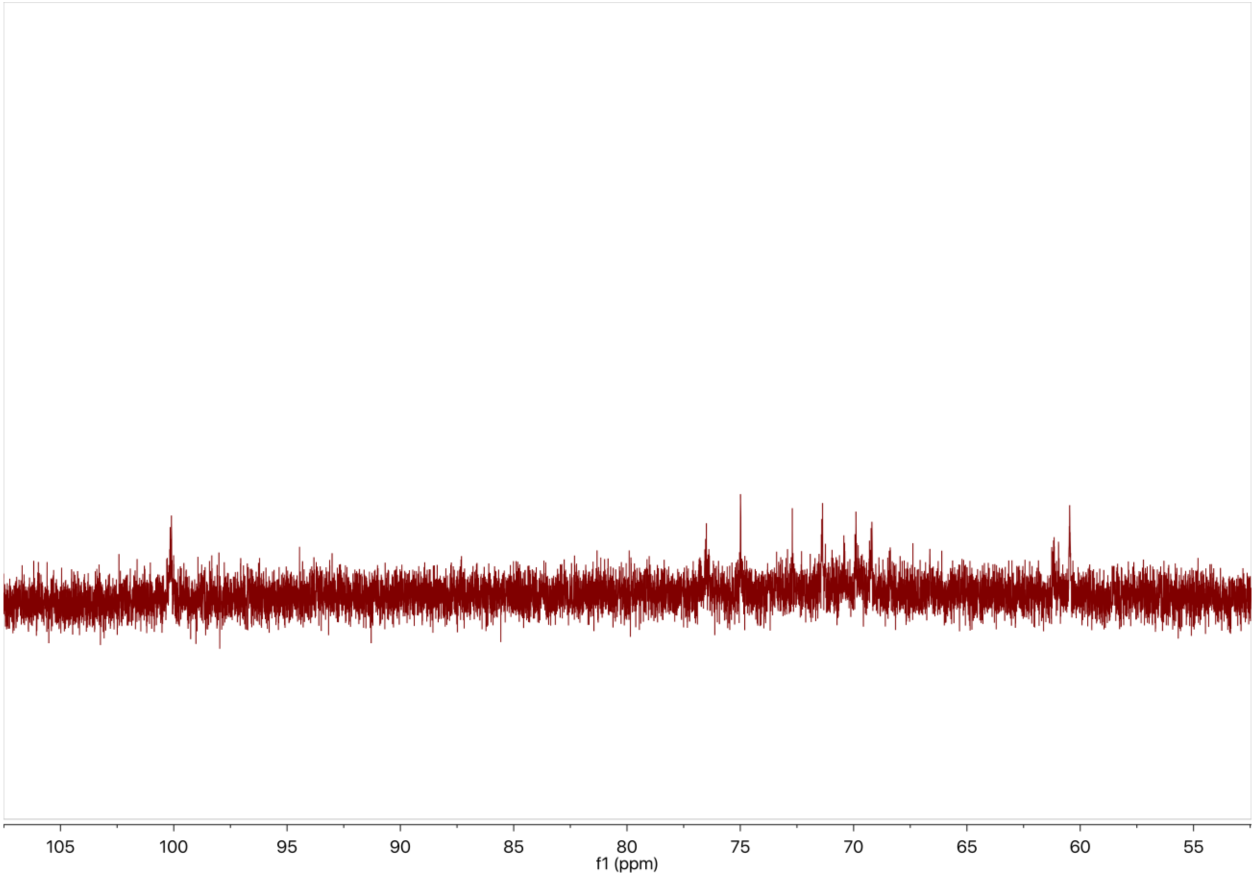

b. 6hex2 ^{13}C NMR (201 MHz, Deuterium Oxide) δ 100.10, 76.50, 74.99, 72.71, 71.37, 69.90, 60.47.

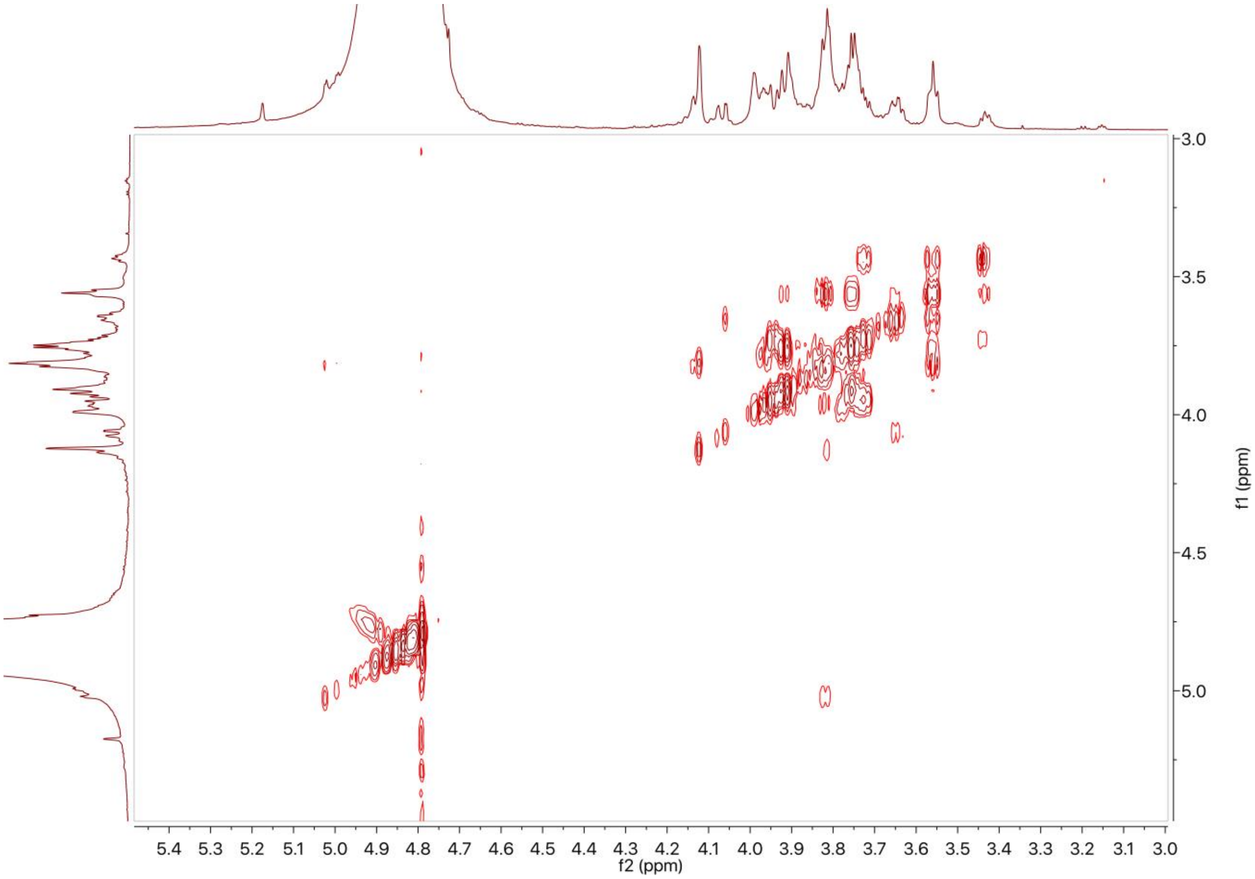

c. 6hex2 COSY

^{1}H NMR (800 MHz, Deuterium Oxide) δ 5.03, 5.00, 4.95, 4.75, 4.93, 4.77, 4.91, 4.78, 4.88, 4.85, 4.84, 4.81, 4.80, 4.12, 3.81, 4.08, 3.65, 4.06, 4.00, 3.98, 3.98, 3.78, 3.97, 3.78, 3.73, 3.95, 3.95, 3.73, 3.76, 3.56, 3.92, 3.76, 3.92, 3.56, 3.90, 3.89, 3.75, 3.87, 3.87, 3.55, 3.97, 3.84, 3.56, 5.02, 3.96, 3.84, 3.56, 4.13, 5.02, 3.82, 3.56, 3.98, 3.78, 3.92, 3.56, 3.76, 3.91, 3.93, 3.75, 3.72, 3.95, 3.44, 3.94, 3.44, 3.73, 3.68, 3.66, 4.07, 3.66, 4.06, 3.65, 3.44, 3.65, 3.56, 3.75, 3.82, 3.56, 3.56, 3.44, 3.81, 3.65, 3.44, 3.73, 3.56, 3.44

^{1}H NMR (800 MHz, Deuterium Oxide) δ 5.02, 5.00, 4.95, 4.93, 4.93, 4.91, 4.90, 4.89, 4.88, 4.85, 4.84, 4.81, 4.79, 4.12, 4.12, 4.08, 4.06, 4.06, 4.01, 3.99, 3.98, 3.97, 3.97, 3.96, 3.95, 3.95, 3.94, 3.94, 3.93, 3.93, 3.93, 3.91, 3.91, 3.91, 3.90, 3.89, 3.89, 3.88, 3.86, 3.84, 3.83, 3.83, 3.83, 3.83, 3.82, 3.82, 3.82, 3.81, 3.81, 3.81, 3.81, 3.78, 3.78, 3.77, 3.76, 3.76, 3.76, 3.74, 3.74, 3.73, 3.73, 3.73, 3.72, 3.71, 3.71, 3.69, 3.66, 3.66, 3.65, 3.64, 3.64, 3.57, 3.57, 3.57, 3.56, 3.56, 3.56, 3.55, 3.55, 3.55, 3.55, 3.55, 3.45, 3.45, 3.44, 3.44.

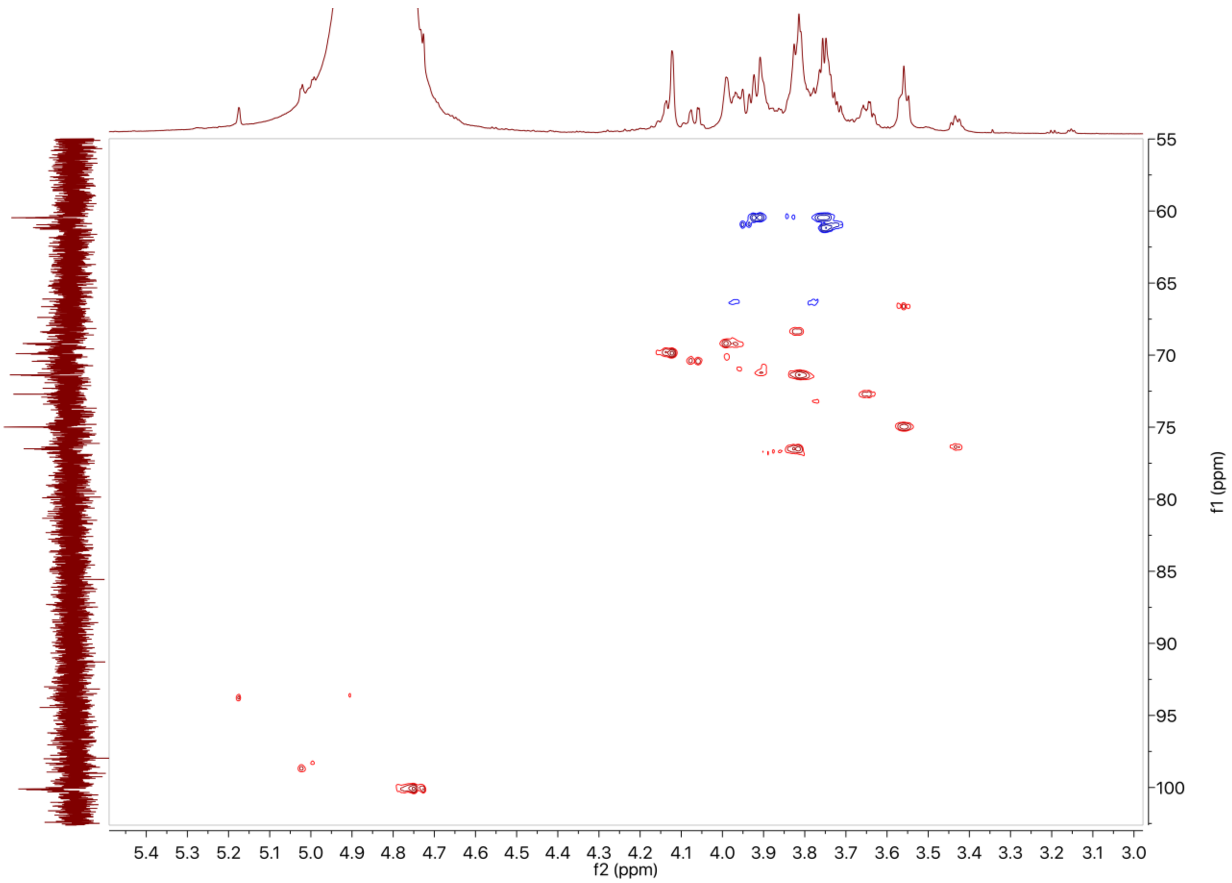

d. 6hex2 HSQC

^{13}C NMR (201 MHz, Deuterium Oxide) δ 93.76, 98.68, 98.28, 100.08, 100.12, 69.86, 70.38, 70.42, 69.19, 70.12, 66.31, 60.95, 60.91, 60.46, 60.45, 71.22, 68.36, 76.50, 71.37, 66.33, 73.20, 60.46, 61.17, 72.71, 66.61, 74.97, 76.38

^{1}H NMR (800 MHz, Deuterium Oxide) δ 5.18, 5.02, 5.00, 4.75, 4.73, 4.12, 4.08, 4.06, 3.99, 3.99, 3.97, 3.95, 3.94, 3.92, 3.91, 3.91, 3.82, 3.82, 3.81, 3.78, 3.77, 3.75, 3.75, 3.64, 3.56, 3.56, 3.43.

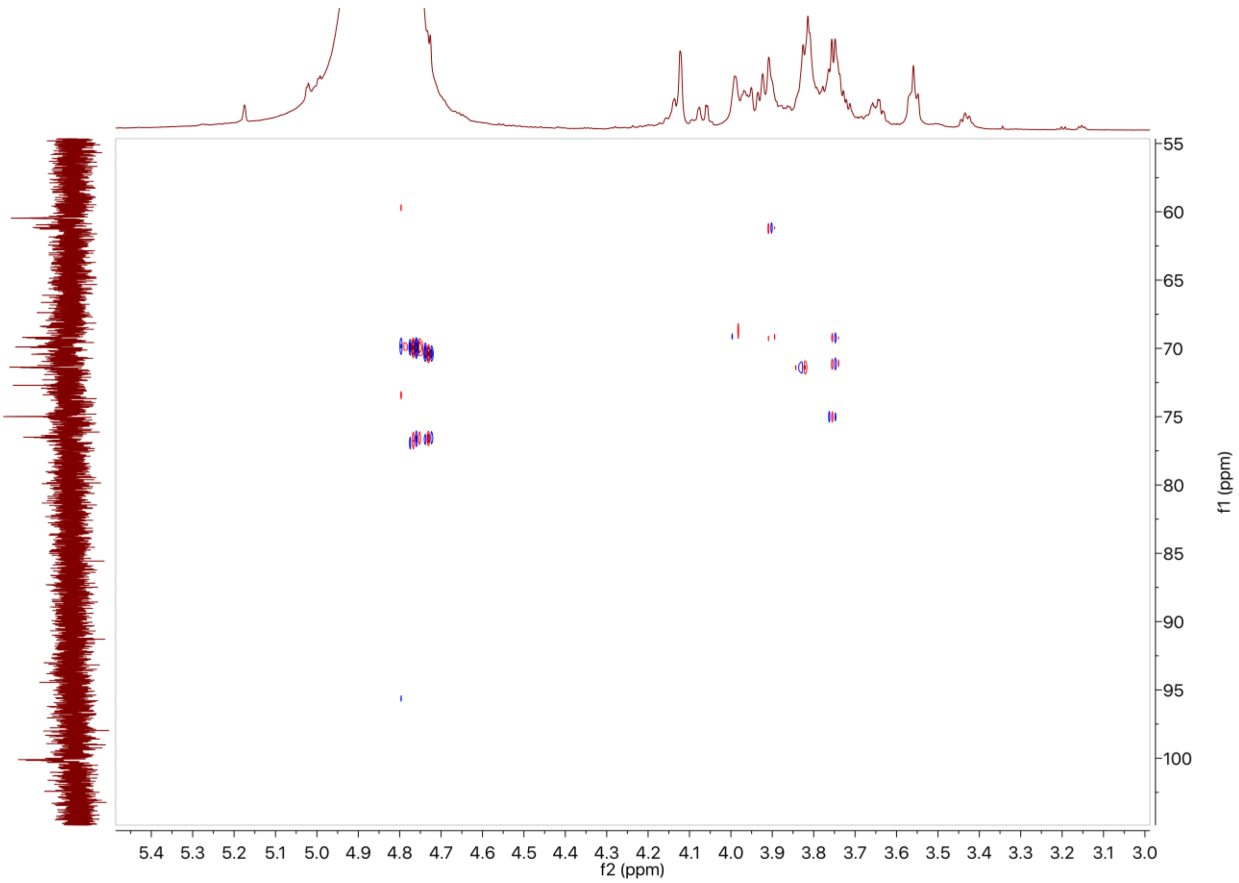

e. 6hex2 HMBC

^{13}C NMR (201 MHz, Deuterium Oxide) δ 69.90, 69.94, 76.76, 76.62, 69.93, 76.58, 69.90, 76.67, 70.29, 76.59, 70.40, 76.54, 70.39, 69.14, 68.60, 61.23, 61.18, 71.41, 71.40, 69.13, 71.39, 75.01, 71.16, 75.01, 69.23, 71.14, 75.01, 71.09

^{1}H NMR (800 MHz, Deuterium Oxide) δ 4.78, 4.77, 4.77, 4.76, 4.76, 4.75, 4.75, 4.74, 4.74, 4.73, 4.73, 4.72, 4.72, 4.00, 3.98, 3.91, 3.90, 3.84, 3.83, 3.82, 3.82, 3.76, 3.76, 3.75, 3.75, 3.75, 3.75, 3.74.

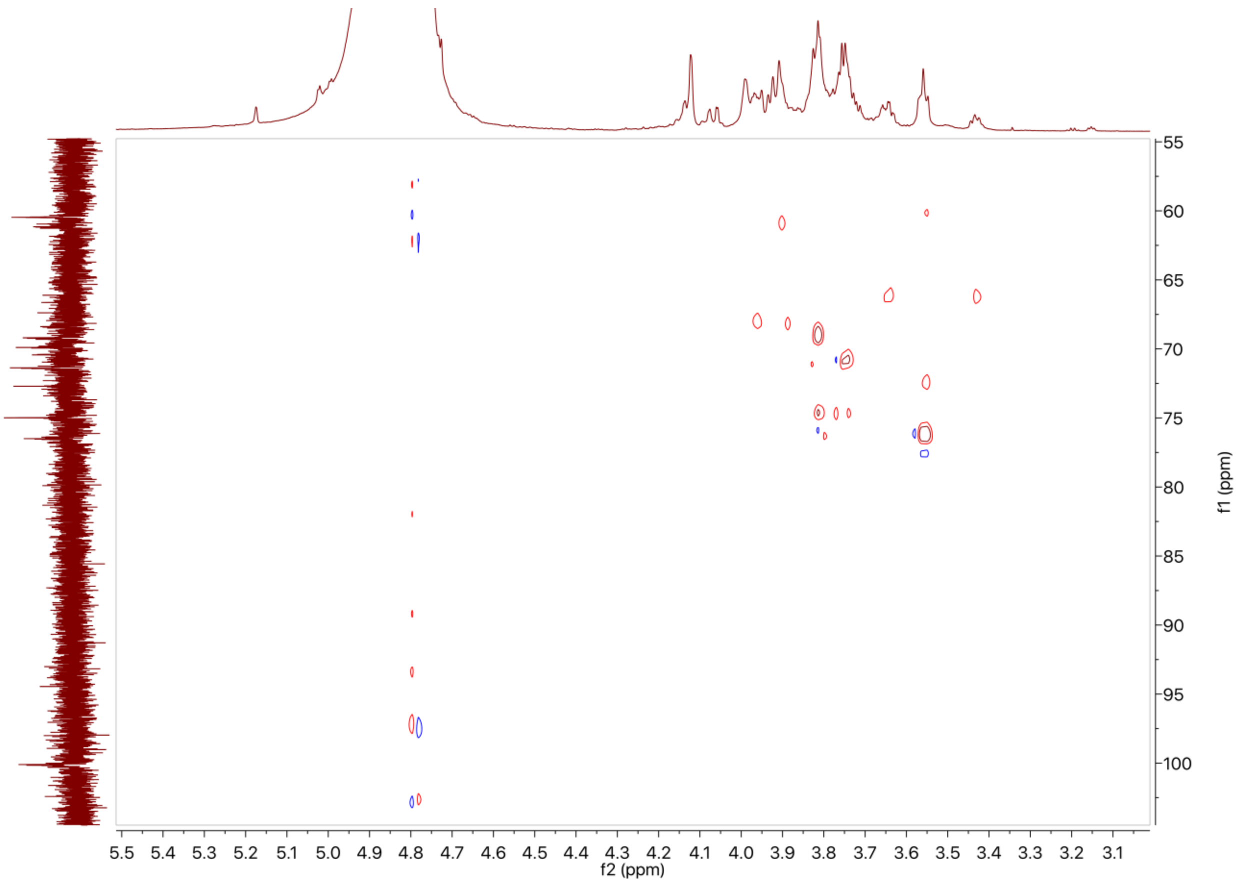

f. 6hex2 H2BC

^{13}C NMR (201 MHz, Deuterium Oxide) δ 68.01, 60.91, 69.00, 74.62, 70.80, 70.76, 74.64, 66.09, 76.19

^{1}H NMR (800 MHz, Deuterium Oxide) δ 3.96, 3.90, 3.81, 3.81, 3.77, 3.75, 3.74, 3.64, 3.56.

g. 6hex3 MS/MS
