# Supplementary material for: A nonenzymatic method for cleaving polysaccharides to yield oligosaccharides for structural analysis
Source: Nat Commun. 2020 Aug 7;11:3963. doi: 10.1038/s41467-020-17778-1 (PMC7414865; doi:10.1038/s41467-020-17778-1)
Supplement: Supplementary file 8 — Reporting Summary [file 41467_2020_17778_MOESM8_ESM.pdf]

nature research

Corresponding author(s): Carlito B. Lebrilla

Last updated by author(s): 07/03/2020

Reporting Summary

Nature Research wishes to improve the reproducibility of the work that we publish. This form provides structure for consistency and transparency in reporting. For further information on Nature Research policies, see our Editorial Policies and the Editorial Policy Checklist.

Statistics

For all statistical analyses, confirm that the following items are present in the figure legend, table legend, main text, or Methods section.

n/a	Confirmed	
☐	☒	The exact sample size (*n*) for each experimental group/condition, given as a discrete number and unit of measurement
☒	☐	A statement on whether measurements were taken from distinct samples or whether the same sample was measured repeatedly
☒	☐	The statistical test(s) used AND whether they are one- or two-sided<br>*Only common tests should be described solely by name; describe more complex techniques in the Methods section.*
☒	☐	A description of all covariates tested
☒	☐	A description of any assumptions or corrections, such as tests of normality and adjustment for multiple comparisons
☒	☐	A full description of the statistical parameters including central tendency (e.g. means) or other basic estimates (e.g. regression coefficient) AND variation (e.g. standard deviation) or associated estimates of uncertainty (e.g. confidence intervals)
☒	☐	For null hypothesis testing, the test statistic (e.g. *F*, *t*, *r*) with confidence intervals, effect sizes, degrees of freedom and *P* value noted<br>*Give P values as exact values whenever suitable.*
☒	☐	For Bayesian analysis, information on the choice of priors and Markov chain Monte Carlo settings
☒	☐	For hierarchical and complex designs, identification of the appropriate level for tests and full reporting of outcomes
☒	☐	Estimates of effect sizes (e.g. Cohen's *d*, Pearson's *r*), indicating how they were calculated

Our web collection on statistics for biologists contains articles on many of the points above.

Software and code

Policy information about availability of computer code

Data collection	LC-MS data was collected using Agilent Mass Hunter B.10. MALDI data was collected using Bruker FlexAnalysis 3.4.
Data analysis	Data was analyzed using Agilent Mass Hunter B.10. MALDI data was collected using Bruker Flex Analysis 3.4.

For manuscripts utilizing custom algorithms or software that are central to the research but not yet described in published literature, software must be made available to editors and reviewers. We strongly encourage code deposition in a community repository (e.g. GitHub). See the Nature Research guidelines for submitting code & software for further information.

Data

Policy information about availability of data

All manuscripts must include a data availability statement. This statement should provide the following information, where applicable:

- Accession codes, unique identifiers, or web links for publicly available datasets
- A list of figures that have associated raw data
- A description of any restrictions on data availability

The authors declare that the data supporting the findings of the study are available in the paper and its supplementary data files.

Field-specific reporting

Life sciences study design

All studies must disclose on these points even when the disclosure is negative.

Sample size	Samples did not involve biological comparisons and samples were analyzed with previously validated methods that have been shown to be precise, accurate, and robust. Data was validated through replication, however, due to the format and intent of the manuscript only a single point was shown for each analyses.
Data exclusions	No data was excluded in our analyses.
Replication	Attempts at replication were successful.
Randomization	Randomization was not used because sample were not compared with each other.
Blinding	As this paper was method development, blinding was not necessary.

Reporting for specific materials, systems and methods

We require information from authors about some types of materials, experimental systems and methods used in many studies. Here, indicate whether each material, system or method listed is relevant to your study. If you are not sure if a list item applies to your research, read the appropriate section before selecting a response.

Materials & experimental systems

n/a	Involved in the study	
☒	☐	Antibodies
☒	☐	Eukaryotic cell lines
☒	☐	Palaeontology and archaeology
☒	☐	Animals and other organisms
☐	☒	Human research participants
☐	☒	Clinical data
☒	☐	Dual use research of concern

Methods

n/a	Involved in the study	
☒	☐	ChIP-seq
☒	☐	Flow cytometry
☒	☐	MRI-based neuroimaging

Human research participants

Policy information about studies involving human research participants

Population characteristics	The fecal samples were collected from a healthy, term 6-month-old infant. who completed the UC Davis Infant Microbiome Nutrition and Development (Infant MiND) Study. The infant was exclusively breastfed at enrollment.
Recruitment	The infant was selected from the UC Davis Infant Microbiome Nutrition and Development (Infant MiND) Study. The infant was exclusively breastfed at enrollment.
Ethics oversight	The University of California Davis Institutional Review Board approved all aspects of this study and written informed consent was obtained from the participants (Protocol ID: 919505).

Note that full information on the approval of the study protocol must also be provided in the manuscript.

Clinical data

Policy information about clinical studies

All manuscripts should comply with the ICMJEguidelines for publication of clinical research and a completedCONSORT checklist must be included with all submissions.

Clinical trial registration	This study was registered on clinicaltrials.gov (NCT01817127).
Study protocol	clinicaltrials.gov (NCT01817127).
Data collection	The sample was collected by the mother at the time of defecation. The sample was stored in -80c until sample preparation.
Outcomes	No outcomes were measured.
