# Supplementary material for: A nonenzymatic method for cleaving polysaccharides to yield oligosaccharides for structural analysis
Source: Nat Commun. 2020 Aug 7;11:3963. doi: 10.1038/s41467-020-17778-1 (PMC7414865; doi:10.1038/s41467-020-17778-1)
Supplement: Supplementary file 1 — Peer Review File [file 41467_2020_17778_MOESM1_ESM.pdf]

Editorial Note: This manuscript has been previously reviewed at another journal that is not operating a transparent peer review scheme. The manuscript was considered suitable for publication without further review at *Nature Communications.*
